# *N*‑Glycoproteomic Portraits of *Bothrops* Snake Venoms Reveal Evolutionarily Conserved and Divergent Phenotypes

**DOI:** 10.1021/acs.jproteome.5c00249

**Published:** 2025-11-07

**Authors:** Débora Andrade-Silva, Lívia Rosa-Fernandes, Marcelo S. Reis, Alison F. A. Chaves, Dilza Trevisan-Silva, Silvia R. T. Cardoso, Giuseppe Palmisano, Martin R. Larsen, Solange M. T. Serrano

**Affiliations:** † Laboratory of Applied Toxinology, Center of Toxins, Immune-Response, and Cell Signaling (CeTICS), 196591 Butantan Institute, São Paulo 05503-900, Brazil; ‡ Department of Biochemistry and Molecular Biology, University of Southern Denmark, Odense DK-5230, Denmark; § Institute of Computing, Unicamp, Campinas 13083-889, Brazil; ∥ Biological Museum, Butantan Institute, São Paulo 05503-900, Brazil; ⊥ Department of Parasitology, Institute of Biomedical Science, University of São Paulo, São Paulo 05508-000, Brazil; # School of Natural Sciences, Macquarie University, Sydney, New South Wales 2109, Australia

**Keywords:** glycoproteomics, mass spectrometry, *N*-glycans, protein glycosylation, proteome, snake venom

## Abstract

Glycosylation is a major protein post-translational modification in snake venom proteins and contributes to the diversification of proteomes. In this study, we carried out an in-depth analysis of the glycosylation profile of seven *Bothrops* venoms, including neutral sugar quantification, glycoprotein profiling by lectin blot, and determination of *N*-glycosylation sites in proteins and their *N*-glycan compositions, by direct, intact glycopeptide analysis by mass spectrometry. Interestingly, all identified *N*-glycosylated peptides were from enzymatic venom components, mainly proteolytic enzymes that are key in envenomation. All venoms revealed a prominent occurrence of fucose and sialic acid in all *N*-glycosylated toxins identified. The results indicated that in *Bothrops* venoms, there is an important level of variation in protein primary structure that is not restricted to regions containing *N*-sequons. Overall, the signatures of *N*-glycosylated and nonglycosylated peptide backbones and of *N*-glycan site occupation by different *N-*glycans revealed conservation of venom phenotype framework and diversification of *N*-glycan usage. Hence, the molecular mechanisms of toxin structure and function evolution are at the same time dynamic in that they involve a fine-tuning for the presence of distinct glycans as an evolutionary novelty and are subjected to some conservation that results in the clustering of *Bothrops* venoms according to the species phylogenetic classification.

## Graphical abstract

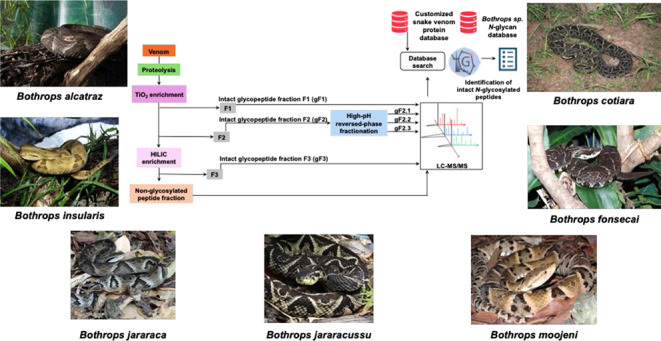

## 1 Introduction

Snake venoms are remarkable sources of biomolecules that have been explored for centuries; however, some of their structural features still require clarification. These complex secretions are the result of innovation and coevolution of an essential weapon arsenal for predation and self-defense, mainly composed of proteins and peptides.^1–3^ Venom proteome complexity is founded on the number of different protein classes, including enzymatic and nonenzymatic components, their proteoforms, and the dynamic range of concentration.^4–7^ Moreover, besides the wide nature of components, variability is another important feature of snake venoms and occurs at all taxonomical levels, with intraspecific variation occurring between individual specimens due to seasonal changes, diet, habitat, age, and sexual dimorphism, impacting envenomation outcome and treatment.^2,8–12^ Variability is a ubiquitous phenomenon that has been illustrated by different electrophoretic profiles, proteomes, and pharmacological activities between snake venoms.

In addition, another important factor affecting snake venom variability is protein glycosylation, a major co- and post-translational modification (PTM) that contributes to the diversification of proteomes and possibly plays a role in their distinct biological activities.^13–15^ Overall, glycosylation is recognized as a crucial modification that affects protein folding, solubility, resistance to proteolysis, structural stability, plasma half-life time, and immunogenicity.^16–19^ Although glycosylation has been extensively documented at several taxonomic levels in venom proteomes,^14,15,20–25^ as its biosynthesis is not template-driven, the regulation of this variability mechanism and the consequences for the venom proteome are poorly understood. In viperid venoms, glycosylation is characterized by different types of *N*-glycan structures, with different isomers, variable degrees of branching, sialylation, and fucosylation, increasing the proteome complexity.^15,20,23,26–28^ Furthermore, glycosylation may have provided a significant advantage for snakes using venom in predation, as it is critical for the maintenance of the proteome homeostasis within the venom gland by contributing to protein solubility and allowing the snake to make use of a highly concentrated toxic solution.^14,15^

Snakes of the *Viperidae* family, including the genera *Bothrops* (pit vipers) and *Crotalus* (rattlesnakes), cause most human accidents in North, Central, and South America.^29^ The *Bothrops* genus is responsible for more than 90% of the snakebite accidents reported in Brazil,^30^ and the general aspects of the envenomation include local edema and myonecrosis, local and systemic hemorrhage, severe coagulopathy, cardiovascular disorders, and renal failure.^31–34^ The medical importance of envenomation by *Bothrops* snakes indicates that the in-depth characterization of their venoms is crucial.

In this study, we focused on the interspecies venom variability of seven *Bothrops* snakes (*B. alcatraz*, *B. cotiara*, *B. fonsecai*, *B. insularis*, *B. jararaca*, *B. jararacussu*, and *B. moojeni*). *B. jararaca*, a mainland species that occurs in the most populated areas of Brazil,^35^ composes a group entitled the *Jararaca* Complex together with two critically endangered insular species, *B. alcatraz* and *B. insularis*, which originated from a *B. jararaca*-like ancestor.^36^ Their speciation process occurred after the geographic isolation process in the late Pleistocene,^37,38^ with *B. jararaca* evolving in a rich environment with different types of prey, while *B. insularis* and *B. alcatraz* were restricted to feeding on, respectively, birds and ectothermic prey. *B. cotiara* and *B. fonsecai*, which inhabit different areas of Araucaria forests in Brazil, are morphologically extremely similar and feed mainly on rodents.^39^ On the other hand, *B. jararacussu* and *B. moojeni* are classified in distinct positions in the phylogenetic tree of *Bothrops* species and share similar diets characterized by both ectothermic and endothermic prey.^39,40^ Hence, this study includes species placed in different branches of the *Bothrops* phylogenetic tree and with different feeding habits.^40,41^

The driving forces operating in shaping snake venom phenotypes upon evolution have been explored from different perspectives. In this study, we prospected *N*-glycosylation in *Bothrops* venom glycoproteins to evaluate the contribution of this PTM to the variability of venom proteomes. The glycosylation profiling was evaluated by neutral sugar content quantification, lectin blot probing different sugars, and identification of intact *N*-glycosylated peptides by mass spectrometry, resulting in the first comprehensive characterization of snake venom *N*-glycoproteomes. The integrated use of MS-driven analysis of *N*-glycan chains and peptide backbones allowed the identification of *N*-glycosylated sites in venom proteins and their roles in intraspecies venom complexity and variability.

## 2 Experimental Procedures

A detailed description of methodology is provided in Supporting Information Appendix 1. In brief, the venom neutral sugar content was quantified by the phenol-sulfuric method, and venom glycoprotein was profiled by lectin blot targeting different carbohydrates. Analysis of *N*-glycosylation in proteins was performed by proteolysis with trypsin allied to glycopeptide enrichment using TiO_2_ and hydrophilic interaction chromatography. Glycopeptide-enriched fractions were split into two parts for (i) enzymatic de-*N*-glycosylation and analysis by LC-MS/MS, and (ii) direct analysis of intact *N*-glycosylated peptides by LC-MS/MS. Combined information from a database of *N*-glycans of eight *Bothrops* venoms together with the peptide sequences identified in the de-*N*-glycosylated peptide fraction was used for a spectra search using the GlycReSoft software. Clustering analyses were performed using a Python-coded program in a Jupyter notebook, using the following libraries: matplotlib, numpy, pandas, seaborn, and scipy.

## 3 Results

### 3.1 *Bothrops* Venom Comparison Based on Electrophoretic Protein Profile and Carbohydrate Content Analysis

The protein profiles of venoms of *B. alcatraz*, *B. cotiara*, *B. fonsecai*, *B. insularis*, *B. jararaca*, *B. jararacussu*, and *B. moojeni* by SDS-PAGE, under reducing and nonreducing conditions (Figure S1), showed differences in molecular masses. In the first group, the venoms from *B. alcatraz*, *B. cotiara*, *B. fonsecai*, *B. insularis*, and *B. jararaca* showed, upon reduction of disulfide bonds, intense protein bands at ∼50 kDa, 20–25 kDa, and 12–15 kDa. Some less intense bands were also visualized at 50–250 kDa and 30–40 kDa. The protein profile of these five venoms under nonreducing conditions also showed similarities, as most bands migrated above 20 kDa, with the most intense and common band at 50 kDa. The second group, composed of the *B. jararacussu* and *B. moojeni* venoms, now shows the characteristic and intense protein band at 50 kDa present in the venoms of the other group. *B. jararacussu* venom showed a strongly stained band at 15 kDa under reducing conditions, while *B. moojeni* venom showed two intense protein bands at 55 and 60 kDa, which were not observed in the other venoms. Under nonreducing conditions, these two venoms showed protein bands distributed in the region above 15 kDa.

Previous studies on *Bothrops* venoms indicated that molecular mass variation may result from differences in protein length, oligomeric structure, and glycosylation levels of proteins.^14,28,42,43^ To further investigate the impact of glycosylation, we applied the phenol-sulfuric method for the determination of the total neutral carbohydrate content of these venoms (Figure S2 and Table S1); however, no significant differences were detected, as the values ranged from 0.03 to 0.04 μg carbohydrate/μg venom protein in all venoms.

### 3.2 Analysis of the Glycan Moiety of *Bothrops* Venom Glycoproteins by Lectin Blot

Our previous *N*-glycomic analysis^15^ revealed significant differences in the *N*-glycan compositions among *Bothrops* venoms. To further explore this feature, we used five digoxigenin-labeled lectins to detect different carbohydrate structures in venom glycoproteins by blot.

To approach the sialic acid units capping the glycan chains, we tested the lectins MAA and SNA, which preferentially bind to sialic acid attached to terminal galactose, respectively, in α-2,3 and α-2,6 linkages. Although at different intensities, the recognition of protein bands by MAA (Figure S3) suggested a wide occurrence of sialic acid in an α-2,3 linkage to a galactose unit in *N*-glycans of the venoms from *B. alcatraz*, *B. cotiara*, *B. fonsecai*, *B. insularis*, and *B. jararaca*. In these venoms, the lectin detection was similarly restricted to proteins of ∼30 kDa and above. Notably, the recognition profiles of *B. alcatraz*, *B. insularis*, and *B. jararaca* were more intense than the venoms of *B. cotiara* and *B. fonsecai*. On the other hand, glycoproteins in the venoms *B. jararacussu* and *B. moojeni* were not recognized. The sialoglycoprotein probing using the lectin SNA (Figure S4) resulted in no recognition in all venoms, as detected by lectin blot.

The DSA lectin recognizes GlcNAc oligomers and the disaccharide Gal-1,4-GlcNAc,^44^ and here, the pattern of venom glycoproteins recognized by this lectin (Figure S5) revealed significant differences and clustered the seven *Bothrops* venoms, similarly as observed by SDS-PAGE (Figure S1). The venoms of *B. alcatraz*, *B. cotiara*, *B. fonsecai*, *B. insularis*, and *B. jararaca* showed a similar recognition pattern. Although with different intensities, the glycoproteins detected were restricted to the mass range of ∼37–100 kDa. Another remarkable feature was the nonstained region at ∼50 kDa in the venoms from *B. alcatraz*, *B. cotiara*, *B. fonsecai*, *B. insularis*, and *B. jararaca*, which corresponds to a strong protein band in these venoms, as visualized by the Ponceau staining (Figure S5). This result indicates that this prominent band does not contain glycoproteins with GlcNAc oligomers and Gal-1,4-GlcNAc in these five venoms. On the other hand, the recognition of the venom proteins from *B. jararacussu* and *B. moojeni* by the lectin DSA showed a very distinct pattern, with glycoproteins stained in the region of 20–250 kDa. The venom of *B. jararacussu* showed a more spread-out pattern of stained bands in this molecular mass range, while in the profile of *B. moojeni* venom, the recognized proteins were concentrated at 40–150 Da. Interestingly, the analysis of the protein profile of these venoms under reducing conditions (Figure S1) showed a few bands of high molecular mass very weakly stained by Coomassie blue, which, however, showed high intensity in the lectin blot using DSA, suggesting that the glycoproteins recognized by DSA are low abundant.

The GNA lectin recognizes mannose units, and our previous study on *N*-glycan structures of glycoproteins of some *Bothrops* venoms revealed that those belonging to high-mannose type are conserved in glycoproteins of *B. alcatraz*, *B. cotiara*, *B. insularis*, *B. jararaca*, *B. erythromelas*, *B. moojeni*, *B. neuwiedi*, and *B. jararacussu* venoms.^15,24^ Differently from the diverse patterns observed with the DSA lectin, the detection using GNA showed rather similar venom profiles (Figure S6). Despite some variation in intensity, in all venoms, two protein bands of ∼50 and 150 kDa were recognized by GNA, while the venoms from *B. cotiara* and *B. jararaca* also showed a protein band of ∼25 kDa. Interestingly, *B. jararaca* and *B. insularis* venoms also showed some weak bands recognized by GNA in the regions of 150–50 and 50–25 kDa.

The PNA lectin, which recognized a few proteins in *Bothrops* venoms^14^ was used here to investigate the presence of the structure Gal-1,3-GalNAc. Accordingly, the blot detection using this lectin showed only weak protein bands of ∼12 kDa in *B. jararacussu* and *B. moojeni* venoms (Figure S7).

### 3.3 Strategy for Identification of *N*-Glycosylation Sites in *Bothrops* Venom Proteins

In this study, the experimental design for *N*-glycosylation site identification in *Bothrops* venom proteins included a combination of strategies to analyze intact *N*-glycopeptides and their corresponding de-*N*-glycosylated counterparts (Figure 1 and Table S2). To this end, *Bothrops* venom proteins were submitted to trypsinization, followed by enrichment of sialylated *N*-glycopeptides using TiO_2_ beads,^45^ from which two different fractions of glycopeptides (F1 and F2) were eluted. Fraction F1 was enriched in glycopeptides that were retained by hydrophilicity, while F2 contained glycopeptides retained due to their negative charges, mainly sialylated ones. The TiO_2_ nonbound fraction was submitted to a second step of glycopeptide enrichment using HILIC, and the bound fraction, F3, was collected. The three enriched glycopeptide fractions (F1, F2, and F3) were split into two parts for further analysis, with 90% of each sample being submitted to intact glycopeptide analysis by LC-MS/MS (fractions gF1, gF2, and gF3). As a higher number of glycopeptides was expected in gF2 because of the abundance of sialic acid units in the *N*-glycan chains of *Bothrops* venom glycoproteins,^15,46^ it was further fractionated by high-pH reversed-phase chromatography, and the three obtained fractions (gF2.1, gF.2.2, and gF2.3) were analyzed separately. The remaining 10% of each sample was subjected to enzymatic de-*N*-glycosylation using PNGase F (dF1, dF2, and dF3) followed by LC-MS/MS analysis. Likewise, the HILIC nonbound fraction, which likely contained nonglycosylated peptides, was quantified and subjected to LC-MS/MS analysis.

**Figure 1:**
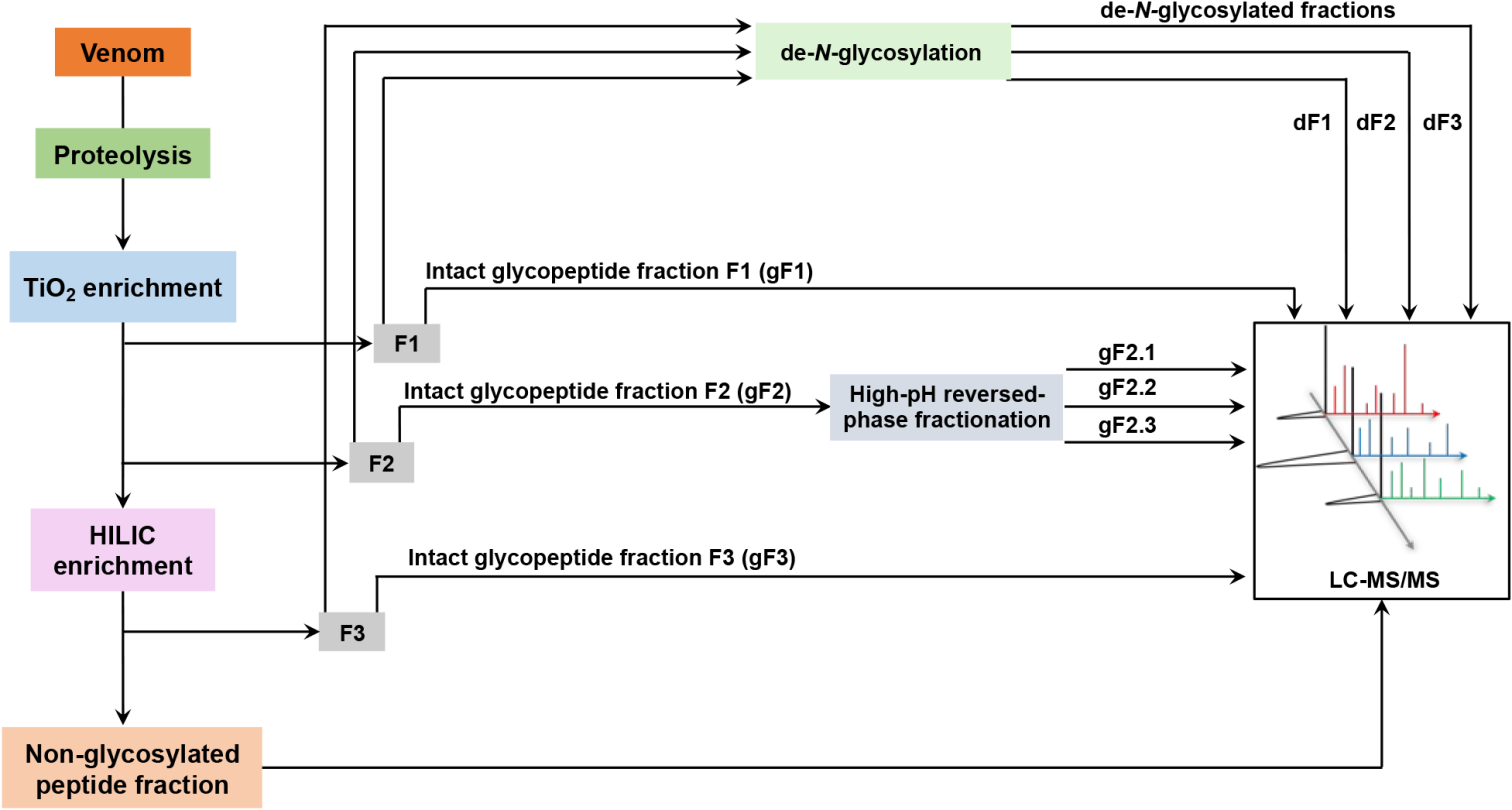
Scheme of the workflow used for the identification of glycopeptides in *Bothrops* venom proteins.

This approach facilitated the identification of intact *N*-peptides because the putative *N*-glycosylated peptides identified in the de-*N*-glycosylated samples were used to compose a restricted peptide database to assist in the intact *N*-glycopeptide data analysis (Figure 2). For this purpose, the spectral files from de-*N*-glycosylated fractions were submitted to a database search using MaxQuant,^47^ and the results are described in Tables S3–9.

**Figure 2:**
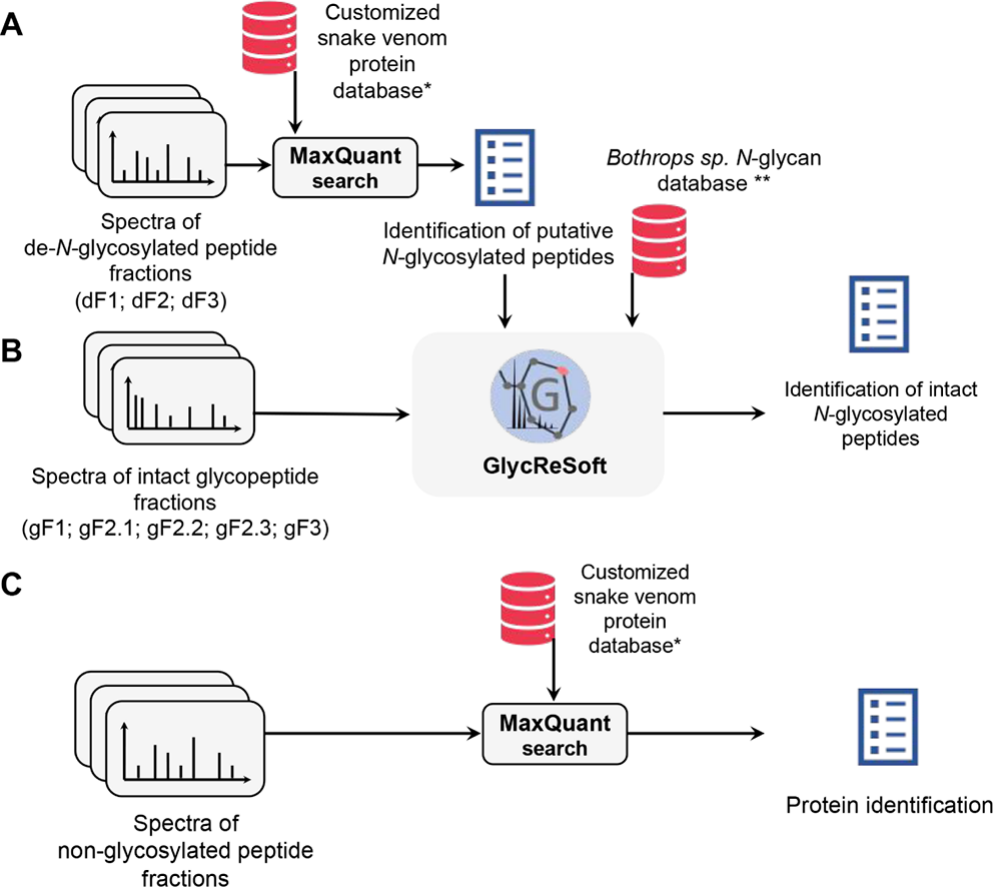
Scheme of the workflow of data analysis of de-*N*-glycosylated peptides, intact glycopeptides, and nonglycosylated peptides. (A) Data analysis workflow of de-*N*-glycosylated peptides. (B) Data analysis workflow of intact glycopeptide peptides. (C) Data analysis workflow of the nonglycosylated peptides (ref 24, *; ref 15, **).

### 3.4 Identification of *N*-Glycosylation Sites in *Bothrops* Venom Proteins by the Analysis of de-*N*-Glycosylated Peptides

The LC-MS/MS analysis of the de-*N*-glycosylated fractions (dF1, dF2, and dF3) showed consistent extracted ion chromatograms and distribution of peptide *m*/*z* values among the technical replicates (Figure S8) and revealed low numbers of missed trypsin cleavages (Figure S9). Furthermore, the three technical replicates shared ∼50% of the identified peptides (Figure S10), and the analysis of the peptides identified in each fraction (Figure S11) showed that dF3 contained the highest number of identified peptides, while the number of shared peptides in dF1, dF2, and dF3 varied between the venoms, and interestingly, dF3 contained the highest number of exclusive peptides in all venoms. After this comparison, all results were combined, and peptides identified in at least two replicates as deamidated and containing at least one canonical *N*-glycosylation sequon (NXS/T, where X is any amino acid except Pro) were considered putative *N*-glycosylated peptides (Figure S12 and Tables S3–9). Even though we considered only the canonical form of *N*-glycosylation sites in our analysis, some variants have been described, such as the sequence NXC/V and the reverse form of the canonical *N*-glycosylation site S/TXN.^48–54^ Although a few deamidated peptides containing noncanonical forms of glycosylation sites were identified (Tables S3–9), these were not considered as being putatively *N*-glycosylated peptides. Figure S13 summarizes the variable number of identified peptides in the de-*N*-glycosylated fractions of the seven venoms and their classification as nonmodified peptides, deamidated peptides, and putative *N*-glycosylated peptides. The main difference found between the venoms was the total number of identifications, while the proportion of putative *N*-glycosylated, deamidated, and nonmodified peptides was similar in all venoms (Figures S10 and S11).

Further, we selected the peptides containing a deamidated asparagine inserted in the canonical *N*-glycosylation sequon (NXS/T), considered here as putative formerly *N*-glycosylated peptides, and used them to compose a database for the intact *N*-glycosylated peptide identification (Figure 2and Tables S3–9). The term “putative” is applicable here since only the analysis of intact *N*-glycopeptides results in robust evidence of *N*-glycosylation occurrence.^55^ The total number of formerly *N*-glycosylated peptides varied from 90 (*B. fonsecai*) to 203 (*B. insularis*) (Table S10). UpSet plots were used to visualize the intersections of identified formerly *N*-glycosylated peptides in the different venoms (Figure S14) and showed that most of them were exclusively found in specific venoms. Also, *B. cotiara* and *B. fonsecai* venoms showed lower numbers of exclusive putative formerly *N*-glycosylated peptides, and only 17 were identified as occurring in the seven venom species, indicating a high level of variability in protein glycosylation in *Bothrops* venoms.

Finally, the numbers of identified putative *N*-glycosylation peptides and sites were compared. Because of the features of the bottom-up approach, the numbers of putative *N*-glycosylated peptides described so far contained a significant level of redundancy. This redundancy originated from missed cleavages, partial identification of semitryptic peptides, and the identification of many peptides of different lengths containing the same *N*-glycosylation site. The latter may be caused by *N*-glycosylation sites shared by different toxins with high identity or products of the proteolytic processing of toxins. Even though these peptides could arise from different proteins, redundancy removal is necessary to analyze *N*-glycosylation events at the toxin class level. To describe the *N*-glycosylation sites covered by the identified peptides, we used the software PatternLab^56^ to remove subset sequences with 100% identity (Tables S11–17). As a result, the number of putative *N*-glycosylation sites corresponded to around 52–77% of the number of identified putative *N*-glycopeptides in all venoms (Figure S15), indicating the occurrence of proteoforms.

### 3.5 Identification of *N*-Glycosylation Sites in *Bothrops* Venom Proteins by the Analysis of Intact *N*-Glycosylated Peptides

The data analysis of the intact glycopeptide fractions (gF1, gF2.1, gF2.2, gF2.3, and gF3) was based on searches using the GlycReSoft software package^57^ (Figure 2and Tables S18–24). Figure 3A–C summarizes the results of the analyses of intact *N*-glycopeptides, including the numbers of *N*-glycosylated peptides, peptide sequences, occupied *N*-glycosylation sites, and *N*-glycan compositions identified in *Bothrops* venoms. In this analysis, *B. jararacussu* and *B. moojeni* venoms resulted in the highest numbers of identified *N*-glycopeptides, respectively, 328 and 327, while *B. fonsecai* venom resulted in the lowest number (129). Nevertheless, the total number of different *N*-glycan compositions was quite similar for all venoms.

**Figure 3:**
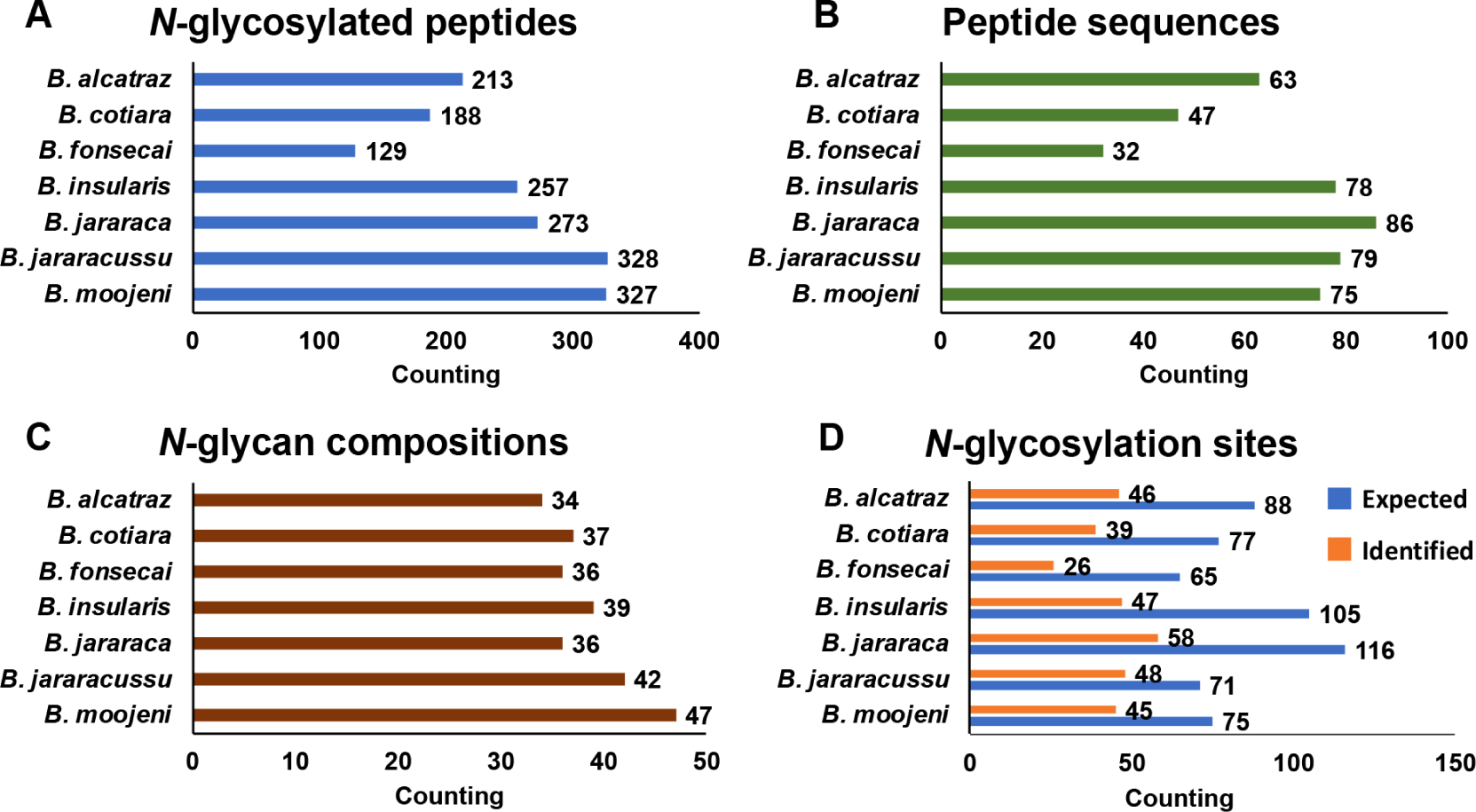
Comparison of the *N*-glycosylation features identified by analysis of intact *N*-glycopeptides of *Bothrops* venoms. (A) *N*-glycopeptides, as defined by a peptide backbone and an *N*-glycan composition. (B) Peptide sequence backbones. (C) *N*-glycan compositions. (D) Comparison of the number of *N*-glycosylation sites identified in the de-*N*-glycosylated peptide fractions (expected) and the *N*-glycosylation sites identified in the intact *N*-glycosylated peptide fractions (identified).

Considering the number of *N*-glycosylation sites identified in the analysis of de-*N*-glycosylated fractions (Figure S15) and the fact that peptides covering these sites were used as a database source for the intact *N*-glycopeptide database search, we expected to identify most of them in their intact glycopeptide forms. However, the number of *N*-glycosylation sites identified in the intact *N*-glycosylated peptide analysis varied between species, so that 40–67% of the expected sites were identified (Figure 3D). On the other hand, the analysis of identified *N*-glycosylation sequons, such as NXS and NXT, in each venom (Figure S16) showed a similar profile, with the number of sites containing NXT being slightly higher than that of NXS.

Further, the analysis of the distribution of *N*-glycosylated peptides identified in the intact glycopeptide fractions according to toxin class revealed that in all venoms metalloproteinases (SVMPs) and serine proteinases (SVSPs) showed the highest proportion of identified *N*-glycosylated peptides (Figure S17). Interestingly, while in the venoms of *B. alcatraz*, *B. insularis*, *B. jararaca*, *B. moojeni*, and *B. jararacussu* most of the identified *N*-glycosylated peptides were from SVMPs, in *B. cotiara* and *B. fonsecai* venoms, the majority of identified *N-*glycosylated peptides were from SVSPs. Moreover, a high number of identifications of *N-*glycosylated peptides was assigned to other low abundant glycoproteins in all venoms (Figure S17).

The next level of analysis of identified intact *N*-glycosylated peptides focused on the general distribution of *N*-glycan features among the venoms and toxins. Interestingly, the analysis of identified *N*-glycan compositions, classified as high-mannose, fucosylated, sialylated, and fucosylated and sialylated, revealed profiles of *B. cotiara*, *B. fonsecai*, *B. jararacussu*, and *B. moojeni* venoms containing a relatively higher proportion of fucosylated forms than venom of the Jararaca complex (*B. alcatraz*, *B. insularis*, and *B. jararaca*) (Figure S18A). In the case of the occupation of glycosites in SVMPs and SVSPs, most of them are populated by fucosylated and sialylated *N*-glycans while those of the high-mannose class are rather rare (Figure S18B). In agreement with our previous *N*-glycomic analysis,^15^ most of the *N*-glycan chains identified in the intact *N*-glycosylated peptide fraction belong to the hybrid/complex type (Figure S19).

Considering only the *N*-glycan compositions of the hybrid/complex type identified in the intact *N*-glycosylated peptide analysis, the number of sialic acid units per composition was a distinct feature between the venoms (Figure 4). In agreement with our previous *N-*glycomic analysis, the venoms of *B. alcatraz*, *B. cotiara*, *B. fonsecai*, *B. insularis*, and *B. jararaca* showed a higher number of *N*-glycan compositions of the hybrid/complex type, containing more than three sialic acid units per composition. In contrast, the analysis of *B. jararacussu* and *B. moojeni* venoms showed mostly hybrid/complex *N*-glycans with one or two sialic acid units. This analysis indicated a higher amount of sialic acid residues in *N*-glycans of SVMPs and SVSPs and overall variations in the content of sialic acid attached to the different toxin classes among the venoms.

**Figure 4:**
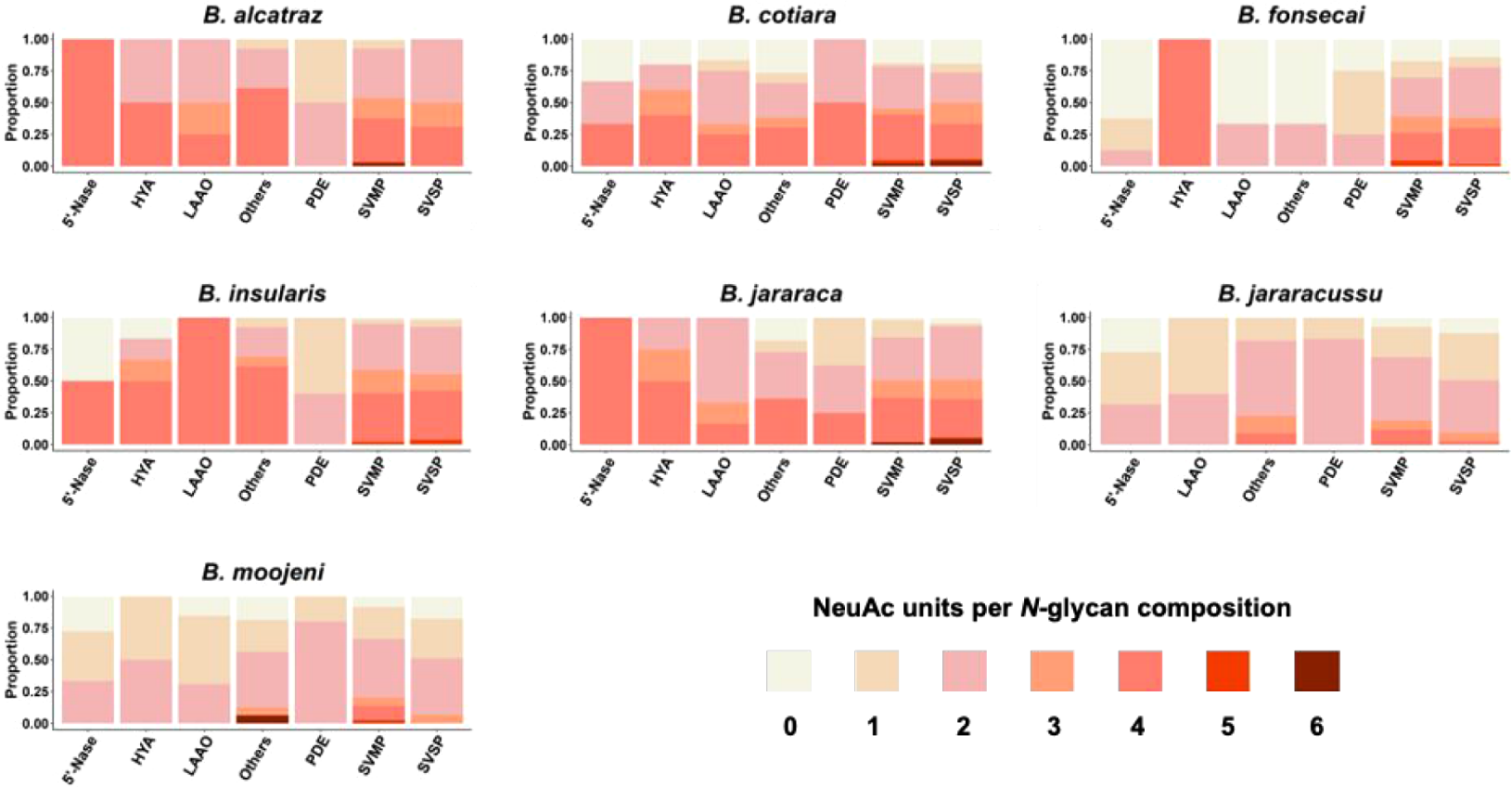
Distribution of identified intact *N*-glycosylated peptides containing *N*-glycans of hybrid/complex types by toxin class, and the proportion of these identifications according to the number of sialic acid units per *N*-glycan chain.

Sialic acid units confer a negative charge to *N*-glycan chains and may also undergo modifications, such as *O*-acetylation, which was already described in an *N*-glycosylated SVSP.^58^ In our analysis, we considered the possibility of the sialylated structures being *O*-acetylated by adding 42 Da to the mass of the unmodified sialylated *N*-glycan in the database. The summary of the *O*-acetylated *N*-glycosylated peptides identified in this study is summarized in Table S25 and shows that a small proportion of the *N*-glycans were modified, varying between 9 and 22% among the venoms. The analysis of some of the MS2 spectra of these identifications indicated the presence of the diagnostic ions 316.103^+1^ and 334.113^+1^ for Neu5Ac_2_ (Figure S20). However, GlycReSoft software did not detect the localization of the *O*-acetyl group on NeuAc in all identifications, and some were not annotated on the Neu5Ac units.

Overall, the *N*-glycoproteomic analysis of *Bothrops* venoms confirmed the significant differences in the *N*-glycosylation protein process regarding the generation of hybrid/complex *N*-glycans previously reported by *N*-glycomic analysis, which indicated clear differences in the glycoproteins of *Bothrops* venoms related to distinct antenna structures.^15^ Here, we searched for signature ions in the MS/MS spectra of intact *N*-glycopeptides that could confirm the presence of different antenna configurations in *Bothrops* venom proteins. As an example, Figure S21 shows the low mass range of the MS/MS spectra of three *N*-glycopeptides of *B. cotiara*, *B. jararaca*, and *B. jararacussu* venoms that share the same peptide backbone (of the catalytic domain of SVMPs) but are modified by different *N*-glycan chains. The antenna structure found in the *B. cotiara* and *B. jararaca* *N*-glycans of hybrid/complex type was composed of (NeuAc-2,8-NeuAc-2,3-Gal-1,4-GlcNAc), and oxonium ions derived from this structure could be annotated in some MS/MS spectra of intact *N*-glycopeptides, as exemplified in Figure S21. In the case of *B. cotiara* and *B. jararaca* venoms, the sialic acid dimer structure was revealed by series *m*/*z* 583, 565, and 547. The first ion represents the sialic acid dimer, and the subsequent ions are formed by its dehydration (loss of one and two water molecules). Conversely, these reporter ions were absent in the spectrum of the *N*-glycopeptide of *B. jararacussu* venom (Figure S21).

Moreover, as an alternative strategy to identify intact *N-*glycopeptides, we initially considered the possibility of using a species-specific *N*-glycan database for the database search instead of a compiled list of all *N*-glycan compositions of *Bothrops* venoms as reported in our previous study.^15^ This is an important aspect of analysis because, as a result of that study, the identified *N*-glycan compositions of *Bothrops* venoms clustered in three different groups. Therefore, considering that no *N*-glycan composition of *B. fonsecai* venom was available in our database, here, for the search of spectra of intact *N*-glycosylated peptides we decided to use the combined data of the eight *Bothrops* venom *N*-glycans described in that previous study for all species, including some *N*-glycan compositions exclusively found in specific venoms.

Overall, the distribution of the ten most frequent *N*-glycan compositions identified in the present study agrees with the previous *N*-glycan analysis, as summarized in Figure S22. The two compositions belonging to the high mannose class were found in all venoms with similar frequencies, and the group specific most abundant compositions from the previous *N*-glycome study were found here in their corresponding species (compositions B11, B22, and B32). As expected, the venom of *B. fonsecai* showed an overall profile closer to that of *B. cotiara* venom than to those of the other venoms. Nevertheless, it is important to mention that some *N*-glycan compositions identified in all the species were not expected, as these compositions were described as belonging to a different group in the previous *N*-glycome analysis.^15^ However, the present study does not elucidate the structure of the identified *N*-glycans, and all of the proposed structures are suggestions based on our *N*-glycome database, while the assigned compositions identified here may represent different structures that were not detected previously. Regarding the most common high-mannose composition found in *Bothrops* venoms (composition A1; Figure S22), a word cloud analysis of its presence in venom proteins showed an overall variation in frequency among the toxin classes and its high prevalence in the SVMPs (Figure S22).

### 3.6 *N*-Glycosylation Heterogeneity in *Bothrops* Toxins

The process of protein glycosylation is heterogeneous by nature. Although our analysis does not allow for a precise evaluation of micro- and macro-heterogeneities of glycosylation in *Bothrops* toxins, we interpreted our results as an estimation of *N*-glycan diversity occurring at the toxin class level in the analyzed venoms. To this end, a heterogeneity ratio was calculated using the number of *N*-glycan compositions as a numerator and the number of *N*-glycosylation sites identified in intact *N*-glycosylated peptides as a denominator, as proposed by Riley et al.^59^ A high ratio indicated that many *N*-glycoforms were identified, and consequently, these *N*-glycosylation sites showed higher heterogeneity. Tables S26 and S27 contain the calculated values, and Figure 5 shows the heterogeneity ratios calculated for each species, respectively, in LAAOs, SVMPs, and SVSPs, and in SVMP domains. The global average ratios of each venom, considering the glycosylation sites identified in all toxin classes, varied from 1.8 to 3.5 *N*-glycoforms per *N*-glycosylation site (Table S26). As there is no venom data for comparison, we used the average ratio obtained by Riley et al.^59^ for the mouse brain tissue as a reference. While in the mouse brain the ratio average was 1.56, *Bothrops* venoms showed a general ratio of 2.6, which is in accordance with the fact that venom proteomes are composed of secreted proteins occurring in many proteoforms.^5,60–62^ Similarly to the glycosylation signatures observed in Figures 3 and 4, the heterogeneity ratios of toxin classes revealed distinct profiles between the venoms. Interestingly, the average ratios displayed by the different toxin classes in all species showed that some nonabundant components, such as LAAO, APase, and PDE, exhibited the highest values (Table S26).

**Figure 5:**
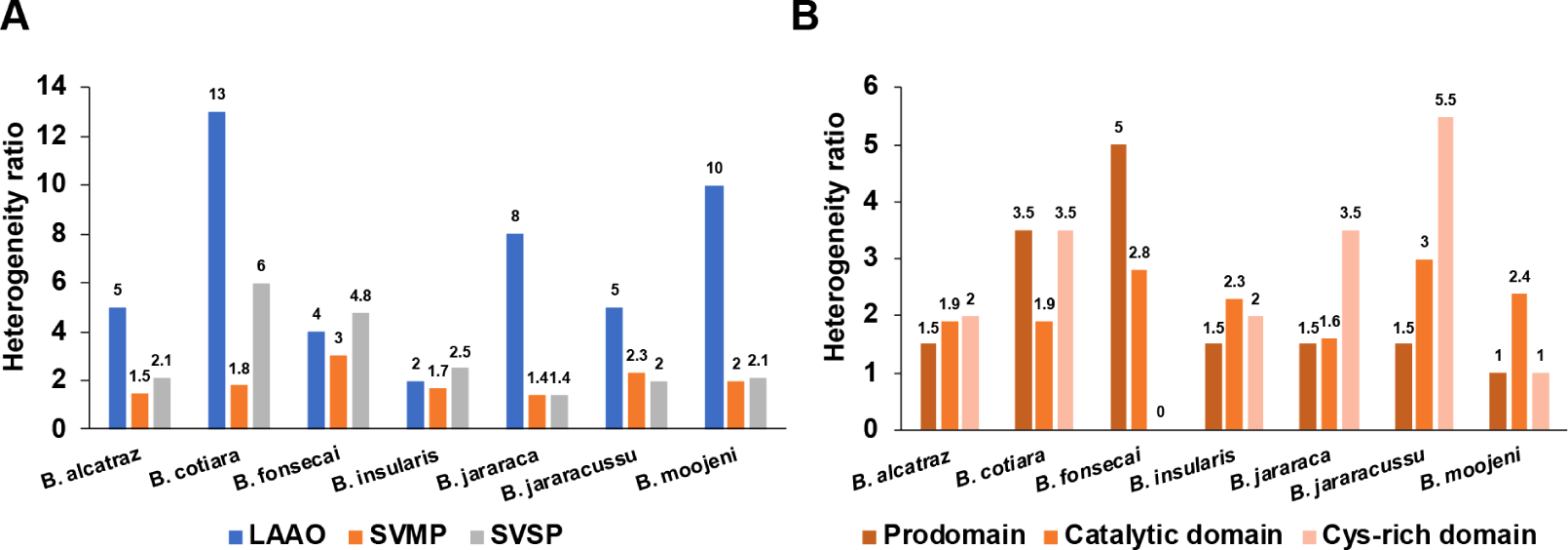
Comparison of the heterogeneity ratio in the *N*-glycosylated toxin classes identified in the intact *N*-glycopeptide analysis. (A) Analysis of the SVMP, SVSP, and LAAO toxin classes. (B) Analysis of the heterogeneity ratio in the SVMP domains. The heterogeneity ratio was calculated by dividing the number of *N*-glycan compositions by the number of *N*-glycosylation sites identified in intact *N*-glycosylated peptides.

Regarding the most abundant glycosylated toxins in *Bothrops* venoms, SVMPs and SVSPs, the latter showed a higher heterogeneity ratio (Figure 5). Comparatively, *B. cotiara* venom SVSPs showed the highest ratio, and *B. jararacussu* venom exhibited the lowest ratio. Considering the heterogeneity within SVMP domains, we observed different patterns among the seven species. While glycosylation in SVMPs of *B. alcatraz* and *B. insularis* venoms showed close heterogeneity ratios in the pro, catalytic, and Cys-rich domains, in *B. fonsecai* venom the prodomain/catalytic domain showed the highest ratio. In *B. moojeni* venom, the highest ratio was detected in the catalytic domain, and in *B. jararaca* and *B. jararacussu* venoms, the Cys-rich domain showed the highest heterogeneity. In *B. cotiara* venom, the prodomain and Cys-rich domain showed heterogeneity higher than that of the catalytic domain. Surprisingly, we did not identify any glycosylated peptide in the disintegrin domain, indicating that these *N*-glycosylation sites may not be occupied often or be present in low abundance.

Considering the SVMP class, we also analyzed the *N*-glycan compositions identified in each structural domain. Figure 6A shows *N*-glycosylation sites identified as occupied by *N*-glycan chains, which, for illustration, were positioned in the primary structure of a typical P–III SVMP precursor and the distribution of the features of the identified *N*-glycans, indicating that in all venoms and all domains most *N*-glycans are fucosylated and sialylated.

**Figure 6:**
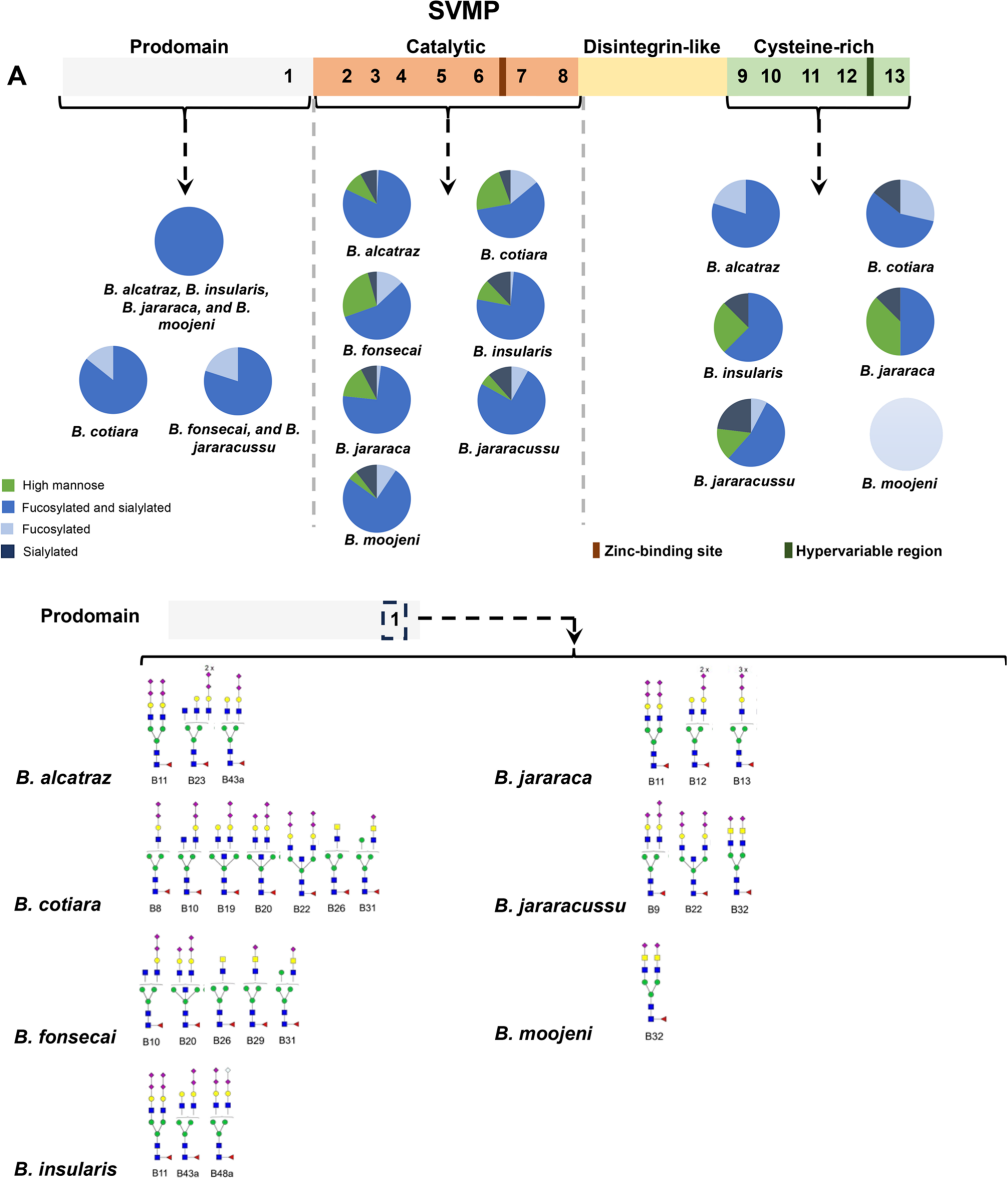

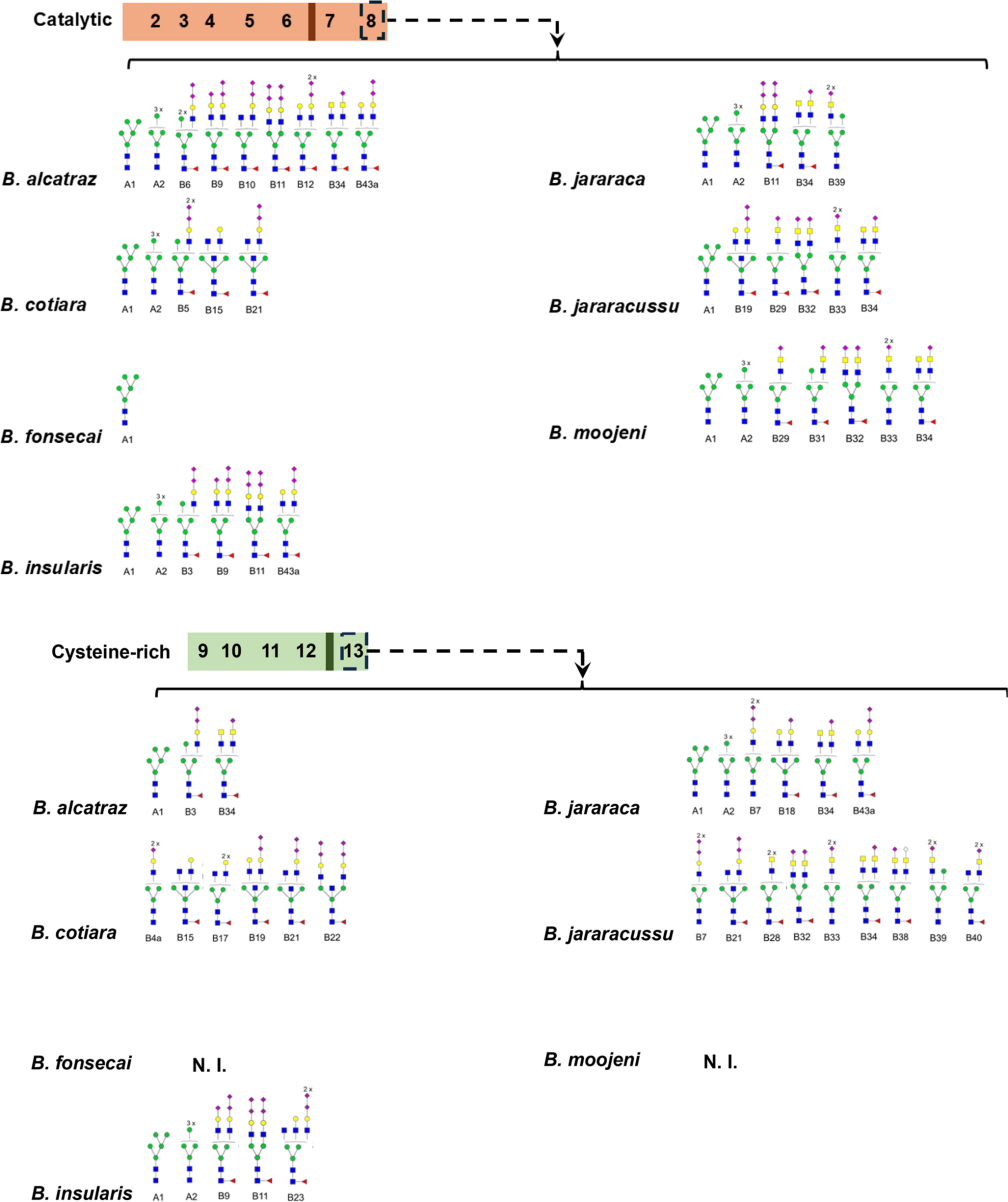

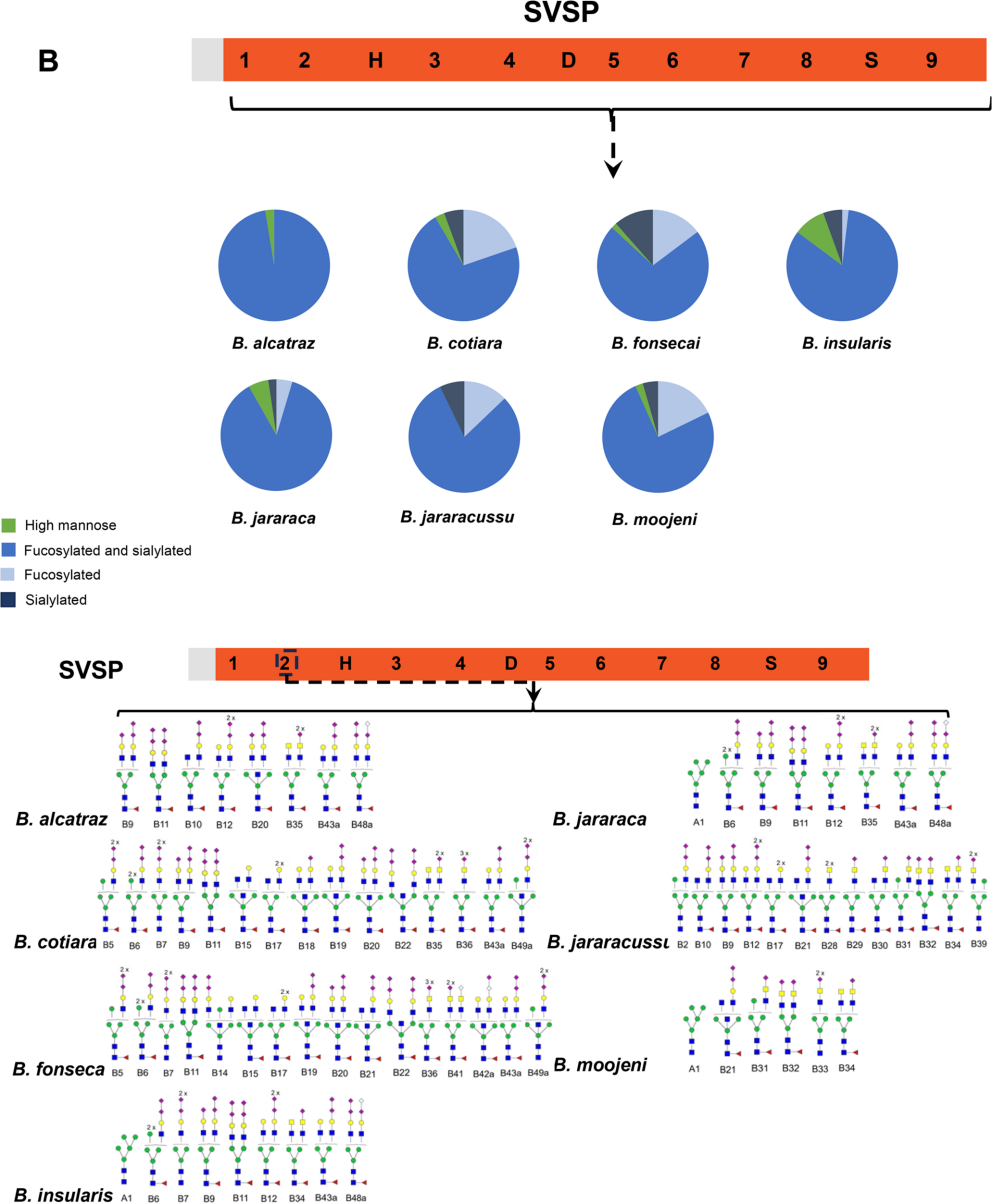

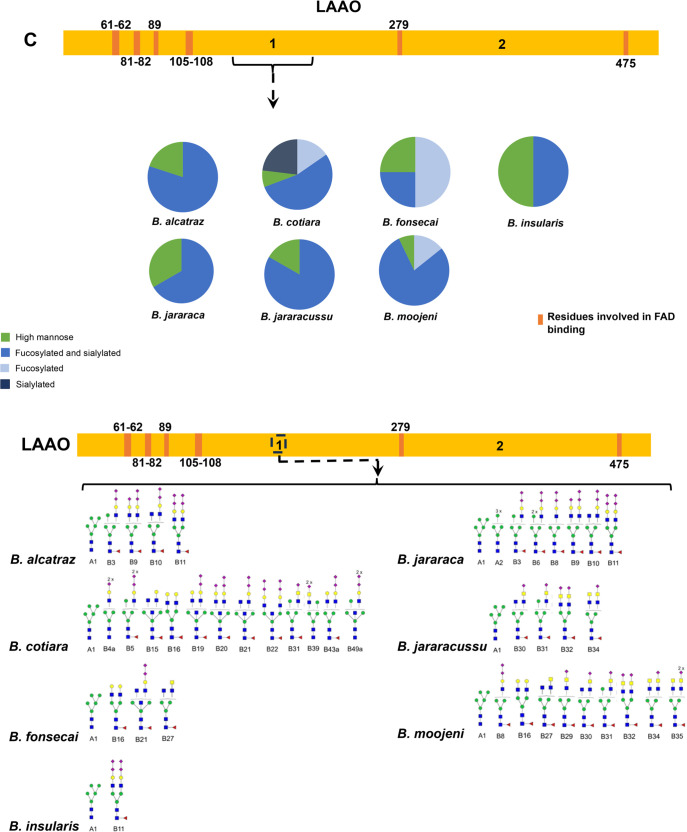

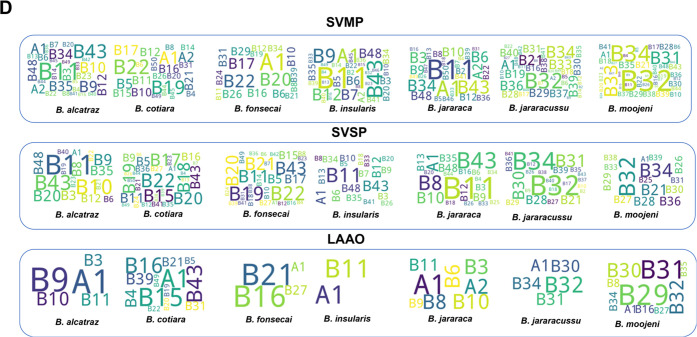
*N-*glycosylation variability in *Bothrops* toxins. (A) Scheme of a typical P–III SVMP precursor showing the distribution of the *N*-glycans features identified in this study. *N*-glycosylation sites identified in this study are numbered. Description of the *N*-glycan compositions identified in the *N*-glycosylation site #1 in the pro-domain, #8 in the catalytic domain, and #13 in the cysteine rich domain. (B) Scheme of a SVSP precursor showing the distribution of the *N*-glycan features identified in this study. Description of the *N*-glycan compositions identified in the *N*-glycosylation site #2 of a SVSP. (C) Scheme of a LAAO precursor showing the distribution of the *N*-glycans features identified in this study. Description of the *N*-glycan compositions identified in the *N*-glycosylation site #1 of a LAAO. (D) Word clouds of the *N*-glycan composition IDs described by Andrade-Silva et al.^15^ identified in this study in SVMPs, SVSPs, and LAAOs. Word clouds were generated by using https://chartexpo.com/wordcloud.

The *N*-glycosylation site located in the pro-domain was occupied by hybrid/complex compositions containing sialic acid and fucose residues in all venoms. The sites in the catalytic domain were also more often found with fucosylated and sialylated hybrid/complex compositions; however, at lower frequency, high-mannose *N*-glycans were also identified. The occupation of the *N*-glycosylation sites in the Cys-rich domain was remarkably variable between the venoms and in the case of *B. moojeni* venom, only fucosylated and sialylated glycans were identified. As the occurrence of *N*-glycosylation sites is a variability factor in SVMPs, an analysis of the glycan occupation of one illustrative site in the pro, catalytic, and cysteine-rich domains is shown describing the compositions identified in the different venoms (Figure 6A).

Even though it is not possible to speculate about the abundance of *N*-glycoforms identified in SVMPs, clearly there is significant diversity in most *N*-glycosylation sites of this toxin class. Considering the differences between the high-mannose and hybrid/complex *N*-glycan compositions, in terms of charge and size, it is possible to speculate that there could be structural differences between the proteoforms containing these two types of *N*-glycans. Although it is not possible to consider these observations as a case of microheterogeneity, as it is unknown whether all identified *N*-glycoforms belong to the same protein, it is possible to conceive that these two types of *N*-glycan chains could be present in the same structural region of different SVMPs, implying that the different *N*-glycans could possibly regulate differential folding of nascent proteins or modulate their interactions with substrates.

The variability of one of the identified *N*-glycosylation sites belonging to the SVSPs is represented in Figure 6B. Notably, the occupation of this *N*-glycosylation site is extremely variable among the venoms. The glycosylation site depicted in Figure 6B is in most venoms occupied by hybrid or complex *N*-glycan compositions.

In the case of the LAAOs, despite their relative low abundance in *Bothrops* venoms, one *N*-glycosylation site at Asn 172 is described as conserved.^63^ We identified *N*-glycosylated peptides covering the Asn172 residue in all venoms (Figure 6C). A second *N*-glycosylation site (Asn 361) has also been identified in some LAAOs; however, its presence was not detected in this study. The Asn172 was identified as containing sialylated, nonsialylated, and high-mannose *N*-glycan compositions in the different venoms. Interestingly, the level of variability was distinct between the venoms, with *B. cotiara* and *B. moojeni* showing a higher number of different *N*-glycan compositions occupying Asn172.

Figure 6D shows word clouds representing the *N*-glycan compositions identified in SVMPs, SVSPs, and LAAOs. This type of display allows visualization of the frequency of identifications of the different compositions in these three toxin classes, providing insights into the most common *N*-glycans occupying sites in venom enzymes important in the pathophysiology of envenomation.

Considering the venom profiles of *N*-glycoproteome heterogeneity described here, we used the arc diagram plots to evaluate the correlation among the *N*-glycan compositions.^59^ To this end, the number of co-occurrences of different compositions at the same site, regardless of the toxin class, was considered, and the pairs of *N*-glycans found at least three times in the same site were selected (Figure 7). Considering the two high mannose compositions found in all venoms, composition A1 (Man5) is more frequent than composition A2 (Man6). This analysis corroborates the previous data from GNA lectin blot experiments, indicating that the high mannose *N*-glycan compositions are conserved among these species.

**Figure 7:**
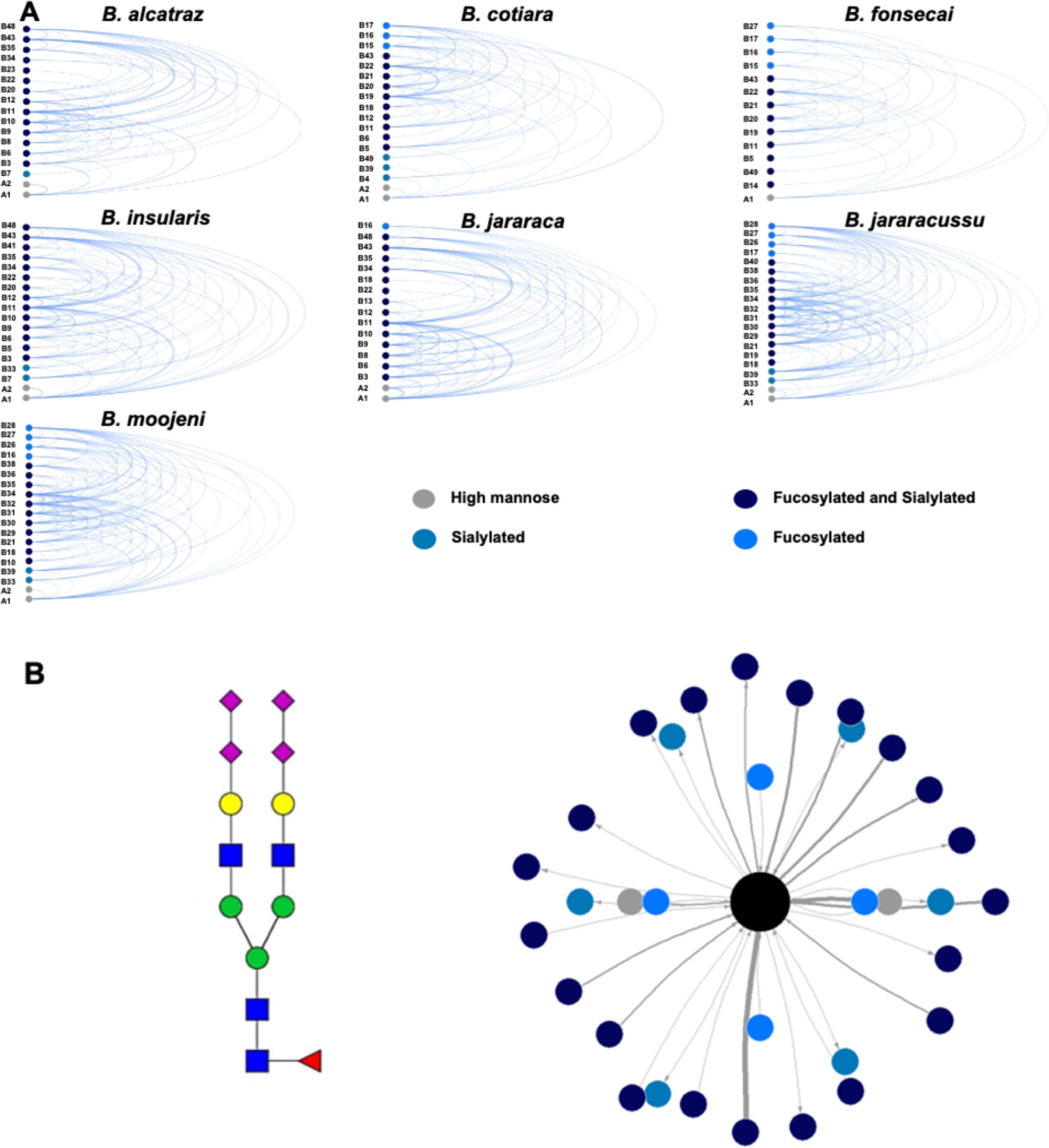
Network analysis of the co-occurrence of identified *N*-glycans at *N*-glycosylation sites. (A) Arc diagram plots of pairs of *N*-glycan compositions found at least three times at the same site. The nodes represent the *N*-glycan compositions, and the width of the edges is proportional to the co-occurrences of these compositions. (B) Glycan co-occurrence network centered on the *N*-glycan composition (Hex)­2 (HexNAc)­2 (Deoxyhexose)­1 (NeuAc)­4 + (Man)­3­(GlcNAc)­2 identified in *B. jararaca* *N*-glycopeptides. At the left side is the proposed structure of this composition, and on the right side is the network graph. Edge colors distinguish *N*-glycan classes and edge thickness the weight of occurrence counting.

As observed in the NeuAc counting analysis (Figure 4), we identified a prominent occurrence of sialylated compositions in *Bothrops* venoms. Arc diagrams of the *N*-glycan compositions are depicted in Figure 7A. A comparison of the nodes and the distribution of the edges shows that it is possible to classify the venoms according to resemblance in three different groups. The first is composed of *B. alcatraz*, *B. insularis*, and *B. jararaca* venoms, which share the identity of the nodes, the most prominent pair [B43; (Hex)_2_(HexNAc)_2_(Deoxyhexose)_1_(NeuAc)_2_+ (Man)_3_(GlcNAc)_2_ and B11; (Hex)_2_(HexNAc)_2_(Deoxyhexose)_1_(NeuAc)_4_+ (Man)­3­(GlcNAc)_2_] and a similar distribution of the edges. *B. cotiara* and *B. fonsecai* venoms compose the second group, as they share the identity of the nodes and the general distribution of edges. Moreover, the group of *B. jararacussu* and *B. moojeni*venoms shares the node identity and shows the most complex network, with a higher crossover between the compositions of the hybrid/complex class. Even though the toxin class in which these sites were identified was not taken into account in this kind of analysis, the results illustrate the general use of *N*-glycosylation sites in the venoms. Figure 7B graphically summarizes the complexity of heterogeneity of glycoproteomics results showing the glycan co-occurrence networks of one of the most abundant *N*-glycan compositions found in *B. jararaca* venom glycoproteins.

The identified *N*-glycopeptides were used to classify the venoms by hierarchical clustering of their unique *N*-glycopeptides in a binary matrix assignment, considering the complete linkage and the Euclidean distance. Figure 8A shows the graphical visualization of the two hierarchical clusterings of the venom *N*-glycoproteome characterization, which considers, for each pair (venom, *N*-glycopeptide) the presence or absence of a given unique *N*-glycopeptide. According to this analysis, similarly as it was obtained in the characterization of *Bothrops* whole venom proteomes, glycoproteomes, and *N*-glycomes,^14,15,28^ the venoms clustered similarly to their phylogenetic species classification.^40^ *B. jararacussu* and *B. moojeni* clustered together, although they showed the most distinct *N*-glycoproteome composition, while *B. cotiara* and *B. fonsecai* formed a second group, and the venoms of the Jararaca complex (*B. alcatraz*, *B. insularis*, and *B. jararaca*) clustered together. Considering the challenges involved in intact *N*-glycopeptide analysis and the limitations related to the database and fragmentation efficiency for both peptide and *N*-glycan moieties, we decided to evaluate the MS1 profile of the mass spectrometry data (neutral masses) as an alternative way to compare the venoms using the clustering analysis. The neutral mass values correspond to the precursor mass of the deisotoped peaks without the adduct mass. This independent clustering that uses the neutral masses of the identified peaks (Figure 8B) showed a cluster organization similar to that described in Figure 8A. In both cases (*N*-glycopeptide and neutral mass), the clustering analysis resulted in profiles similar to the *Bothrops* species phylogenetic classification.^40^

**Figure 8:**
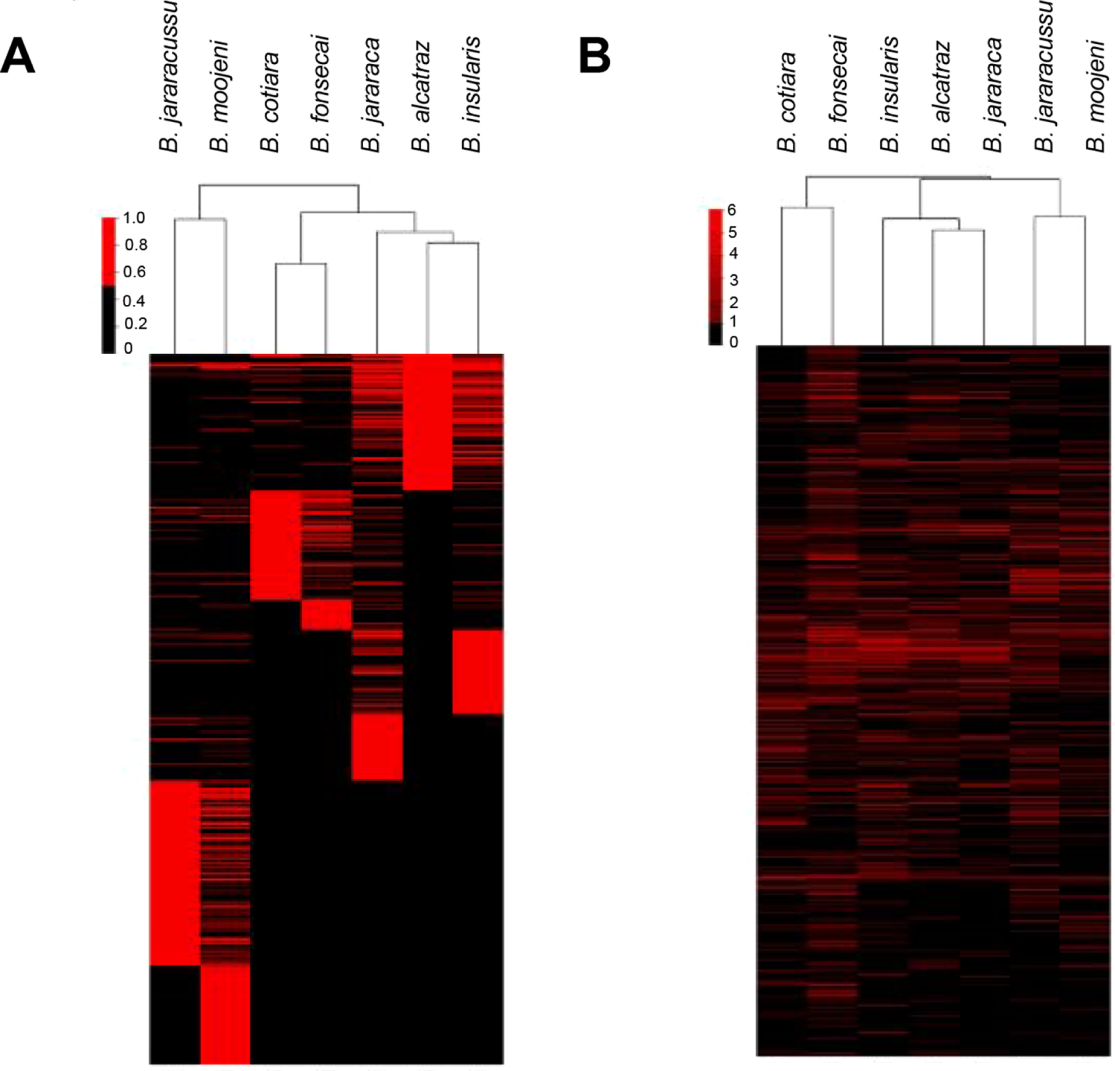
*Bothrops* venom clustering according to glycoproteomes. Graphical visualization of the two hierarchical clustering of identified intact *N*-glycopeptides by a combination of peptide sequence + *N*-glycan composition (A) and by their neutral masses (B). For each venom, a given *N*-glycopeptide or neutral mass is either present (red) or absent (black).

### 3.7 Analysis of Nonglycosylated Peptide Fractions

We also analyzed the peptides that did not bind to TiO_2_ beads or to the HILIC resin. Although only a fraction of the venom proteins was submitted to trypsin digestion and LC-MS/MS, this analysis resembles a typical bottom-up proteomic characterization of a snake venom. Extracted ion chromatograms and the distribution of peptide *m*/*z* values of LC-MS/MS analysis of nonglycosylated fractions are depicted in Figure S23, while the counting of missed trypsin cleavages is shown in Figure S24. The identifications of the peptides in the nonbound fractions are described in Tables 27–33 (information from the MaxQuant evidence file) and Tables 34–40 (information from the MaxQuant protein groups file). The analysis of the nonglycosylated peptide fractions showed that the three technical replicates shared ∼50% of the identified peptides (Figure S25), with high counting of peptides identified in at least two replicates of LC-MS/MS (Figure S26). As a result of these analyses, Figure 9 shows the number of protein groups identified in each venom and their distribution among the main toxin classes found in *Bothrops* venoms.

**Figure 9:**
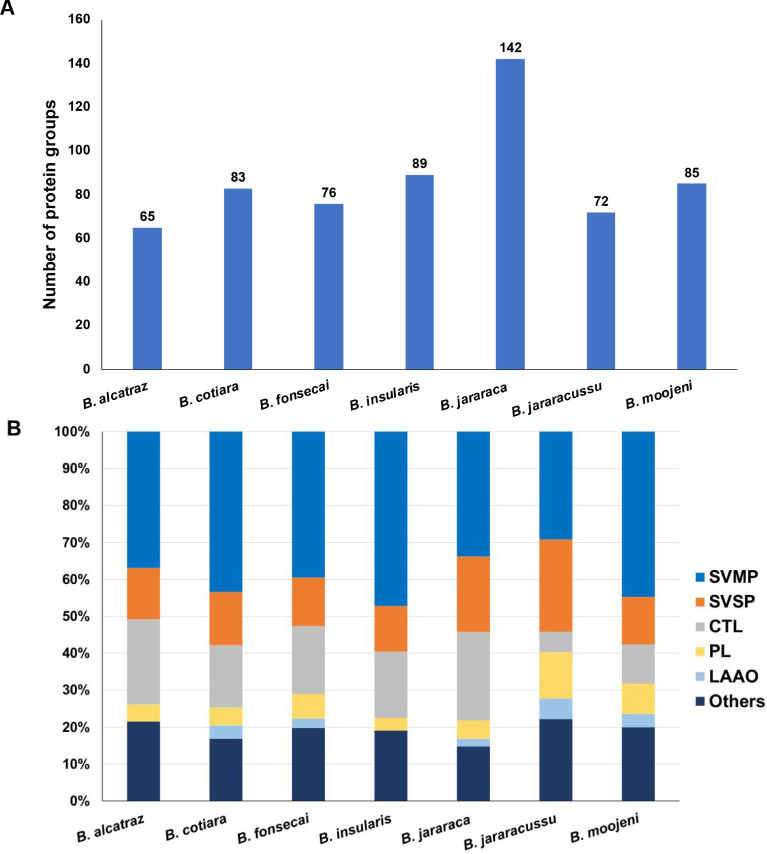
Analysis of peptides identified in the fractions that did not bind to TiO_2_ beads and to the HILIC resin. (A) Bar graph of the comparison of the number of protein groups identified in the nonglycosylated peptide fractions. (B) Toxin class distribution of the protein groups identified in the nonglycosylated peptide fractions.

In this analysis, the highest number of protein groups was identified in the venom of *B. jararaca*, while the lowest number was identified in the venom of *B. alcatraz* (Figure 9A). Considering that the numbers of identified protein groups represent only protein diversity and not abundance of these venom components, the identification of a higher number in *B. jararaca* venom may be a consequence of the high amount of information about toxin structures of this venom present in the protein databases. Although a very low number of protein sequences from *B. cotiara* and *B. fonsecai* venoms are known, the protein diversity identified in these venoms was identified as similar to the other species.

The distribution of proteins regarding the main toxin classes showed the typical diversity of *Bothrops* venom proteomes and glycoproteomes (Figure 9B).^14,64^ In all venoms, SVMPs and SVSPs together corresponded to about 50% of the protein groups, while the minor components (others) corresponded to nearly 20%. Compared to the other venoms, *B. jararaca* and *B. jararacussu* showed a relatively higher proportion of proteins belonging to the SVSP class, along with a relatively lower proportion of SVMPs. Another interesting difference was the diverse profiles of CTL identifications, which showed, comparatively, a lower number of *B. jararacussu* and *B. moojeni* venoms. On the other hand, the PL class was represented by a higher number of protein groups in these two venoms.

### 3.8 Comparison of Identifications of Intact *N*-Glycosylated and Nonglycosylated Peptides

The results of the complex analysis of *Bothrops* venom composition described here also allowed for a comparison of the proteomes obtained by the identification of intact N-glycosylated and nonglycosylated peptides. For this purpose, we generated Upset plots^65^ to show the intersections between identified peptide backbones (Figure 10).

**Figure 10:**
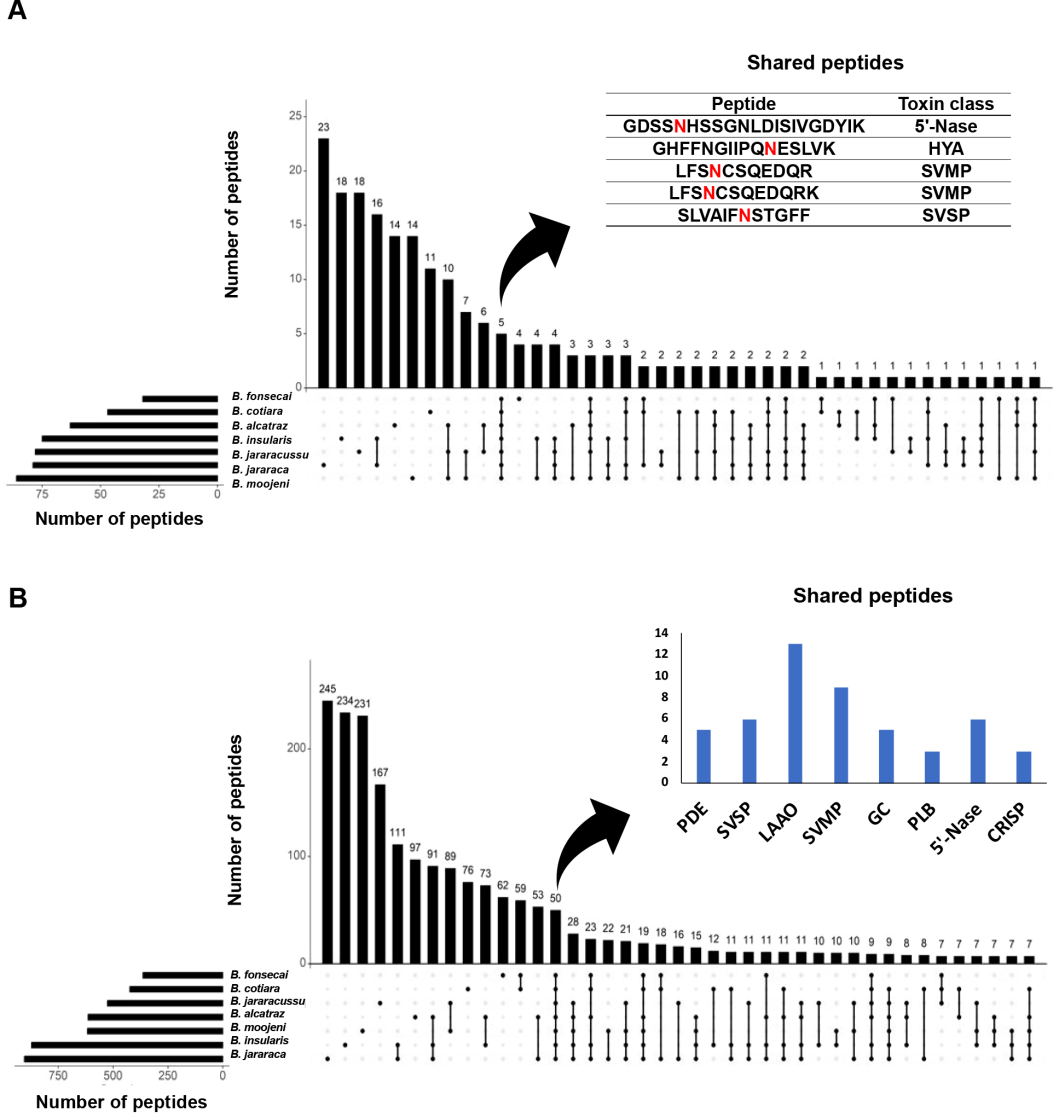
Comparison of peptide backbones identified in the intact *N*-glycosylated and nonglycosylated fractions of *Bothrops* venoms. (A) Upset plot of peptide sequences identified in the intact *N*-glycosylated fractions. The asparagine involved in *N*-glycosylation sites are labeled in red.(B) Upset plot of peptide sequences identified in the nonglycosylated fractions. *Y*-axis: number of peptides (unique or shared). Horizontal bars represent the total number of identified sequences in each venom. Connections between the circles represent peptides shared by the venoms. Unconnected circles represent unique peptides.

The analysis of the intact *N*-glycosylated fractions (Figure 10A) showed that most peptide backbones were exclusively identified in different venoms, while only five were shared by all venoms. *B. jararaca* and *B. insularis* venoms showed the highest number of exclusive peptide sequences, respectively, 23 and 18, whereas in the other venoms around 30 exclusive peptide sequences were identified. Interestingly, peptides common to all venoms were restricted to four toxin classes: 5′-Nase, HYA, SVMP, and SVSP. Concerning the peptide identified in the nonglycosylated fractions (Figure 10B), most were exclusively identified in only one venom, and all species shared only 50 sequences. The species with the highest number of exclusive peptides were *B. jararaca* and *B. insularis*, respectively, 245 and 234. Differently from the analysis of common peptide sequences of the intact *N*-glycosylated fractions, the 50 common peptides identified in the nonglycosylated fractions were derived from various toxin classes. Overall, both intersection profiles revealed a significant level of diversity between the venoms in both peptide backbone sets, adding to the notion that venom proteome variability is impacted by both amino acid sequence variations and occupation of the *N*-glycosylation sites.

The amino acid backbones of glycosylated peptides and nonglycosylated peptides were independently used to classify the venoms by hierarchical clustering (Figure S27) interestingly resulting in *Bothrops* venoms clustering according to their phylogenetic classification.^40^

## 4 Discussion

This study builds upon our previous *N*-glycomic analysis of *Bothrops* venoms that revealed *N*-glycans characterized by different degrees of branching, isomer structures, and variable abundances, contributing to the complexity of venom proteomes, and the suggestion that the subproteomes of glycoproteins reflect the phenotypic plasticity found in congeneric species indicating distinct molecular signatures for each venom.^15^ Here, *N*-glycosylation in proteins of seven *Bothrops* venoms was deeply explored by (i) the quantification of neutral sugars in the venoms, (ii) the evaluation of the presence of carbohydrate structures by blotting using five lectins of different specificities, and (iii) the application of high-throughput mass spectrometric approaches to identify *N*-glycosylation sites and their corresponding *N*-glycan compositions in tryptic peptides, resulting in the most extensive profiling of snake venom glycosylation so far.

We started this study by the evaluation of the molecular masses of venom proteins by gel electrophoresis, under reducing and nonreducing conditions, which revealed protein band differences likely due to variable glycosylation levels (Figure S1). Indeed, it has been shown that, after de-*N-*glycosylation, the protein profiles of *Bothrops* venoms are more similar than in their native forms.^14,66^ Thus, the differences observed in the evaluation of venom protein molecular mass by gel electrophoresis in this and other studies may reflect distinct *N*-glycan compositions or differences in the presence/occupation of *N*-glycosylation sites.^10,14,26^ On the other hand, some observed differences in the low molecular mass range, especially below 20 kDa, could be explained by the differential abundance of some low molecular mass venom components, including toxin classes that are not glycosylated, such as CTL and PLA2.

The evaluation of venom glycosylation levels by measuring the contents of neutral carbohydrates did not reveal significant differences between the *Bothrops* venoms, and the value obtained for *B. jararaca* venom agreed with a previous analysis of this venom by the same method (Figure S2).^67^ Although the method used does not allow for the quantification of all types of monosaccharides commonly found in proteins, the data can be used to estimate the overall venom protein glycosylation level. From the *N*-glycosylation perspective, the basic core contains mannose units that can be detected by the method used. In the case of *O*-glycosylation, galactose units are frequently present in *O*-glycans, which also ensures some detection. A noteworthy comparison that confirms that the quantification of neutral carbohydrates may be used as an estimate of protein glycosylation levels involves the venoms of *Crotalus durissus* *sp.*,^68^ whose values are around three times lower than those found for *B. jararaca* venom^67^ and agree with the fact that, in comparison to *Bothrops*, the venoms of *C. durissus* sp. contain a very low content of glycosylated proteinases (SVMPs and SVSPs).

Further, the qualitative analysis by lectin blot allowed the comparison of the occurrence of some carbohydrate structures in the venoms, and the results agreed with our previous *N*-glycomic data.^15^ In the case of GNA lectin, which binds to mannose units, it was interesting to observe the similar pattern of recognition among the venoms in agreement with our identification of *N*-glycosylated peptides, most of which showed the presence of shared *N*-glycan compositions of the high-mannose type in eight toxin classes (SVMP, SVSP, LAAO, 5′-Nase, APase, PDE, PLB, and HYA) identified in all venoms (Figure S6). In contrast, the lectin PNA did not recognize glycoproteins in any *Bothrops* venom (Figure S7). However, this result is limited by the fact that sialic acids inhibit PNA binding, and this condition was not tested. Even though *B. jararacussu* and *B. moojeni* venoms showed a weakly stained band of approximately 13–14 kDa that cross-reacted with the lectin, it likely does not contain a glycoprotein, but could rather contain a PLA2, since it has been shown that this kind of toxin, although not glycosylated, may interact with lectins.^69,70^ There are only a few members of the venom PLA2 class that are glycoproteins,^27,71^ and none of them were found in *Bothrops* venoms. Interestingly, mammalian secreted PLA2s are recognized by receptors that contain C-type lectin domains.^72^

The lectins MAA and SNA (Figures S3 and S4) revealed significant differences in the recognition of proteins, which allowed the classification of the venoms in two groups: #1 (*B. alcatraz*, *B. cotiara*, *B. fonsecai*, *B. insularis*, and *B. jararaca*) and #2 (*B. jararacussu* and *B. moojeni*). MAA is generally used to probe whether the linkage between the sialic acid and galactose units at the nonreducing end of glycan structures is α-2,3. Our previous *Bothrops* *N*-glycome analysis indicated that the sialic acid unit in the *N*-glycan antennae is linked by a α-2,3 linkage.^15^ On the other hand, the lectin SNA, which recognizes sialic acid linked to galactose residues by an α-2,6 linkage, showed no recognition of proteins in all venoms, a finding that would suggest the absence of this type of linkage in *Bothrops* venom glycans. Since the proteins of *B. jararacussu* and *B. moojeni* venoms were not recognized by both MAA and SNA lectins, probably because of the absence of a Gal unit in their N-glycan antennae, it was not possible to define the linkage in the glycans of these venoms in this study (Figures S3 and S4).

In the case of the DSA lectin, which recognizes the structure Gal-1,4-GlcNAc and the polysaccharide chitin,^73^ the venoms showed a profile of weakly stained bands, except for *B. jararacussu* and *B. moojeni* venoms that were strongly recognized (Figure S5). The unexpected weak recognition observed in the venoms of *B. alcatraz*, *B. cotiara*, *B. fonsecai*, *B. insularis*, and *B. jararaca* could be explained by the presence of a sialic acid dimer in the Gal-1,4-GlcNAc structure, which could impair the recognition of *N*-glycan chains by DSA. Regarding the venoms of *B. jararacussu* and *B. moojeni*, the antenna composition of all *N*-glycans previously identified was HexNAc-1,4-HexNAc.^15^ Even though the data did not allow the determination of the *N*-acetylhexosamine that composed the *N*-glycans, their identities were suggested based on an analysis of the *N*-glycans found in an SVSP of *B. moojeni* venom.^74^ In this way, the strong recognition of glycoproteins by DSA in these two venoms may indicate that some *N*-acetylhexosamine dimers could be formed by GlcNAc–GlcNAc instead of the predicted GalNAc-1,4-GlcNAc structure.

In the analysis of *N*-glycosylated peptides enriched in the intact glycopeptide fractions, the large set of precursor ions generated in the mass spectrometer was originated from various types of peptides. As we used TiO2 affinity chromatography to enrich glycopeptides, any other peptide with affinity might have been enriched and composed of our sample subjected to LC-MS/MS, and possibly, eventually, also phosphopeptides. Moreover, precursors of *O*-glycosylated peptides were certainly present in our glycopeptide-enriched samples, and these were not assigned since our database is composed of *N*-glycans. We indeed have attempted to explore *O*-glycans in snake venom toxins, but so far, no reliable results have been obtained. Nonetheless, considering that about half of the putative *N*-glycosylated peptides were identified as glycosylated in the intact glycopeptide analysis using our designated set of *N*-glycans, we acknowledge that as a reasonable identification rate, which ensued in the identification of 1,715 intact *N*-glycosylated peptides present in all putatively glycosylated toxin classes of seven venoms.

Concerning the features of the glycosylation process, variability originates from the number of occupied glycosylation sites and the glycan structures attached to them.^75^ In *Bothrops* venoms, the contribution of *N*-glycosylation in interspecies variability was evidenced by the different numbers of intact *N*-glycopeptides identified. Although the analysis of peptide backbones revealed a higher number in *B. jararaca* venom, *B. jararacussu* and *B. moojeni* venoms (Figure 3) showed the highest number of identified intact *N*-glycopeptides whereas *B. fonsecai* venom showed the lowest. Nevertheless, the numeric differences cannot be interpreted as differential glycosylation levels in the venom proteins and might rather be due to (i) differences in the number of proteins available in the protein database employed in the search and (ii) the lack of *N*-glycomic data of *B. fonsecai* venom. Further, variation in the *N*-glycan compositions may also have contributed to the differences in the identification of intact *N*-glycosylated peptides, as some compositions could have been favored or disfavored in the ionization and fragmentation processes of mass spectrometry.

In all venoms analyzed in this study, high-mannose and hybrid/complex-type *N*-glycans were identified in all toxin classes. Our analyses also revealed that, in general, there was a predominance of hybrid/complex type *N*-glycans, and most of them were sialylated and fucosylated. However, the number of sialic acid units per *N*-glycan composition showed remarkable differences between the venoms and toxin classes, especially in proteinases (Figures 4 and Figure S18).

In our study, the heterogeneity analysis revealed that, considering all toxin classes together, the venoms showed similar average of heterogeneity: approximately three *N*-glycoforms per *N*-glycosylation site (Table S26). However, considering each venom separately, it was evident that heterogeneity varied between the toxin classes, and despite the higher number of different polypeptide chains of SVMPs and SVMPs, some nonabundant components, such as LAAO, HYA, and DPase, showed the highest heterogeneity levels (Table S26).

Overall, our data suggest that in *Bothrops* venom, protein variability involves both peptide backbones and *N*-glycan chains. Even in the case of venoms with similar *N*-glycan repertoires, such as *B. alcatraz*, *B. insularis*, and *B. jararaca*, our results did not show complete overlap of *N*-glycan compositions and *N*-glycosylated peptides (Figures S14 and S15). Furthermore, the identified *N*-glycosylated peptide backbones also represented a variability factor, as some peptide sequences were not identified in closely related species (Figure S27).

The evolutionary history of snake venom toxins and their hypothetical ancestors reveals progressive alterations in the amino acid composition and structural characteristics with the generation of functionally diverse molecules. Based on our analyses, despite the notable variability of occupation of glycosylation sites in *Bothrops* venom proteins, we show that *Bothrops* venom glycoproteomes contain a core of components that define their framework, which is conserved upon evolution in parallel to other molecular markers that determine their phylogenetic classification, as viewed by the clustering of the seven species, according to glycopeptide backbone + glycan composition, or glycan composition, or peptide backbone (Figures 7 and 8 and S27). Therefore, paradoxically, *Bothrops* venom phenotypes are conserved and divergent, indicating that evolution and divergence in the venom composition among species might be related to evolutionary responses to diet, habitat, and predator–prey interactions and need further studies to be fully understood.

Another noteworthy aspect of *Bothrops* venom protein glycosylation is the fact that it is a PTM restricted to polypeptide chains that contain at least 230 amino acid residues, such as the SVSPs, P–II and P–III SVMPs, and some less abundant enzymes (LAAO, HYA, 5′-Nase among others). Polypeptide chains containing around 200 amino acids or less, such as P–I SVMP, CRISP, PLA2, and CTL, are either devoid of or contain only one putative *N*-glycosylation site (for example, a few P–I SVSPs). Besides the importance of *N*-glycosylation in proteins related to their biological functions, stability, solubility (especially for secreted proteins), and protection from proteolysis, this PTM is also crucial in the quality control of protein folding taking place in the endoplasmic reticulum, shaping the glycoproteome of eukaryotic cells.^76,77^ *N*-glycan positioning and composition (glyco-code) determines lectin chaperone selection; thus, *N*-glycosylation is important for the fate of newly synthesized secretory proteins, such as the venom proteins in the venom gland, where a productive fold may take place, resulting in proteins successfully secreted, or incorrectly folded proteins may be routed for displacement from the endoplasmic reticulum and be headed for degradation. It is therefore intriguing the fact that abundant toxins, such as CTL and PLA2, are not putatively *N*-glycosylated, however, display refined molecular structures that are fundamental for their functional activities.

Snake venom intra- and interspecies variability has been explored from different perspectives.^9,11,14,64,78–80^ The hypotheses behind this phenomenon usually consider two main possibilities: the presence of specific toxins and the differential abundance of specific toxin classes in the venoms. Although *N*-glycosylation is a common PTM in snake toxins, the identification of glycosylation sites, the features of glycan chains attached to toxins, and their role in shaping venom phenotypes are poorly explored. Interestingly, our data revealed some insights into the contribution of *N*-glycosylation to proteoform generation in some toxin classes.

Based on the *N*-glycosylation sites identified in this study, all *N*-glycosylated toxin classes in *Bothrops* venoms are enzymes that were identified as mainly containing *N*-glycan compositions of the hybrid/complex type, most of which were sialylated and fucosylated. Considering the human plasma as a reference,^81^ as it is, similarly to the venom, composed of secreted proteins, a high sialylation level in proteins is a common feature between them, which could be related to the fact that most toxins have systemic targets and should be carried by the circulation system to act in distant organs in the prey organism. In plasma glycoproteins, the presence of noncaped Gal, GalNAc, and NeuAc-2,6-GalNAc at the nonreducing end of glycan chains is related to their uptake by the asialoglycoprotein receptor (ASGPR) by parenchymal liver cells.^82^ These receptors are also present in rodents,^83^ which are natural prey of many snake species, and therefore, it is possible that snake toxins could be selected upon evolution to contain sialic acid units to increase their half-life and favor their systemic effects. Interestingly, human plasma and venom *N*-glycosylated proteins differ in that the latter, according to our results, seem to contain more core fucosylated *N*-glycans.^81,84^ Core fucosylation is an important modification of the *N*-glycan core in eukaryotes,^85^ and our venom *N*-glycomic analysis suggested the presence of this modification in most *N*-glycan compositions of *Bothrops* venoms. Core fucosylation modifies the *N*-glycan conformation, supporting a different orientation of the α1,6-arm.^86^

Another important point of analysis is whether venom protein *N*-glycosylation is toxin class-specific or whether different *N*-glycan chains might be shared by different toxin classes. Our data did not show any specific *N*-glycan distribution among the toxin classes, and apparently, in each venom, *N*-glycan compositions of high-mannose and hybrid/complex types are found in all *N*-glycosylated proteins.

Considering the venom production cycle, it has been recently described that in the venom gland, some cells are committed with the synthesis of some toxin families, instead of a general production of the venom components distributed among all gland cells.^87^ This finding can be also correlated to another study that revealed an asynchronous synthesis of different toxin classes during the venom production cycle.^88^ Concerning the venom glycoproteins, the *N*-glycosylation process involves nontemplate-driven pathways, and the factors that affect variability in the *N*-glycosylation sites are steric accessibility of sequons during the protein translation and traffic along the endoplasmic reticulum and the Golgi lumen, and availability of nucleotide sugar in these organelles.^89,90^ Further, as the *N*-glycosylation process is primarily cell-type-specific,^75^ sharing of *N-*glycan structures by the venom glycoproteins is a more parsimonious process, resulting in an unspecific use of *N*-glycan chains in the different toxin classes.

Venoms of the *Bothrops* genus have been prospected by proteomic approaches,^10,43,91,92^ and a previous study of seven species found a similar profile of their glycoproteomic phenotypes and phylogenetic classification.^14^ Regarding snake venom proteome complexity, considering the possible contribution of mRNA splicing as a factor for protein variability,^93^ the protein inference problem in proteomic analyses performed by the bottom-up approach,^94^ and the PTMs, particularly glycosylation, it is still not possible to accurately assess the number of proteins and proteoforms that compose viperid venom.

In general, proteomic studies of the venoms of the *Bothrops* genus, including this work, were performed using bottom-up approaches. Hence, here, the numbers of protein groups identified by the analysis of the peptide nonglycosylated fractions agree with those usually reported in other studies (Figure 9).^14,95^ The differences in the number of protein groups identified in the different venoms could be explained by the database used in the spectra search, which does not contain similar contents of protein sequences of the analyzed species. Nevertheless, as expected, most toxins that were detected in all of the venoms were SVMPs and SVSPs.

A comparison of backbones identified in the intact *N*-glycopeptide and nonglycosylated peptide fractions showed quite different profiles. The five common *N*-glycosylated peptides identified in all venoms belong to abundant toxins (SVMP and SVSP), while low-abundant components (5′-Nase and HYA) (Figure 10A) also showed a few conserved glycosylated sequences. On the other hand, the 50 conserved peptides identified in the nonglycosylated fractions mapped to eight toxin classes (Figure 10B). In general, MS-driven data of snake venom proteomes are analyzed in terms of the comparison of unique and shared toxins, as shown in Figure 9B. The comparison of nonglycosylated tryptic peptides shown in Figure 10B is an alternative way to evaluate proteome diversity, which, in the case of the venoms analyzed in this study, revealed conserved and variable peptides of various toxin classes and offered a different view of intragenus variability that took into account amino acid sequences and might overcome the bias of comparing venom proteomes rich in proteoforms.

The efficiency of the utilization of *N*-glycosylation sites depends on the kinetics of the transfer of a lipid-linked oligosaccharide to asparagine residues in sequons of nascent polypeptide chains.^19,96^ A study on the characterization of the proteome of the venom glands of *B. jararaca*, taking into account two distinct phases of its ontogenetic development and the marked sexual dimorphism of this species, demonstrated variable abundances of proteins associated with glycosylation processes and folding, in comparisons of newborn × adult and female × male venoms, suggesting that the variation in the expression levels of cellular proteins might give rise to an even higher variation in the expressed toxins.^97^ Although no mechanism to determine or impact *N*-glycosylation site occupancy in snake venom proteins has been demonstrated, the low number of common *N-*glycosylated peptides identified here suggests that the variability in glycosylation is an important feature of proteomes.

One of the most significant findings of this study was the confirmation of the high sialylation levels of toxins in *Bothrops* venoms, and the presence of this monosaccharide could be related to a higher half-life time in the prey body.^82^ However, sialylation could be important to the toxin function as well, as suggested by studies that explored the importance of sialylated glycan structures in the biological activities of LAAO, SVMP, and SVSP.^46,98–100^ In the case of an LAAO of *Calloselasma rhodostoma* venom, the *N*-glycosylation was not necessary for its catalytic activity but was essential for induction of apoptosis.^99^ The activities of the sialylated and nonsialylated forms of the SVMP bilitoxin-1 were evaluated by Nikai et al.,^98^ who showed that sialic acid was not important for substrate hydrolysis, but its removal promoted a decrease of hemorrhagic activity. In the SVSP acutobin, the presence of a sialic acid dimer structure showed to be important for enzymatic activity and protein stability.^100^ Furthermore, in a comprehensive study, carbohydrate chains containing sialic acid were shown to play a role in the activity of proteinases of *Bothrops* venoms upon macromolecular substrates.^46^ Besides the importance of *N*-glycans containing sialic acid in the enzymatic activity of toxins, there is also the possibility of sialylated glycan chains to interact with proteins of the Siglec family (sialic acid-binding immunoglobulin-type lectins).^101^ These lectins are widely expressed by immune cells, and their engagement by sialylated toxins could play a role in the envenomation process. The occurrence of sialic acid dimers in snake toxins was first identified by Lin et al.^58^ and, in our study, it was more often found in toxins of *B. alcatraz*, *B. cotiara*, *B. fonsecai*, *B. insularis*, and *B. jararaca* venoms.

In general, the SVMP class showed an average ratio of *N*-glycosylation heterogeneity below that of the whole venoms (Figure 5and Table S26). An *N*-glycosylation site located in the C-terminal portion of the prodomain and in the vicinity of the catalytic domain was identified in the N-terminomic analysis of *B. alcatraz* ^24^ and *B. jararaca* venoms^102^ as unoccupied. In this study, we identified the same peptide backbone in the venoms of *B. alcatraz*, *B. jararaca*, *B. jararacussu*, and *B. moojeni* with its *N*-glycosylation site free and occupied (Tables S18–24 and S27–33). Farther, all *N*-glycosylated peptides located in the same region of SVMP precursors contained hybrid/complex *N-*glycans, many of them with sialic acid units, suggesting that the occupation of these *N*-glycosylation sites conveys a variability source and might affect the structure or activity of the nascent SVMPs (Figure 6A). As with other proteinases in which glycosylation sites are located in the pro-domain or in its vicinity,^103–107^ we hypothesize that the *N*-glycosylation site identified in the prodomain of SVMPs could be involved in the control of zymogen activation, and the variable occupancy of this site contributes to the N-terminal variability of the mature forms of this toxin class. In most venoms, the Cys-rich domain showed the highest level of glycosylation heterogeneity (Figures 5 and 6A). This domain of the SVMPs has been shown to play a role in their functional activities, serving as an interaction interface with substrates, and directing the toxin to specific sites.^108–110^ Thus, besides the hypervariable region of the Cys-rich domain, which is a variable spot in the structure of different SVMPs, *N*-glycosylation appears to be another feature contributing to variability as the occupation of the sites identified here was variable among the venoms (Figure 6A).

Regarding the SVSPs, it is known that glycosylation is an important variability factor, as these proteins may contain distinct numbers of putative *N*-glycosylation sites.^111^ As an example of the significance of glycosylation for some SVSPs of *Bothrops* venoms, it was shown that the glycan moiety of the heavily glycosylated *Bothrops* Protease A (BPA) is important for its substrate specificity.^112^ In another example, the SVSP PA-BJ, present in *B. jararaca* venom in at least eight proteoforms, contains an *N*-glycosylation site close to the catalytic cleft that was proposed not to affect substrate specificity but could avoid the interaction with serpins present in the blood.^60^ In agreement with the high variability of occupation of glycosylation sites by *N*-glycans containing sialic acid in *Bothrops* venoms (Figure 6B), several SVSP preparations showed distinct forms in electrophoresis, with slightly different molecular masses and pIs, due to variations in their amino acid sequences and glycosylation levels.^111^ While SVSPs show the same primary specificity as trypsin (Arg or Lys at the P1 position), they exhibit stringent macromolecular substrate specificity in contrast to trypsin, which is putatively not *N*-glycosylated and lacks specificity upon macromolecular substrates. Hence, the variable positions of putative *N*-glycosylation sites and their occupation in *Bothrops* SVSPs are likely a crucial factor conveying functional specificity to these enzymes.

The LAAOs contain variations in their tertiary structures and may show distinct physicochemical properties.^63,113^ Of special note is the high variability ratio of occupation of a conserved *N*-glycosylation site identified in LAAOs of the different *Bothrops* venoms. Even in venoms sharing the same venom *N-*glycome repertoire, such as *B. alcatraz*, *B. insularis*, and *B. jararaca*,^14^ the present study revealed that the occupation of an *N*-glycosylation site in LAAOs was heterogeneous, representing a variability spot in these toxins (Figures 5 and 6C). The carbohydrate moiety of LAAOs has been associated with the ability of the enzymes to interact with the cell surface and enhance the localization of high concentrations of H_2_O_2_;^114^ thus, the variable occupancy of *N*-glycosylation sites might be a factor in the modulation of their pathological activities as toxins in the context of the distinct venom proteomes.

As we previously reported,^14,28^ the analysis of lectin-enriched fractions of venom glycosylated proteins favors the identification of minor components, such as APase, 5′-Nase, and PDE. Even though their role in the envenomation process is poorly understood,^115^ the identification of *N*-glycosylation sites showed that the structural complexity of the venoms involves most components, with the exception of PLA_2_ and CTL. Overall, while it is not possible to predict or accurately measure the impact of the *N*-glycosylation process for the structure and function of toxins, future studies on snake venom *N*-glycosylated toxins should consider the occurrence of this PTM when comparing toxins of the same class.

## 5 Conclusions

The application of advanced approaches of mass spectrometry is fundamental for assessing PTMs in proteins of nonmodel organisms, such as snakes. This study represents the first report on the deep, high-throughput identification of snake venom *N*-glycoproteomes. The integrated application of MS-driven analysis of *N*-glycan chains and peptide backbones allowed the identification of *N*-glycosylated sites in venom proteins and their roles in intraspecies venom complexity and variability. This study also confirmed that *N*-glycan compositions belonging to the hybrid/complex class are a variability source, mainly in phylogenetically distant species. Moreover, it showed that, except for PLA2, all other venom enzymes are *N*-glycosylated, and, in general, sialylation and fucosylation are prominent features of *N*-glycans in all analyzed venoms.

Considering that the venom samples used in this study were composed of variable amounts of individual venoms, it is possible that individual variability may have contributed to an unpredictable additive effect on the compositions of glycoproteomes. Hence, ideally, an accurate comparison of these species would embody the knowledge of individual venoms. Furthermore, the extensive identification of *N*-glycopeptides reported here does not allow for the determination of the stoichiometry of all protein glycoforms in the venoms. Nevertheless, the results of this study confirmed the widely accepted notion that viperid snake venoms show a variety of modified and nonmodified toxin proteoforms.

It is not possible to unveil the function of glycosylation in a toxin without specific studies to assess the relationships between the structure and biological activity, and there is no general role that could be attributed to it. However, factors like the energy cost of venom production^116–118^ and the conservation of the presence of *N*-glycosylated toxins during the evolution process indicate that *N*-glycosylation is an important feature of the venom proteome phenotype.

Feeding habits have been associated with the variability of snake venom composition, and microheterogeneities of toxins, such as *N*-glycosylation, can be seen as functional signatures of the variability in snake venoms that could be involved in the ability of different species to deal with different types of prey. The fine-tuning mechanism of occupation of *N*-glycosylation sites can be considered as (1) parsimonious, since changes in glycan composition or in the occupation of the sequons contribute to the generation of different glycoforms, and (2) functional, since structural changes could lead to new targets in different types of prey. Nevertheless, the diversity of glycoproteins in venoms of the Viperidae family is impressive; hence, it is possible that the use of *N*-sequons and the assembly of *N*-glycan chains are basic strategies of structural diversification of toxins and not influenced by diet.

The knowledge about the location and occupation of *N*-glycosylation sites in snake venoms should help in the exploration of the role *N*-glycans could play in the function of each toxin. Further, with the recent possibility of cultivating functional organoids derived from snake venom glands,^87^ researchers have more tools to generate toxins directly from this system and create new ways to study the role of *N*-glycosylation on specific toxin sites without the bias of the heterologous production of recombinant proteins.

Therefore, paradoxically, *Bothrops* venom phenotypes are conserved and divergent, indicating that evolution and divergence in the venom composition among species might be related to evolutionary responses to diet, habitat, and predator–prey interactions, and need further studies to be fully understood.

Typically, variation in venom toxins is attributed to gene duplication and the effect of positive selection, leading to the production of functionally distinct venoms and possibly affecting the effectiveness of antivenom treatments. Our results clearly show that variable protein glycosylation is another molecular mechanism that contributes to variability and should be taken into account when investigating venom functional evolution.

Whether the variability that originated from the *N*-glycan structures is a coordinated action of glycosidases and glycosyltransferases of functional significance for the mature glycoprotein in the venom or merely an expected, stochastic generation of biologically neutral diversity is a relevant question that needs further studies to build on evidence to achieve a solid response.

The contradictory aspects of conservation of the venom phenotype framework and diversification of glycan usage, in parallel to *Bothrops* species clustering according to phylogeny, offer the opportunity for further studies to gain insights into the determinants of toxin structure and function evolution.

## Supplementary Material





## Data Availability

All mass spectrometry proteomics data have been deposited to PRIDE with the data set identifier: PXD057219.

## Notes

Experimental procedures (Pages S4–S16); venom samples; venom protein profile and carbohydrate content determination; analysis of venom protein glycosylation profiles by lectin blot; identification of glycosylation sites in venom proteins by mass spectrometry; protein quantification and digestion with Lys-C and trypsin; glycopeptide enrichment by interaction with titanium dioxide (TiO_2_); enzymatic de-*N*-glycosylation; glycopeptide enrichment by HILIC; glycopeptide fractionation using high-pH reversed-phase chromatography; LC-MS/MS analysis of de-*N*-glycosylated peptides (dF1, dF2, and dF3 fractions); LC-MS/MS analysis of intact glycopeptides (gF1, gF2.1, gF2.2, gF2.3, and gF3 fractions); LC-MS/MS analysis of nonglycosylated peptide fractions; data analysis of de-*N*-glycosylated and nonglycosylated peptide fractions; data analysis of intact *N*-glycopeptides; clustering analyses; Supplementary Figures S1–S27 (pages S16 – S45) (PDF)
Supplementary Tables S1–S40; protein groups identified in at least two replicates of the nonglycosylated peptide fraction of *Bothrops* venoms (ZIP)

## Abbreviations

5′Nase: 5′-nucleotidase
APase: aminopeptidase
CRISP: cysteine-rich secretory protein
CTL: C-type lectin
DPase: dipeptidase
DSA: *Datura stramonium* agglutinin
GC: glutaminyl cyclase
GNA: *Galanthus nivalis* agglutinin
LAAO: L-amino acid oxidase
MAA: *Maackia amurensis* agglutinin
PDE: phosphodiesterase
PLA2: phospholipase A2
PLB: phospholipase B
PNA: peanut agglutinin
SNA: *Sambucus nigra* agglutinin
SVMP: snake venom metalloproteinase
SVSP: snake venom serine proteinase

## References

[1] Barlow A., Pook C. E., Harrison R. A., Wüster W. (2009). Coevolution
of diet and prey-specific venom activity supports the role of selection
in snake venom evolution. Proc. Biol. Sci.

[2] Casewell N. R., Wüster W., Vonk F. J., Harrison R. A., Fry B. G. (2013). Complex
cocktails: the evolutionary novelty of venoms. Trends Ecol. Evol.

[3] Villar-Briones A., Aird S. D. (2018). Organic and peptidyl constituents
of snake venoms:
The picture is vastly more complex than we imagined. Toxins.

[4] Fox J. W., Serrano S. M. T. (2008). Exploring snake
venom proteomes: multifaceted analyses
for complex toxin mixtures. Proteomics.

[5] Melani R. D., Skinner O. S., Fornelli L., Domont G. B., Compton P. D., Kelleher N. L. (2016). Mapping Proteoforms and Protein Complexes From King
Cobra Venom Using Both Denaturing and Native Top-down Proteomics. Mol. Cell. Proteomics.

[6] Calvete J. J. (2018). Snake venomics
- from low-resolution toxin-pattern recognition to toxin-resolved
venom proteomes with absolute quantification. Expert Rev. Proteomics.

[7] Ghezellou P., Garikapati V., Kazemi S. M., Strupat K., Ghassempour A., Spengler B. (2019). A perspective view of top-down proteomics in snake
venom research. Rapid Commun. Mass Spectrom.

[8] Chippaux J.
P., Williams V., White J. (1991). Snake venom variability: methods
of study, results and interpretation. Toxicon.

[9] Gibbs H. L., Sanz L., Calvete J. J. (2009). Snake population venomics: proteomics-based
analyses of individual variation reveals significant gene regulation
effects on venom protein expression in Sistrurus rattlesnakes. J. Mol. Evol.

[10] Zelanis A., Tashima A. K., Rocha M. M., Furtado M. F., Camargo A. C., Ho P. L., Serrano S. M. (2010). Analysis of the
ontogenetic variation
in the venom proteome/peptidome of Bothrops jararaca reveals different
strategies to deal with prey. J. Proteome Res.

[11] Casewell N.
R., Wagstaff S. C., Wüster W., Cook D. A., Bolton F. M., King S. I., Pla D., Sanz L., Calvete J. J., Harrison R. A. (2014). Medically important
differences in snake venom composition
are dictated by distinct postgenomic mechanisms. Proc. Int. Acad. Sci.

[12] Casewell N. R., Jackson T. N. W., Laustsen A. H., Sunagar K. (2020). Causes and Consequences
of Snake Venom Variation. Trends Pharmacol.
Sci.

[13] Huang H. W., Liu B. S., Chien K. Y., Chiang L. C., Huang S. Y., Sung W. C., Wu W. G. (2015). Cobra venom proteome
and glycome
determined from individual snakes of *Naja atra* reveal
medically important dynamic range and systematic geographic variation. J. Proteomics.

[14] Andrade-Silva D., Zelanis A., Kitano E. S., Junqueira-de-Azevedo I. L. M., Reis M. S., Lopes A. S., Serrano S. M. T. (2016). Proteomic and
glycoproteomic profilings reveal that post-translational modifications
of toxins contribute to venom phenotype in snakes. J. Proteome Res.

[15] Andrade-Silva D., Ashline D., Tran T., Lopes A. S., Travaglia
Cardoso S. R., Reis M. D. S., Zelanis A., Serrano S. M. T., Reinhold V. (2018). Structures of *N*-Glycans of *Bothrops* venoms revealed as molecular signatures that contribute
to venom phenotype in viperid snakes. Mol. Cell.
Proteomics.

[16] Gagneux P., Varki A. (1999). Evolutionary considerations in relating
oligosaccharide diversity
to biological function. Glycobiology.

[17] Spiro R. G. (2002). Protein
glycosylation: nature, distribution, enzymatic formation, and disease
implications of glycopeptide bonds. Glycobiology.

[18] Wujek P., Kida E., Walus M., Wisniewski K. E., Golabek A. A. (2004). *N*-Glycosylation Is Crucial for Folding,
Trafficking, and Stability of Human Tripeptidyl-peptidase I*. J. Biol. Chem.

[19] Jones J., Krag S. S., Betenbaugh M. J. (2005). Controlling *N*-linked
glycan site occupancy. Biochim. Biophys. Acta,
Gen. Subj.

[20] Gowda D. C., Jackson C. M., Hensley P., Davidson E. A. (1994). Factor
X-activating
glycoprotein of Russell’s viper venom. Polypeptide composition
and characterization of the carbohydrate moieties. J. Biol. Chem.

[21] Nawarak J., Phutrakul S., Chen S. T. (2004). Analysis of lectin-bound glycoproteins
in snake venom from the Elapidae and Viperidae families. J. Proteome Res.

[22] Birrell G. W., Earl S. T., Wallis T. P., Masci P. P., de Jersey J., Gorman J. J., Lavin M. F. (2007). The Diversity
of Bioactive Proteins
in Australian Snake Venoms. Mol. Cell. Proteomics.

[23] Chen H. S., Chen J. M., Lin C. W., Khoo K. H., Tsai I. H. (2008). New insights
into the functions and N-glycan structures of factor X activator from
Russell’s viper venom. FEBS J.

[24] Andrade-Silva D., Zelanis A., Travaglia-Cardoso S.
R., Nishiyama M. Y. J., Serrano S. M. T. (2021). Venom Profiling of the Insular Species
Bothrops alcatraz: Characterization of Proteome, Glycoproteome, and
N-Terminome Using Terminal Amine Isotopic Labeling of Substrates. J. Proteome Res.

[25] Abu
Aisheh M., Kayili H. M., Numanoglu Cevik Y., Kanat M. A., Salih B. (2023). Composition characterization of various
viperidae snake venoms using MS-based proteomics *N*-glycoproteomics and *N-*glycomics. Toxicon.

[26] Zelanis A., Serrano S. M. T., Reinhold V. N. (2012). *N*-glycome profiling
of *Bothrops jararaca* newborn and adult venoms. J. Proteomics.

[27] Tsai I. H., Chang H. C., Chen J. M., Cheng A. C., Khoo K. H. (2012). Glycan
structures and intrageneric variations of venom acidic phospholipases
A2 from Tropidolaemus pitvipers. FEBS J.

[28] Brás-Costa C., Chaves A. F. A., Cajado-Carvalho D., da Silva Pires D., Andrade-Silva D., Serrano S. M. T. (2022). Profilings of subproteomes of lectin-binding
proteins of nine *Bothrop*s venoms reveal variability
driven by different glycan types. Biochim. Biophys.
Acta, Proteins Proteomics.

[29] Gutiérrez J. M., Calvete J. J., Habib A. G., Harrison R. A., Williams D. J., Warrell D. A. (2017). Snakebite envenoming. Nat. Rev.
Dis. Primers.

[30] da
Silva W. R. G. B., de Siqueira Santos L., Lira D., de Oliveira
Luna K. P., Fook S. M. L., Alves R. R. N. (2023). Who are the most
affected by Bothrops snakebite envenoming in Brazil? A Clinical-epidemiological
profile study among the regions of the country. PLoS Neglected Trop. Dis.

[31] Roselfeld, G. Symptomatology, pathology, and treatment of snake bites in South America. In Venomous Animals And Their Venoms Academy Press 1971, 345–384.

[32] Santoro M. L., Sano-Martins I. S., Fan H. W., Cardoso J. L. C., Theakston R. D. G., Warrell D. A., Butantan Institute Antivenom Study Group (2008). Haematological evaluation of patients
bitten by the jararaca, Bothrops jararaca, in Brazil. Toxicon.

[33] Pinho F. M. O., Yu L., Burdmann E. A. (2008). Snakebite-Induced
Acute Kidney Injury
in Latin America. Semin. Nephrol..

[34] Gutiérrez J. M., Escalante T., Rucavado A. (2009). Experimental pathophysiology of systemic
alterations induced by *Bothrops asper* snake venom. Toxicon.

[35] Sazima, I. Natural history of the jararaca pitviper, Bothrops jararaca, in southeastern Brazil. In Biology of Pitvipers. Selva 1992, 199–216.

[36] Grazziotin F. G., Monzel M., Echeverrigaray S., Bonatto S. L. (2006). Phylogeography of
the Bothrops jararaca complex (Serpentes: Viperidae): past fragmentation
and island colonization in the Brazilian Atlantic Forest. Mol. Ecol.

[37] Marques O. A. V., Martins M., Sazima I. (2002). A New Insular
Species of Pitviper
from Brazil, with Comments on Evolutionary Biology and Conservation
of the Bothrops jararaca Group (Serpentes, Viperidae). Herpetologica.

[38] Marques O. A. V., Martins M., Develey P. F., Macarrão A., Sazima I. (2012). The golden lancehead Bothrops insularis
(Serpentes:
Viperidae) relies on two seasonally plentiful bird species visiting
its island habitat. J. Nat. Hist..

[39] Martins, M. ; Marques, O. ; Sazima, I. Ecological and phylogenetic correlates of feeding habits in neotropical pitvipers of the genus Bothrops. Biology of the Vipers. Eagle Mountain Publishing 2002, 307–328.

[40] Fenwick A. M., Gutberlet R. L., Evans J. A., Parkinson C. L. (2009). Morphological
and molecular evidence for phylogeny and classification of South American
pitvipers, genera Bothrops, Bothriopsis, and Bothrocophias (Serpentes:
Viperidae). Zool. J. Linn. Soc..

[41] Alencar L. R. V., Quental T. B., Grazziotin F. G., Alfaro M. L., Martins M., Venzon M., Zaher H. (2016). Diversification
in vipers: Phylogenetic
relationships, time of divergence and shifts in speciation rates. Mol. Phylogenet. Evol.

[42] Serrano S. M. T., Shannon J., Wang D., Camargo A., Fox J. W. (2005). A multifaceted
analysis of viperid snake venoms by two-dimensional gel electrophoresis:
An approach to understanding venom proteomics. Proteomics.

[43] Gonçalves-Machado L., Pla D., Sanz L., Jorge R. J. B., Leitão-De-Araújo M., Alves M. L. M., Alvares D. J., De Miranda J., Nowatzki J., de Morais-Zani K. (2016). Combined venomics, venom
gland transcriptomics, bioactivities, and antivenomics of two *Bothrops jararaca* populations from geographic isolated regions
within the Brazilian Atlantic rainforest. J.
Proteomics.

[44] Cummings, R. D. ; Etzler, M. ; Hahn, M. G. ; Darvill, A. ; Godula, K. ; Woods, R. J. ; Mahal, L. K. Glycan-Recognizing Probes as Tools In Essentials of Glycobiology 4th Ed.; Varki, A. ; Cummings, R. D. ; Esko, J. D. ; Stanley, P. ; Hart, G. W. ; Aebi, M. ; Mohnen, D. ; Kinoshita, T. ; Packer, N. H. ; Prestegard, J. H. ; Schnaar, R. L. ; Seeberger, P. eds.; Cold Spring Harbor Laboratory Press 2022. 35536983

[45] Palmisano, G. ; Lendal, S. E. ; Larsen, M. R. Titanium Dioxide Enrichment of Sialic Acid-Containing Glycopeptides. Gel-Free Proteomics; Methods Mol Biol. 2011, 309–322. 10.1007/978-1-61779-148-2_21. 21604132

[46] Brás-Costa C., Alencar Chaves A. F., Trevisan-Silva D., Menezes M. C., Rocha M. M. T., Cajado-Carvalho D., Andrade-Silva D., Serrano S. M. T. (2023). Sialic acid-containing glycans play
a role in the activity
of snake venom proteases. Biochimie.

[47] Cox J., Mann M. (2008). MaxQuant enables high
peptide identification rates, individualized
p.p.b.-range mass accuracies and proteome-wide protein quantification. Nat. Biotechnol.

[48] Miletich J. P., Broze G. J. (1990). Beta protein C is
not glycosylated at asparagine 329.
The rate of translation may influence the frequency of usage at asparagine-X-cysteine
sites. J. Biol. Chem.

[49] Vance B. A., Wu W., Ribaudo R. K., Segal D. M., Kearse K. P. (1997). Multiple dimeric
forms of human CD69 result from differential addition of N-glycans
to typical (Asn-X-Ser/Thr) and atypical (Asn-X-cys) glycosylation
motifs. J. Biol. Chem.

[50] Sato C., Kim J. H., Abe Y., Saito K., Yokoyama S., Kohda D. (2000). Characterization of
the N-Oligosaccharides Attached to the Atypical
Asn-X-Cys Sequence of Recombinant Human Epidermal Growth Factor Receptor. J. Biochem.

[51] Chi Y. H., Koo Y. D., Dai S. Y. (2010). *N*-glycosylation
at non-canonical Asn-X-Cys sequence of an insect recombinant cathepsin
B-like counter-defense protein. Comp. Biochem.
Physiol., Part B: Biochem. Mol. Biol.

[52] Zielinska D. F., Gnad F., Wiśniewski J.
R., Mann M. (2010). Precision
Mapping of an In Vivo N-Glycoproteome Reveals Rigid Topological and
Sequence Constraints. Cell.

[53] Valliere-Douglass J.
F., Eakin C. M., Wallace A., Ketchem R. R., Wang W., Treuheit M. J., Balland A. (2010). Glutamine-linked and Non-consensus
Asparagine-linked Oligosaccharides Present in Human Recombinant Antibodies
Define Novel Protein Glycosylation Motifs. J.
Biol. Chem.

[54] Lowenthal M. S., Davis K. S., Formolo T., Kilpatrick L. E., Phinney K. W. (2016). Identification of Novel N-Glycosylation Sites at Noncanonical
Protein Consensus Motifs. J. Proteome Res.

[55] Palmisano G., Melo-Braga M. N., Engholm-Keller K., Parker B. L., Larsen M. R. (2012). Chemical
Deamidation: A Common Pitfall in Large-Scale N-Linked Glycoproteomic
Mass Spectrometry-Based Analyses. J. Proteome
Res.

[56] Carvalho P. C., Lima D. B., Leprevost F. V., Santos M. D., Fischer J. S., Aquino P. F., Moresco J. J., Yates Y., Barbosa V. C. (2016). Integrated analysis of shotgun proteomic data with
PatternLab for proteomics 4.0. Nat. Protoc.

[57] Maxwell E., Tan Y., Tan Y., Hu H., Benson G., Aizikov K., Conley S., Staples G. O., Slysz G. W., Smith R. D., Zaia J. (2012). GlycReSoft: A Software
Package for Automated Recognition of Glycans
from LC/MS Data. PLoS One.

[58] Lin C. W., Chen J. M., Wang Y. M., Wu S. W., Tsai I. H., Khoo K. H. (2011). Terminal disialylated
multiantennary complex-type N-glycans
carried on acutobin define the glycosylation characteristics of the
Deinagkistrodon acutus venom. Glycobiology.

[59] Riley N. M., Hebert A. S., Westphall M. S., Coon J. J. (2019). Capturing site-specific
heterogeneity with large-scale N-glycoproteome analysis. Nat. Commun.

[60] Yamashiro E. T., Oliveira A. K., Kitano E. S., Menezes M. C., Junqueira-de-Azevedo I. L., Paes
Leme A. F., Serrano S. M. (2014). Proteoforms of the platelet-aggregating
enzyme PA-BJ, a serine proteinase from *Bothrops jararaca* venom. Biochim. Biophys. Acta, Proteins Proteomics.

[61] Giorgianni M. W., Dowell N. L., Griffin S., Kassner V. A., Selegue J. E., Carroll S. B. (2020). The origin and diversification
of a novel protein family
in venomous snakes. Proc. Int. Acad. Sci.

[62] Wang C. R., McFarlane L. O., Pukala T. L. (2024). Exploring snake venoms beyond the
primary sequence: From proteoforms to protein-protein interactions. Toxicon.

[63] Ullah A. (2020). Structure–Function
Studies and Mechanism of Action of Snake Venom L-Amino Acid Oxidases. Front. Pharmacol.

[64] Sousa L. F., Nicolau C. A., Peixoto P. S., Bernardoni J. L., Oliveira S. S., Portes-Junior J. A., Mourão R. H., Lima-dos-Santos I., Sano-Martins I. S., Chalkidis H. M. (2013). Comparison of Phylogeny, Venom Composition
and Neutralization by
Antivenom in Diverse Species of Bothrops Complex. PLoS Neglected Trop. Dis..

[65] Lex A., Gehlenborg N., Strobelt H., Vuillemot R., Pfister H. (2014). UpSet: Visualization of Intersecting Sets. IEEE Trans. Vis. Comput. Graph..

[66] Gren E. C. K., Kitano E. S., Andrade-Silva D., Iwai L. K., Reis M. S., Menezes M. C., Serrano S. M. T. (2019). Comparative
analysis of the high
molecular mass subproteomes of eight *Bothrops* snake
venoms. Comp. Biochem. Physiol., Part D: Genomics
Proteomics.

[67] Antunes T. C., Yamashita K. M., Barbaro K. C., Saiki M., Santoro M. L. (2010). Comparative
analysis of newborn and adult *Bothrops jararaca* snake
venoms. Toxicon.

[68] Santoro M. L., Sousa-e-Silva M. C. C., Gonçalves L. R.
C., Almeida-Santos S. M., Cardoso D. F., Laporta-Ferreira I. L., Saiki M., Peres C. A., Sano-Martins I. S. (1999). Comparison of the biological activities in venoms from
three subspecies of the South American rattlesnake (*Crotalus
durissus terrificus*, *C. durissus cascavella* and *C. durissus collilineatus*). Comp. Biochem. Physiol. C Pharmacol. Toxicol. Endocrinol..

[69] Gomes P. C., Machado de Ávila R. A., Selena Maria W., Richardson M., Fortes-Dias C. L., Chávez-Olórtegui C. (2007). The co-purification
of a lectin (BJcuL) with phospholipases A2 from *Bothrops jararacussu* snake venom by immunoaffinity chromatography with antibodies to
crotoxin. Toxicon.

[70] Oliveira S. C., Fonseca F. V., Antunes E., Camargo E. A., Morganti R. P., Aparício R., Toyama D. O., Beriam L. O., Nunes E. V., Cavada B. S., Nagano C. S., Sampaio A. H., Nascimento K. S., Toyama M. H. (2008). Modulation of the pharmacological effects of enzymatically-active
PLA2 by BTL-2, an isolectin isolated from the Bryothamnion triquetrum
red alga. BMC Biochem.

[71] Lai C. C., Her G. R. (2002). Analysis of N-glycosylation of phospholipase A2 from
venom of individual bees by microbore high-performance liquid chromatography–electrospray
mass spectrometry using an ion trap mass spectrometer. J. Chromatogr. B: Biomed. Sci. Appl.

[72] Valentin E., Lambeau G. (2000). What can venom phospholipases
A2 tell us about the
functional diversity of mammalian secreted phospholipases A2?. Biochimie.

[73] Nishimoto K., Tanaka K., Murakami T., Nakashita H., Sakamoto H., Oguri S. (2015). Datura stramonium agglutinin: Cloning,
molecular characterization and recombinant production in Arabidopsis
thaliana. Glycobiology.

[74] Lochnit G., Geyer R. (1995). Carbohydrate structure
analysis of batroxobin, a thrombin-like serine
protease from Bothrops moojeni venom. Eur. J.
Biochem.

[75] Khoo K. H. (2021). A mass
spectrometry-based glycotope-centric cellular glycomics is the more
fruitful way forward to see the forest for the trees. Biochem. Soc. Trans.

[76] Roth J., Zuber C., Park S., Jang I., Lee Y., Kysela K. G., Le Fourn V., Santimaria R., Guhl B., Cho J. W. (2010). Protein *N*-Glycosylation,
Protein Folding, and Protein Quality Control. Mol. Cells.

[77] Adams B. M., Oster M. E., Hebert D. N. (2019). Protein
Quality Control in the Endoplasmic
Reticulum. Protein J.

[78] Paes
Leme A. F., Kitano E. S., Furtado M. F., Valente R. H., Camargo A. C., Ho P. L., Fox J. W., Serrano S. M. (2009). Analysis
of the subproteomes of proteinases and heparin-binding toxins of eight
Bothrops venoms. Proteomics.

[79] Dias G. S., Kitano E. S., Pagotto A. H., Sant’anna S. S., Rocha M. M., Zelanis A., Serrano S. M. (2013). Individual Variability
in the Venom Proteome of Juvenile Bothrops jararaca Specimens. J. Proteome Res.

[80] Smith C. F., Nikolakis Z. L., Ivey K., Perry B. W., Schield D. R., Balchan N. R., Parker J., Hansen K. C., Saviola A. J., Castoe T. A. (2023). Snakes on a plain: biotic and abiotic factors
determine venom compositional variation in a wide-ranging generalist
rattlesnake. BMC Biol.

[81] Clerc F., Reiding K. R., Jansen B. C., Kammeijer G. S. M., Bondt A., Wuhrer M. (2016). Human plasma protein N-glycosylation. Glycoconjugate J..

[82] Steirer L. M., Park E. I., Townsend R. R., Baenziger J. U. (2009). The Asialoglycoprotein
Receptor Regulates Levels of Plasma Glycoproteins Terminating with
Sialic Acid α2,6-Galactose *. J. Biol.
Chem.

[83] Monroe R. S., Huber B. E. (1994). The major form of the murine asialoglycoprotein receptor:
cDNA sequence and expression in liver, testis and epididymis. Gene.

[84] Zhang Y., Mao Y., Zhao W., Su T., Zhong Y., Fu L., Zhu J., Cheng J., Yang H. (2020). Glyco-CPLL: An Integrated Method
for In-Depth and Comprehensive N-Glycoproteome Profiling of Human
Plasma. J. Proteome Res.

[85] Ma B., Simala-Grant J. L., Taylor D. E. (2006). Fucosylation in prokaryotes and eukaryotes. Glycobiology.

[86] Nishima W., Miyashita N., Yamaguchi Y., Sugita Y., Re S. (2012). Effect of
Bisecting GlcNAc and Core Fucosylation on Conformational Properties
of Biantennary Complex-Type N-Glycans in Solution. J. Phys. Chem. B.

[87] Post Y., Puschhof J., Beumer J., Kerkkamp H. M., de Bakker M. A. G., Slagboom J., de Barbanson B., Wevers N. R., Spijkers X. M., Olivier T. (2020). Snake
Venom Gland Organoids. Cell.

[88] Luna M. S., Valente R. H., Perales J., Vieira M. L., Yamanouye N. (2013). Activation
of *Bothrops jararaca* snake venom gland and venom
production: A proteomic approach. J. Proteomics.

[89] Wu D., Struwe W. B., Harvey D. J., Ferguson M. A. J., Robinson C. V. (2018). N-glycan
microheterogeneity regulates interactions of plasma proteins. Proc. Natl. Acad. Sci. U. S. A.

[90] Stanley, P. ; Moremen, K. ; Lewis, N. ; Taniguchi, N. ; Aebi, M. N-glycans. In Essentials of Glycobiology; Cold Spring Harbor Laboratory Press, 2022. 35536922

[91] Fox J. W., Ma L., Nelson K., Sherman N. E., Serrano S. M. T. (2006). Comparison of
indirect and direct approaches using ion-trap and Fourier transform
ion cyclotron resonance mass spectrometry for exploring viperid venom
proteomes. Toxicon.

[92] Sousa L. F., Portes-Junior J. A., Nicolau C. A., Bernardoni J. L., Nishiyama M. Y., Amazonas D. R., Freitas-de-Sousa L. A., Mourão R. H., Chalkidis H. M., Valente R. H. (2017). Functional
proteomic analyses of *Bothrops atrox* venom reveals
phenotypes associated with habitat variation in the Amazon. J. Proteomics.

[93] Ogawa T., Oda-Ueda N., Hisata K., Nakamura H., Chijiwa T., Hattori S., Isomoto A., Yugeta H., Yamasaki S., Fukumaki Y., Ohno M., Satoh N., Shibata H. (2019). Alternative
mRNA Splicing in Three Venom Families Underlying a Possible Production
of Divergent Venom Proteins of the Habu Snake, Protobothrops flavoviridis. Toxins.

[94] Melani R. D., Nogueira F. C. S., Domont G. B. (2017). It is time for top-down venomics. J. Venomous Anim. Toxins Incl. Trop. Dis..

[95] Nicolau C. A., Carvalho P. C., Junqueira-de-Azevedo I. L. M., Teixeira-Ferreira A., Junqueira M., Perales J., Neves-Ferreira A. G., Valente R. H. (2017). An in-depth snake
venom proteopeptidome characterization:
Benchmarking *Bothrops jararaca*. J. Proteomics.

[96] Kelleher D. J., Gilmore R. (2006). An evolving view of
the eukaryotic oligosaccharyltransferase. Glycobiology.

[97] Augusto-de-Oliveira C., Stuginski D. R., Kitano E. S., Andrade-Silva D., Liberato T., Fukushima I., Serrano S. M., Zelanis A. (2016). Dynamic Rearrangement
in Snake Venom Gland Proteome: Insights into Bothrops jararaca Intraspecific
Venom Variation. J. Proteome Res.

[98] Nikai T., Taniguchi K., Komori Y., Masuda K., Fox J. W., Sugihara H. (2000). Primary Structure and Functional Characterization of
Bilitoxin-1, a Novel Dimeric P-II Snake Venom Metalloproteinase from *Agkistrodon bilineatus* Venom. Arch.
Biochem. Biophys.

[99] Geyer A., Fitzpatrick T. B., Pawelek P. D., Kitzing K., Vrielink A., Ghisla S., Macheroux P. (2001). Structure and characterization of
the glycan moiety of L-amino-acid oxidase from the Malayan pit viper
Calloselasma rhodostoma. Eur. J. Biochem.

[100] Wang Y. M., Tsai I. H., Chen J. M., Cheng A. C., Khoo K. H. (2014). Correlation
between the Glycan Variations and Defibrinogenating
Activities of Acutobin and Its Recombinant Glycoforms. PLoS One.

[101] Angata T., Varki V. A. (2023). Discovery, classification, evolution
and diversity of Siglecs. Mol. Aspects Med.

[102] Andrade-Silva D., Nishiyama M. Y., Stuginski D. R., Zelanis A., Serrano S. M. T. (2021). The distinct
N-terminomes of *Bothrops jararaca* newborn and adult
venoms. Biochim. Biophys. Acta, Proteins Proteomics.

[103] Remacle A. G., Chekanov A. V., Golubkov V. S., Savinov A. Y., Rozanov D. V., Strongin A. Y. (2006). O-Glycosylation
Regulates Autolysis
of Cellular Membrane Type-1 Matrix Metalloproteinase (MT1-MMP) *. J. Biol. Chem.

[104] Dumez M. E., Teller N., Mercier F., Tanaka T., Vandenberghe I., Vandenbranden M., Devreese B., Luxen A., Frère J. M., Matagne A., Jacquet A., Galleni M., Chevigné A. (2008). Activation
Mechanism of Recombinant Der p 3 Allergen
Zymogen. J. Biol. Chem.

[105] Goettig P. (2016). Effects of Glycosylation on the Enzymatic
Activity
and Mechanisms of Proteases. Int. J. Mol. Sci.

[106] Boon L., Ugarte-Berzal E., Vandooren J., Opdenakker G. (2016). Glycosylation of matrix metalloproteases
and tissue
inhibitors: present state, challenges and opportunities. Biochem. J.

[107] Kumar S., Cieplak P. (2018). Role of N-glycosylation
in activation
of proMMP-9. A molecular dynamics simulations study. PLoS One.

[108] Serrano S. M., Kim J., Wang D., Dragulev B., Shannon J. D., Mann H. H., Veit G., Wagener R., Koch M., Fox J. W. (2006). The cysteine-rich
domain of snake
venom metalloproteinases is a ligand for von Willebrand factor A domains:
role in substrate targeting. J. Biol. Chem.

[109] Serrano S. M. T., Wang D., Shannon J. D., Pinto A. F. M., Polanowska-Grabowska R. K., Fox J. W. (2007). Interaction of the
cysteine-rich domain of snake venom metalloproteinases with the A1
domain of von Willebrand factor promotes site-specific proteolysis
of von Willebrand factor and inhibition of von Willebrand factor-mediated
platelet aggregation. FEBS J.

[110] Menezes M. C., Paes Leme A. F., Melo R. L., Silva C. A., Della Casa M., Bruni F. M., Lima C., Lopes-Ferreira M., Camargo A. C., Fox J. W., Serrano S. M. (2008). Activation of leukocyte
rolling by the cysteine-rich domain and the hyper-variable region
of HF3, a snake venom hemorrhagic metalloproteinase. FEBS Lett.

[111] Serrano S. M. T., Maroun R. C. (2005). Snake venom serine
proteinases: sequence
homology vs. substrate specificity, a paradox to be solved. Toxicon.

[112] Paes Leme A. F., Prezoto B. C., Yamashiro E. T., Bertholim L., Tashima A. K., Klitzke C. F., Camargo A. C., Serrano S. M. (2008). Bothrops protease A, a unique highly glycosylated serine
proteinase, is a potent, specific fibrinogenolytic agent. J. Thromb. Haemostasis.

[113] Fox J. W. (2013). A brief review of the scientific
history of several
lesser-known snake venom proteins: l-amino acid oxidases, hyaluronidases
and phosphodiesterases. Toxicon.

[114] Moustafa I. M., Foster S., Lyubimov A. Y., Vrielink A. (2006). Crystal Structure
of LAAO from *Calloselasma rhodostoma* with an l-Phenylalanine
Substrate: Insights into Structure and Mechanism. J. Mol. Biol.

[115] Aird S. D. (2002). Ophidian envenomation strategies
and the role of purines. Toxicon.

[116] McCue M. D. (2006). Cost of Producing Venom in Three
North American Pitviper
Species. Copeia.

[117] Pintor A. F. V., Krockenberger A. K., Seymour J. E. (2010). Costs of venom production
in the common death adder (*Acanthophis antarcticus*). Toxicon.

[118] Smith M. T., Ortega J., Beaupre S. J. (2014). Metabolic
cost of
venom replenishment by Prairie Rattlesnakes (*Crotalus viridis
viridis*). Toxicon.

