# Supplementary material for: N‑Glycoproteomic Portraits of Bothrops Snake Venoms Reveal Evolutionarily Conserved and Divergent Phenotypes
Source: J Proteome Res. 2025 Nov 7;24(12):5948–72. doi: 10.1021/acs.jproteome.5c00249 (PMC12687316; doi:10.1021/acs.jproteome.5c00249)
Supplement: Supplementary file 1 [file pr5c00249_si_001.pdf]

N-glycoproteomic portraits of *Bothrops* snake venoms reveal evolutionarily conserved and divergent phenotypes.

Débora Andrade-Silva[a], Lívia Rosa-Fernandes[b], Marcelo S. Reis[c], Alison F. A. Chaves[a], Dilza Trevisan-Silva[a], Silvia R. T. Cardoso[d], Giuseppe Palmisano[e,f,] Martin R. Larsen[b]*, Solange M. T. Serrano[a]*.

[a]Laboratory of Applied Toxinology, Center of Toxins, Immune-Response, and Cell Signaling (CeTICS), Butantan Institute, São Paulo, Brazil.
[b]Department of Biochemistry and Molecular Biology, University of Southern Denmark, Odense, Denmark.
[c]Institute of Computing, Unicamp, Campinas, Brazil.
[d]Biological Museum, Butantan Institute, São Paulo, Brazil.
[e]Department of Parasitology, Institute of Biomedical Science, University of São Paulo, São Paulo, Brazil.
[f] School of Natural Sciences, Macquarie University, Sydney, Australia.
* Corresponding authors

Supplementary Information

Experimental Procedures (pages S4 – S16)

1. Venom samples

2. Venom protein profile and carbohydrate content determination

3. Analysis of venom protein glycosylation profiles by lectin blot

4. Identification of glycosylation sites in venom proteins by mass spectrometry

5. Protein quantification and digestion with Lys-C and trypsin

6. Glycopeptide enrichment by interaction with titanium dioxide (TiO_2)

7. Enzymatic de-*N*-glycosylation

8. Glycopeptide enrichment by HILIC

9. Glycopeptide fractionation using high-pH reversed-phase chromatography

10. LC-MS/MS analysis of de-*N*-glycosylated peptides (dF1, dF2, and dF3 fractions)

11. LC-MS/MS analysis of intact glycopeptides (gF1, gF2.1, gF2.2, gF2.3, and gF3 fractions)

12. LC-MS/MS analysis of non-glycosylated peptide fractions

13. Data analysis of de-*N*-glycosylated and non-glycosylated peptide fractions

14. Data analysis of intact *N*-glycopeptides

15. Clustering analyses

Supplementary Figures S1 – S27 (pages S16 – S44)

Supplementary Figure S1. Electrophoretic profiles of *Bothrops* venoms

Supplementary Figure S2. Determination of carbohydrate content in *Bothrops* venoms

Supplementary Figure S3. Profile of proteins of *Bothrops* venoms recognized by lectin blot using digoxigenin-labeled MAA lectin.

Supplementary Figure S4. Profile of proteins of *Bothrops* venoms recognized by lectin blot using digoxigenin-labeled SNA lectin.

Supplementary Figure S5. Profile of proteins of *Bothrops* venoms recognized by lectin blot using digoxigenin-labeled DSA lectin.

Supplementary Figure S6. Profile of the proteins of *Bothrops* venoms recognized by lectin blot using digoxigenin-labeled GNA lectin.

Supplementary Figure S7. Profile of proteins of *Bothrops* venoms recognized by lectin blot using digoxigenin-labeled PNA lectin.

Supplementary Figure S8. Extracted ion chromatograms and distribution of peptide m/z values of LC-MS/MS analysis of de-*N*-glycosylated fractions of *Bothrops* venoms.

Supplementary Figure S9. Numbers of missed cleavages detected in LC-MS/MS analysis of de-*N*-glycosylated fractions of *Bothrops* venoms.

Supplementary Figure S10. Venn diagrams of the number of peptides identified in the three replicates of de-*N*-glycosylated fractions of *Bothrops* venoms.

Supplementary Figure S11. Venn diagrams of the number of peptides identified in each of the three replicates of de-*N*-glycosylated fractions of *Bothrops* venoms.

Supplementary Figure S12. Number of peptides identified in at least two replicates of LC-MS/MS analysis of de-*N*-glycosylated fractions (dF1, dF2 and dF3) of *Bothrops* venoms.

Supplementary Figure S13. Summary of peptide identifications in the de-*N*-glycosylated fractions of *Bothrops* venoms

Supplementary Figure S14. Intersections of identified putative *N*-glycosylated peptide sequences in *Bothrops* venoms.

Supplementary Figure S15. Comparison of the number of putative *N*-glycosylated peptides and putative *N*-glycosylation sites identified in de-*N*-glycosylated fractions.

Supplementary Figure S16. Number of the *N*-glycosylation sites containing the sequons NXS and NXT identified in the intact *N*-glycosylated peptides.

Supplementary Figure S17. Analysis of the distribution of *N*-glycosylated peptides identified in the intact glycopeptide fractions, according to toxin class and *Bothrops* species.

Supplementary Figure S18. Analysis of *N*-glycan classes identified in *Bothrops* venoms.

Supplementary Figure S19. Distribution of intact *N*-glycosylated peptides identified in *Bothrops* venoms, by toxin class, and the proportion of identified peptides that contain *N*-glycan chains of high-mannose or hybrid/complex types.

Supplementary Figure S20. MS/MS spectra of an intact acetylated *N*-glycopeptide identified in *B. insularis* venom.

Supplementary Figure S21. Comparison of the low mass range of MS/MS spectra of an intact *N*-glycopeptide identified in *B. cotiara*, *B. jararaca*, and *B. jararacussu* venoms.

Supplementary Figure S22. Overview of the most often *N*-glycan compositions identified in the seven *Bothrops* venoms analyzed in this study.

Supplementary Figure S23. Extracted ion chromatograms and distribution of peptide m/z values of LC-MS/MS analysis of non-glycosylated fractions of *Bothrops* venoms.

Supplementary Figure S24. Numbers of missed cleavages detected in LC-MS/MS analysis of non-glycosylated fractions of *Bothrops* venoms.

Supplementary Figure S25. Venn diagrams of the number of peptides identified in the three replicates of non-glycosylated peptide fractions of *Bothrops* venoms.

Supplementary Figure S26. Number of peptides identified in at least two replicates of LC-MS/MS analysis of non-glycosylated fractions of *Bothrops* venoms.

Supplementary Figure S27. *Bothrops* venom clustering according to the amino acid backbones of glycosylated peptides or non-glycosylated peptides.

Supplementary Tables S1 – S40 (Supplementary tables S1 - S40.zip)

Supplementary Table S1. Data from the measurement of carbohydrate content in *Bothrops* venoms by the phenol sulfuric method.

Supplementary Table S2A. Nomenclature of samples submitted to mass spectrometric analysis and their descriptions.

Supplementary Table S2B. Description of mass spectrometry raw files.

Supplementary Tables S3 – S9. Peptide identification of the de-*N*-glycosylated fractions (dF1, dF2, dF3) of *Bothrops* venoms by LC-MS/MS.

Supplementary Table S10. Comparison of the total number of peptides identified in the de-*N*-glycosylated peptide fraction (dF1, dF2, and dF3) of each venom.

Supplementary Tables S11 – S17. List of the putative *N*-glycosylated peptides identified in the de-*N*-glycosylated fractions (dF1, dF2, dF3) of *Bothrops* venoms and their related *N*-glycosylated sites.

Supplementary Tables S18 – S24. *N*-glycopeptides identified in the intact glycopeptide fractions of *Bothrops* venoms using the GlycReSoft software.

Supplementary Table S25. *O*-acetylated *N*-glycosylated peptides identified in the seven *Bothrops* venoms.

Supplementary Table S26. *N*-glycosylation heterogeneity ratios of toxin classes identified in *Bothrops* venoms.

Supplementary Tables S27 – S33. Peptide identification of the non-glycosylated peptide fractions of *Bothrops* venoms by LC-MS/MS.

Supplementary Tables S34 – S40. Protein groups identified in at least two replicates of the non-glycosylated peptide fraction of *Bothrops* venoms.

References (page S45-S46)

Experimental procedures

1. Venom samples

Lyophilized venom of *B. jararaca*, *B. insularis*, *B. cotiara*, *B. jararacussu*, *B. moojeni*, and *B. fonsecai*, was provided by Laboratory of Herpetology, Butantan Institute, São Paulo, Brazil. Pools were composed of venom extractions from at least ten individuals of each species. The venom of *B. alcatraz*, was extracted from six specimens and lyophilized. Brazilian Sisgen (*Sistema Nacional de Gestão do Patrimônio Genético e do Conhecimento Tradicional Associado*) licenses: A0AE234, A362095, AD1F726, AE8C064, AC2B2CE, A318EB8, and A5F9985.

2. Venom protein profile and carbohydrate content determination

Bothrops venom solutions were prepared in 150 mM sodium chloride, and protein concentration was determined by the Bradford assay[119], using bovine serum albumin as standard protein, and the procedure followed the manufacturer's instructions (Sigma). For protein profile visualization, venoms were analyzed by sodium dodecyl sulfate polyacrylamide gel electrophoresis (SDS-

PAGE), as described[120]. Carbohydrate content was quantified by the phenol-sulfuric method[121] using reagents of the kit Total Carbohydrate Quantification Assay (Abcam) and following the manufacturer's instructions. Comparisons of absorbance values were performed against a glucose standard curve, and the quantification was expressed as glucose equivalent. The statistical analysis was performed using the One-way ANOVA.

3. Analysis of venom protein glycosylation profiles by lectin blot

Bothrops venoms were submitted to lectin blot analysis using five different lectins labeled with digoxigenin. Lectin recognition was detected using an antibody against digoxigenin, labeled with alkaline phosphatase. The reagents of the Dig Glycan Differentiation Kit (Sigma) included the lectins: *Datura stramonium agglutinin* (DSA), *Galanthus nivalis agglutinin* (GNA), *Maackia amurensis agglutinin* (MAA), *Sambucus nigra agglutinin* (SNA), and *Peanut agglutinin* (PNA). These lectins bind to structures present in both *N*- and *O*-glycans. DSA recognizes the disaccharide Gal-1,4-GlcNAc or GlcNAc oligomers. GNA recognizes terminal mannose units linked to another mannose unit in the positions 3, 6 or 2. MAA and SNA, recognize glycans with sialic acid in linkages, respectively, α2-3- and α2-6-, to a galactose unit. PNA recognizes a disaccharide composed by Gal-1,3-GalNAc. All lectin solutions were prepared according to the manufacturer's instructions.

4. Identification of glycosylation sites in venom proteins by mass spectrometry

To identify *N*-glycosylation sites in glycoproteins of *Bothrops* venoms, proteins were digested with Lys-C and trypsin and subsequently submitted to a tandem glycopeptide enrichment and LC-MS/MS analysis. The first enrichment method used was based on the affinity of sialylated

glycopeptides to titanium dioxide (TiO_2)[45,122], followed by hydrophilic interaction chromatography (HILIC). The elution fractions from the TiO_2 enrichment were designated glycopeptide fraction #1 (F1) and glycopeptide fraction #2 (F2), and the elution fraction from HILIC was designated glycopeptide fraction #3 (F3).

For the analysis of de-N-glycosylated peptides, 1/10 of the volume of the glycopeptide-enriched fractions (F1–F3) was submitted to enzymatic de-*N*-glycosylation using PNGase F and the deamidated peptides containing the *N*-glycosylation sequon (NXS/T) were used to compose a database for the identification of intact glycopeptides. As the glycopeptide fractions were divided in two parts, the part submitted to de-*N*-glycosylation received the prefix "d" while the part containing the intact glycopeptides received the prefix "g". Figure 1 outlines the scheme of sample preparation workflow and LC-MS/MS analysis, and Supplementary Table S2A summarizes the adopted nomenclature for the samples and their descriptions. The entire experiment was performed in three technical replicates for each venom. Supplementary Table S2B describes the raw files of mass spectrometric analyses.

5. Protein quantification and digestion with Lys-C and trypsin

Venom protein solutions were prepared in 150 mM sodium chloride and protein concentration was determined by fluorescence using Qubit Protein Assay kit (Thermo Fischer Scientific). For each experiment, 600 µg of venom proteins were denatured using 5.5 M urea, 1.8 M thiourea, and the disulfide bonds were reduced by incubation with 10 mM dithiothreitol (DTT) for 30 min at room temperature. The cysteine residues were carbamidomethylated by incubation with 40 mM iodoacetamide (IAA) for 30 min at room temperature in the dark. In the first digestion step, 0.05 AU (activity unit) of Lys-C (Wako) was added and the mixture was further incubated for 4 h at 30

ºC. Then, protein solutions were diluted with 20 mM triethylammonium bicarbonate buffer (TEAB) (Sigma) before adding methylated trypsin in a 1:60 (m/m) ratio, and the mixtures were incubated at 30 ºC for 18 h. After the confirmation of trypsin digestion, by peptide mass profiling using a MALDI-ToF mass spectrometer (Bruker), the reaction was stopped with 1% trifluoroacetic acid (TFA). The peptide solutions were concentrated to 150 μL by vacuum centrifugation and then directly used as starting material for the first glycopeptide enrichment step.

6. Glycopeptide enrichment by interaction with titanium dioxide (TiO_2)

For the interaction of glycopeptides with TiO_2, the concentrated peptide solutions were diluted with TiO_2 loading solution (80% acetonitrile (ACN)/5% TFA and 1 M glycolic acid) to a final volume of 1 mL. TiO_2 beads (GL Sciences) were added to the peptide solutions using the proportion of 0.6 mg of TiO_2 beads to 100 μg of initial protein mass, and the mixtures were incubated on a mixer for 20 min at room temperature (IKA Vibrax VXR basic). After centrifugation at 2,000 x g for 5 min to decant the beads, the supernatants were transferred to new microtubes containing 50% of TiO_2 beads previously used, for another similar incubation step. After centrifugation, the supernatants were collected from the second tube containing TiO_2 beads and designated as TiO_2 unbound fractions.

The TiO_2 beads from the two enrichment steps were washed with 100 μL of loading solution, and then pooled together into new microtubes. After a new centrifugation step, these new supernatants were collected and mixed with the previously collected TiO_2 unbound fractions. The last washing step was performed with 100 μL of 80% ACN/1% TFA and the resulting supernatant was added to compose the TiO_2 unbound fraction.

The fraction of glycopeptides bound to the TiO_2 beads was eluted in two steps. In the first, elution was performed with 100 µL of 10% ACN/0.1% TFA and the eluate was designated as glycopeptide fraction #1 (F1). After drying the beads by vacuum centrifugation for 10 min, for the second elution step, the beads were mixed with 150 µL of 1% Triethyl-amine (TEA) and shaken on a mixer for 20 min. After centrifugation, the supernatant was collected and designated as glycopeptide fraction #2 (F2). After that, the beads were washed with 30 µL of 1% TEA and 65 µL of 30% ACN and these eluates were added to the F2 fraction.

7. Enzymatic de-*N*-glycosylation

For the analysis of de-*N*-glycosylated peptides, 1/10 of the volume of the F1 and F2 fractions were dried by vacuum centrifugation and dissolved in 30 µL of 20 mM TEAB, and 200 U of PNGase F (New England Biolabs) and 0.5 mU of sialidase A (Prozyme) were added. The reactions were incubated for 18 h at 37 ºC. The de-N-glycosylated samples were dried and analyzed by LC-MS/MS, as described below. These de-*N*-glycosylated fractions were named dF1 and dF2, while the non-de-*N*-glycosylated part of fractions F1 and F2 were named gF1 and gF2. Fractions gF1 were submitted to LC-MS/MS analysis as described below, while fractions gF2 were submitted to a fractionation step using high pH reversed-phase.

8. Glycopeptide enrichment by HILIC

For a second round of glycopeptide enrichment, the TiO_2 unbound fractions were desalted using Oligo R2/Oligo R3 tip microcolumns prepared in house using a C18 disk membrane (3M Empore) and a slurry of 50/50 mixture of Oligo R2 /OligoR3 resin (Applied Biosystems). For that, the acetonitrile content was removed by vacuum centrifugation and the samples were loaded on the

microcolumns previously equilibrated with 0.1% TFA. To ensure the complete removal of glycolic acid and urea, the peptides were washed four times with 0.1% TFA and eluted with 60% ACN/0.1% TFA.

After desalting, the peptides were dried by vacuum centrifugation and dissolved in 100 µL of 80% ACN/1% TFA prior to loading into HILIC tip microcolumns packaged in house in p200 tips, using a membrane of C8 (3M Empore) and 40 µL of HILIC resin slurry (14 mg of resin in 100 µL 1% TFA) (Polyhydroxyethyl A, 12 µm, 300 Å) (PolyLC Inc.). The samples were loaded on the microcolumn, the eluate was collected and re-loaded. This procedure was repeated four times and the microcolumn was washed once with 100 µL of 80% ACN/1% TFA. Glycopeptides bound to the resin were eluted in three sequential steps using 100 µL 0.1% TFA, 20 mM TEAB and 50% ACN, and all collected volumes were pooled together to compose the fraction designated as glycopeptide fraction #3 (F3). As described for the glycopeptide enrichment by TiO_2, 1/10 of the volume of the HILIC fractions was submitted to enzymatic de-*N*-glycosylation (dF3 fractions), as described above. The remaining volume of the glycopeptide fractions (gF3) was dried and submitted to LC-MS/MS as described below. The peptides present in the HILIC unbound fractions (flow-through plus the washing volume) were quantified by the Qubit Protein Assay kit (Thermo Fischer Scientific). The fractions containing the peptides that did not bind to TiO_2 and were not retained in HILIC were designated as non-glycosylated peptide fractions and submitted to LC-MS/MS analysis as described below.

9. Glycopeptide fractionation using high-pH reversed-phase chromatography

The second fraction from the TiO_2 enrichment, gF2, was further submitted to high-pH reversed-phase fractionation in a tip microcolumn of Oligo R2/R3, packaged as described in Glycopeptide

Enrichment by HILIC. In this step, buffer A was 20 mM ammonium formate, pH 9.3, and buffer B was composed of 80% ACN/20% buffer A. The pH of samples was adjusted to pH 9 using a 12.5% ammonium hydroxide solution prior to loading to the microcolumn and each sample was fractionated in three parts: i) the first (gF2.1) was composed by the flow-through eluted with buffer A and the glycopeptides eluted with 25% of buffer B; ii) the second (gF2.2) was eluted with 50% of buffer B; and the third (gF.2.3) was eluted with 100% of buffer B. All fractions were dried by vacuum centrifugation. These fractions were submitted to LC-MS/MS analysis as described below.

10. LC-MS/MS analysis of de-*N*-glycosylated peptides (dF1, dF2, and dF3 fractions)

The fractions of de-*N*-glycosylated peptides (dF1, dF2, and dF3 fractions) were dissolved in 5.5 µL of 0.1% formic acid (FA) and 5 µL were analyzed by LC-MS/MS. Samples were loaded onto a 3.5 cm C18 precolumn (100 µm I.D. x 360 µm O.D.) in an EASY-nLC system (Thermo). The peptide separations were performed using a 20 cm C18 analytical column (75 µm I.D. x 360 µm O.D.), packed with ReproSil-Pur C18 AQ 3 µm (Dr. Maisch). Peptide elution followed a gradient composed by 0.1% FA, as phase A and 95% ACN/0.1% FA, as phase B, with a constant flow rate of 250 nL/min. Elution started with a linear gradient of 7%-30% B in 10 min, followed by 50% B for 10 min and a final increase to 100% B in 5 min, remaining in this condition for 8 min. The nLC system was in tandem with a Q-Exactive HF mass spectrometer (Thermo) operating in the positive mode. Data-dependent acquisition was selected, and the 15 most intense ions were selected for MS/MS analysis. The automatic gain control (AGC) was set to 3 x 10^6 ions and a maximum fill time of 100 ms, while the mass-range was fixed to 400-1800 m/z with high resolution (45,000 full width half maximum (FWHM) at m/z 200). For peptide fragmentation, higher energy collision-induced dissociation (HCD) was applied, using a normalized collision energy (NCE) of 28. The

fragmentation step was performed at 15,000 FWHM resolution with AGC target of 1 x 10^5 and with a maximum injection time of 60 ms using an isolation window of 1.2 m/z. The intensity threshold for MS/MS selection was set to 8.3 x 10^4 and dynamic exclusion of 15 s.

11. LC-MS/MS analysis of intact glycopeptides (gF1, gF2.1, gF2.2, gF2.3, and gF3 fractions)

The fractions of intact glycopeptides (gF1, gF2.1, gF2.2, gF2.3, and gF3 fractions) were dissolved in 10 µL of 0.1% FA and 9.5 µL were analyzed by LC-MS/MS. Samples were loaded onto a 3.5 cm C18 precolumn (100 µm I.D. x 360 µm O.D.) in an EASY-nLC system (Thermo). The peptide separations were performed using a 20 cm C18 analytical column, packed with ReproSil-Pur C18 AQ 3 µm (Dr. Maisch). Glycopeptide elution followed a gradient composed by 0.1% FA, as phase A, and 95% ACN/0.1% FA, as phase B, with a constant flow rate of 250 nL/min. Elution started with a linear gradient of 7%-28% B in 10 min, followed by 55% B for 16 min and a final increase to 100% B in 5 min, remaining in this condition for 10 min. The nLC system was in tandem with a Q-Exactive HF mass spectrometer (Thermo) operating in the positive mode. Data-dependent acquisition was selected, and the 10 most intense ions were selected for MS/MS analysis. The AGC was set to 3 x 10^6 ions and a maximum fill time of 120 ms, the mass-range was fixed to 700-2,000 m/z with high resolution (60,000 FWHM at m/z 200). For glycopeptide fragmentation, HCD was applied, using the stepped mode of NCE at 20 and 37. The fragmentation step was performed at high resolution (60,000 FWHM) with AGC target of 1 x 10^6 and with a maximum injection time of 400 ms using an isolation window of 2.0 m/z. The intensity threshold for MS/MS selection was set to 2.5 x 10^4 and a dynamic exclusion of 15 s.

12. LC-MS/MS analysis of non-glycosylated peptide fractions

The fractions of peptides that did not bind to TiO2 beads and to HILIC resin were desalted using Oligo R2/Oligo R3 tip microcolumns, and then dissolved in 300 µL of 0.1% FA. The final peptide solutions were quantified using Qubit Protein Assay kit and then samples corresponding to 1.5 µg were analyzed by LC-MS/MS. Samples were loaded onto a 3.5 cm C18 precolumn (100 µm I.D. x 360 µm O.D.) in an EASY-nLC system (Thermo). The peptide separations were performed using a 20 cm C18 analytical column, packed with ReproSil-Pur C18 AQ 3 µm (Dr. Maisch). Peptide elution followed a gradient composed by 0.1% FA, as phase A, and 95% ACN/0.1% FA, as phase B, with a constant flowrate of 250 nL/min. Elution started with a linear gradient of 4%-25% B in 30 min, followed by 50% B for 25 min and a final increase to 100% B in 10 min, remaining in this condition for 15 min. The nLC system was in tandem with a Q-Exactive HF mass spectrometer (Thermo) operating in the positive mode. Data-dependent acquisition was selected, and the 15 most intense ions were selected to the MS/MS analysis. The AGC was set to 3×10^6 ions and a maximum fill time of 100 ms, the mass-range was fixed to 400-1800 m/z with high resolution (45,000 FWHM at m/z 200). For fragmentation, HCD was applied, using the NCE at 28. The fragmentation step was performed at 15,000 FWHM resolution with AGC target of 5×10^5 and with a maximum injection time of 60 ms using an isolation window of 1.2 m/z. The intensity threshold for MS/MS selection was set to 8.3×10^4 and a dynamic exclusion of 15 s.

13. Data analysis of de-*N*-glycosylated and non-glycosylated peptide fractions

Database search for peptide identification of de-*N*-glycosylated and non-glycosylated peptide fractions was performed using MaxQuant software (version 1.6.14.0)[47]. All .raw files were submitted to database search against an optimized protein .fasta file containing non-redundant sequences from UniProt and snake venom and accessory gland transcriptomes (built in September

2019 with 111,653 sequences, as described previously[24]. Spectra of LC-MS/MS analyses were submitted to database search considering trypsin as enzyme, in a semi-specific mode. The carbamidomethylation of cysteine residues was considered as a static modification, while methionine oxidation and asparagine/glutamine deamidation were set as dynamic modifications. The search mode "match between runs" was enabled in all searches. A false discovery rate (FDR) < 1% was set at PSM (Peptide Spectrum Match) and protein levels. At peptide level, only those with PEP (Posterior Error Probability) lower than 0.05 were considered. All results were analyzed using the Perseus software (version 1.6.13).

For the analysis of the de-*N*-glycosylated fractions, in each venom, the peptides identified in all replicates and fractions were considered together. Since previously to LC-MS/MS runs these fractions were submitted to enzymatic de-*N*-glycosylation to remove the *N*-glycan chains attached to asparagine residues and converting them into aspartic acid residues, from the list of identified peptides we considered as deamidated those containing at least one asparagine residue within a *N*-glycosylation consensus sequence (NXS/T), and these peptides were named 'putative *N*-glycosylated peptides'. We also considered the peptides containing an *N*-glycosylation consensus sequence disrupted by proteolytic cleavage (deamidated peptides with C-terminus containing asparagine at the penultimate position). Information about the amino acid residue after the last residue in the peptide was recovered from the peptide.txt file. Further, the probability value for the localization of deamidation was considered for peptide classification. Only deamidated peptides containing a deamidated asparagine within an *N*-glycosylation consensus sequence with a probability higher or equal to 0.5 was classified as putative *N*-glycosylated peptides. Peptides identified with a deamidation event but without an asparagine residue within an *N*-glycosylation consensus sequence were named 'deamidated peptides'. All other identified peptides were named

‘non-*N*-glycosylated peptides’. For protein inference analysis in the de-*N*-glycosylated peptide fractions, the protein groups IDs were not considered, and all analysis were based only on the toxin class of the protein IDs. As the enrichment step was applied at peptide level, many protein sequences have not been covered by more than one identified peptide, and as the protein inference step considered all peptides for the final assignment, the IDs of the proteins can be biased for those containing more *N*-glycosylation sites. This is specifically critical for snake venoms, whose proteomes comprise many toxins with considerable level of similarity.

In the case of the non-glycosylated peptide fractions, only protein groups identified in at least two replicates and by at least two peptides (at least one unique peptide) were considered. For all uses, for protein count the column Majority ID from proteinGroups.txt file was considered.

14. Data analysis of intact *N*-glycopeptides

Database search for identification of intact *N*-glycopeptides (gF1, gF2.1, gF2.2, gF2.3, and gF3 fractions) was performed using GlycReSoft (version 0.4.11)[57,123]. The protein database was composed by a non-redundant list of the putative *N*-glycosylated peptides identified in the de-*N*-glycosylated fractions and a list of 52 *N*-glycan compositions described in a previous study[15]. In the searches, the same list of *N*-glycans was used for all species, while the putative *N*-glycosylated peptide list applied in the searches was specific to each venom.

For the search, there was a previous step for conversion of .raw files into the .mzML format using MS Convert[124]. In the sample loading process, the preset configurations for “LC-MS/MS Glycoproteomics” were used. The *N*-glycopeptide search space was created from the putative *N*-glycosylated peptide list, considering carbamidomethylation of the cysteine residues as fixed modification, methionine oxidation as variable modification, no enzyme specification, and only

one *N*-glycosylation event per peptide. MS1 and MS2 tolerances were set to 10 ppm, q-Value threshold to 0.05, and minimum oxonium threshold to 0.05. For variable adducts, up to two ammonium ions or one sodium ion were accepted. The options permute decoy glycan, include rare signature ions and model retention time were enabled during the search. To search for O-acetylated sialylated structures, 42 Da was considered as a static modification and was add to the mass of the unmodified sialylated *N*-glycan in the database.

Network analyses were done in R 4.4.3 using the igraph and arcdiagram packages[59].

15. Clustering analyses

All clustering analyses were performed using a Python-coded program in a Jupyter notebook. To this end, we used the following libraries: matplotlib, numpy, pandas, seaborn, and scipy. In total, three analyses were performed, each one producing one or more hierarchical clustering, using the clustermap class in the seaborn library, which in turn was set with the complete linkage and the Euclidean distance.

In the first analysis, lists containing the identified peptides and their associated glycan compositions for each venom were processed, generating two occurrence matrices: in the first one, the rows and columns correspond to, respectively, peptides and venoms, and a matrix entry (i,j) has value one if peptide i was found in the venom j, and zero otherwise. The second one was built in a similar fashion, with the difference that rows correspond to a pair (peptide, glycan composition). For each occurrence matrix, we built a hierarchical clustering which displays the data in a heatmap and also shows the yielded dendrogram.

A second analysis was carried out using the list of neutral masses generated by the software GlycReSoft during the preprocessing step of the .mzml files. These values were calculated based

on the ion chromatogram and considering the preset configuration for LC-MS/MS glycoproteomics. Neutral masses from monocharged ions were excluded and all others charge states were considered. The neutral masses had values between 1,398.43 and 12,889.62. In this analysis, we built an occurrence matrix, in which rows and columns are, respectively, neutral masses (with rounded values) and venoms. A matrix entry (i,j) is a log base 2 of the counts of the number of neutral masses with value v_i in the venom j. Hierarchical clustering was then carried out.

Finally, for the analysis of the non-glycosylated peptides, we processed a list of peptides and venoms in a similar way to the first analysis, generating an occurrence matrix. That matrix was then used to perform hierarchical clustering.

Supplementary Figures

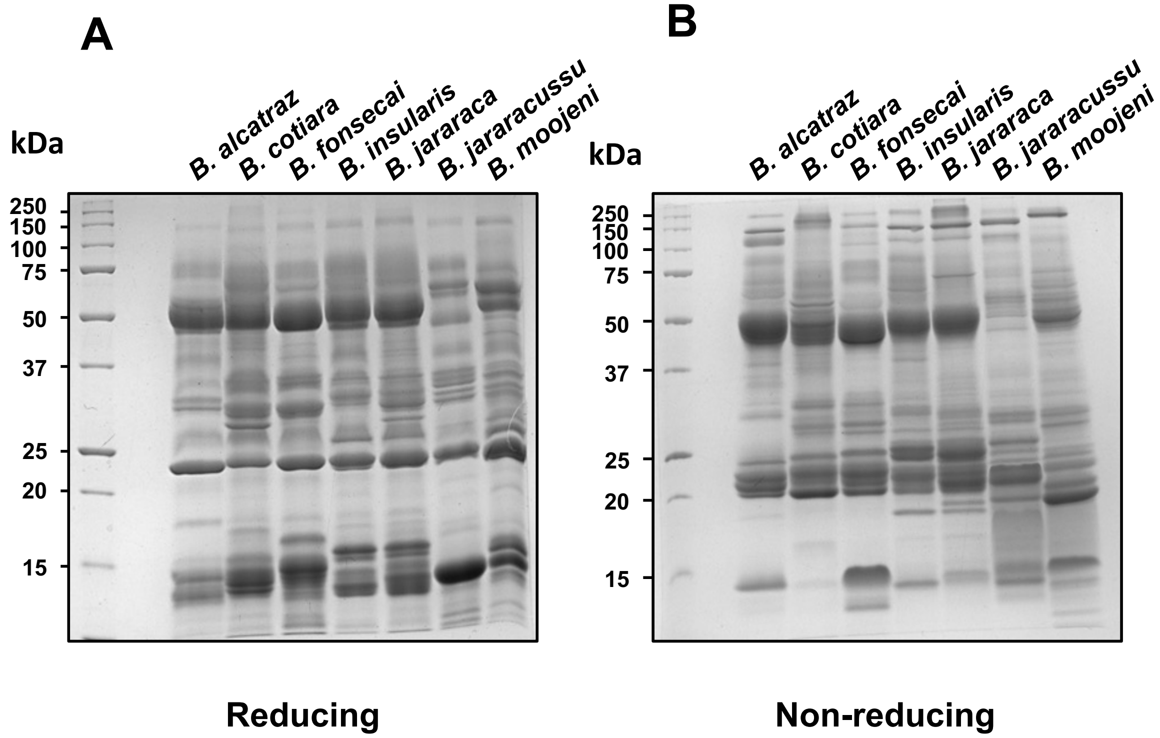

Supplementary Figure S1. Electrophoretic profiles of *Bothrops* venoms (30 µg) by SDS-PAGE (12% SDS-polyacrylamide gel) under reducing (A) and non-reducing conditions (B). Staining by colloidal Coomassie.

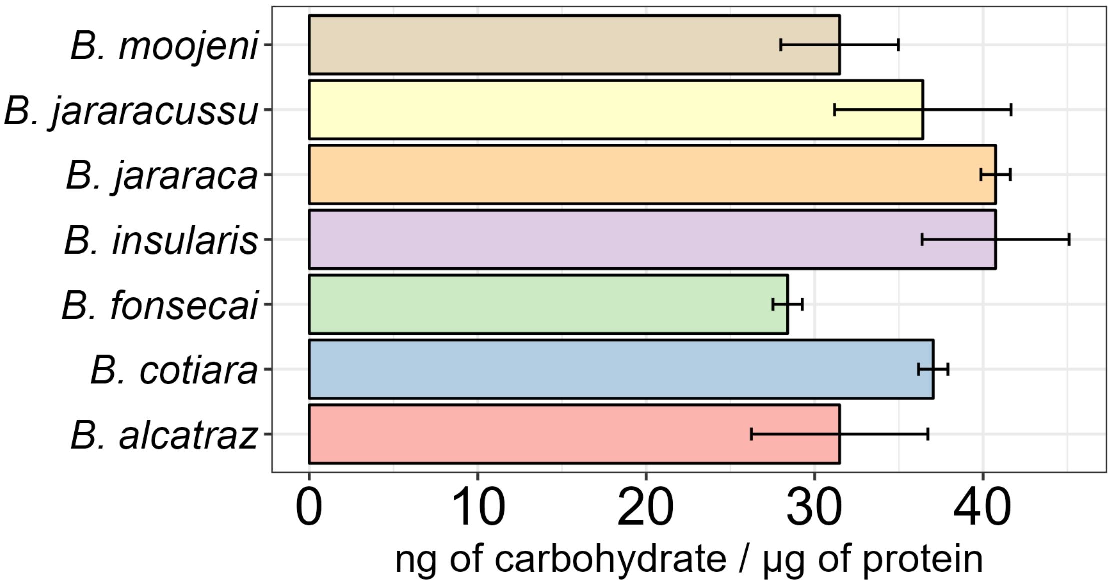

Supplementary Figure S2. Determination of carbohydrate content in *Bothrops* venoms. Bar graph of the values obtained in the determination of carbohydrate content using 300 µg of protein. The bars represent the standard deviation.

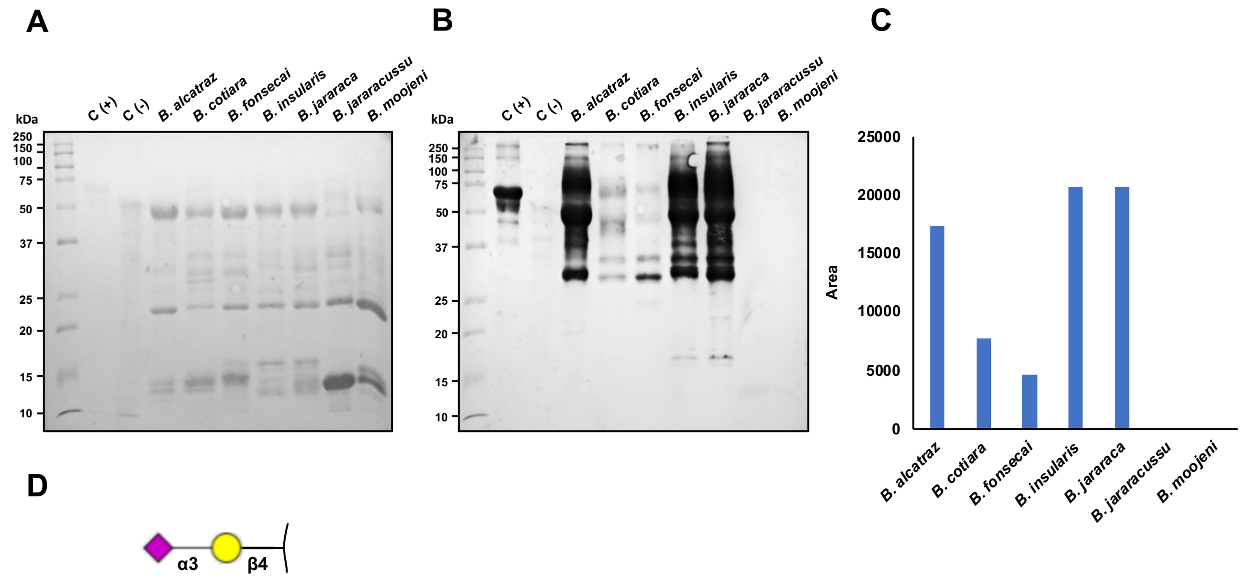

Supplementary Figure S3. Profile of proteins of *Bothrops* venoms recognized by lectin blot using digoxigenin-labeled MAA lectin. Proteins were separated in a 12.5% SDS-polyacrylamide gel under reducing conditions and transferred to a nitrocellulose membrane. (A) Membrane stained with ponceau S, used as the protein loading control. (B) Proteins detected by the lectin MAA. (C) Bar graph of the signal area observed in (B) and analyzed using Image Studio Lite version 5.2.5. C (+): positive control (3 µg of fetuin); C (-): negative control (3 µg of asialofetuin); venom protein mass loaded: 20 µg (*B. alcatraz*, *B. cotiara*, *B. fonsecai*, *B. insularis* and *B. jararaca*) or 40 µg (*B. jararacussu* and *B. moojeni*). (D) Structure recognized by MAA lectin.[44,125]

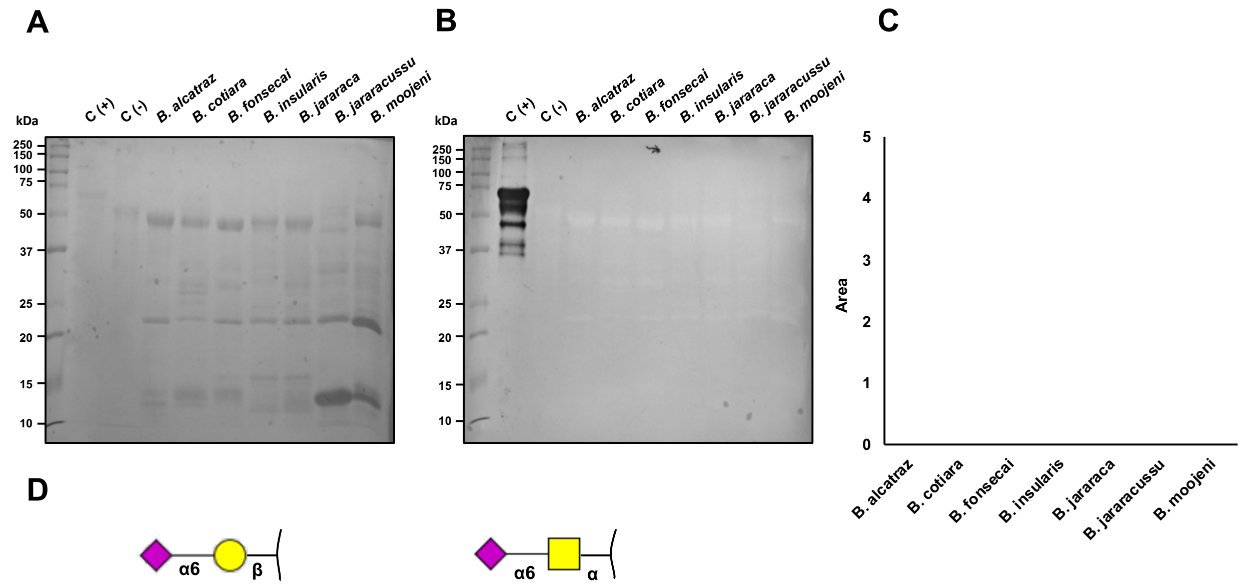

Supplementary Figure S4. Profile of proteins of *Bothrops* venoms recognized by lectin blot using digoxigenin-labeled SNA lectin. Proteins were separated in a 12.5% SDS-polyacrylamide gel under reducing conditions and transferred to a nitrocellulose membrane. (A) Membrane stained with ponceau S, used as the protein loading control. (B) Proteins detected by the lectin SNA. (C) Bar graph of the signal area observed in (B) and analyzed using Image Studio Lite version 5.2.5. C (+): positive control (3 µg of fetuin); C (-): negative control (3 µg of asialofetuin); venom protein mass loaded: 20 µg (*B. alcatraz*, *B. cotiara*, *B. fonsecai*, *B. insularis* and *B. jararaca*); 40 µg (*B. jararacussu* and *B. moojeni*). (D) Structures recognized by SNA lectin[44,126].

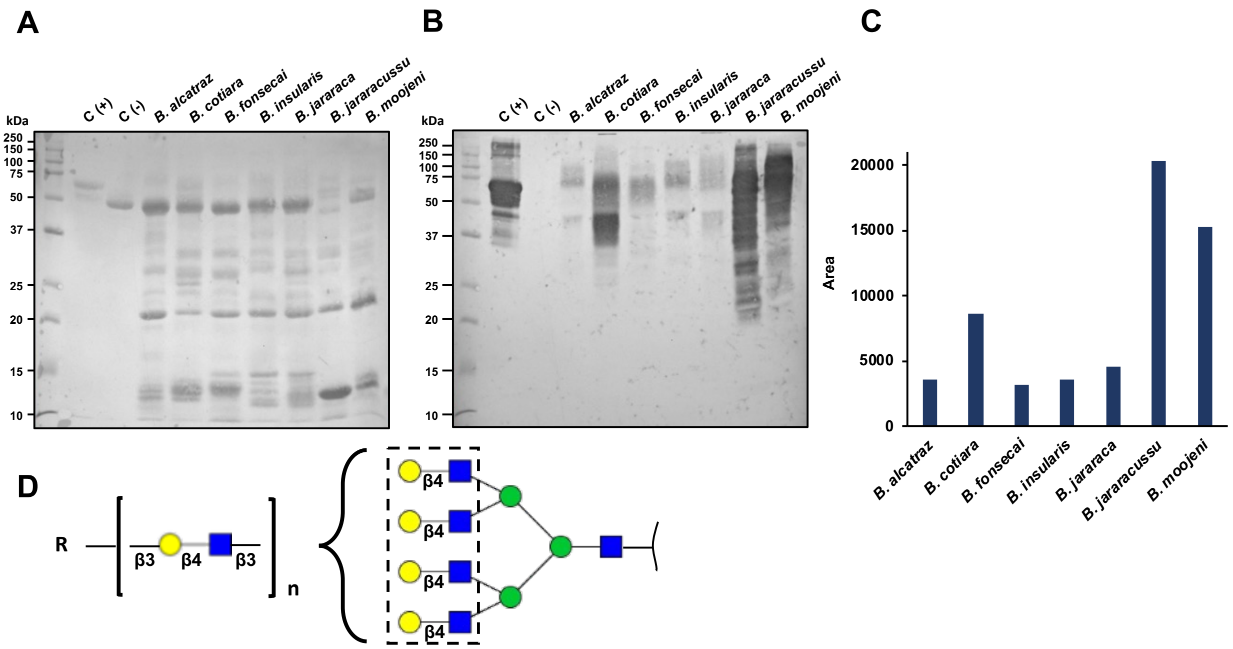

Supplementary Figure S5. Profile of proteins of *Bothrops* venoms recognized by lectin blot using digoxigenin-labeled DSA lectin. Proteins were separated in a 12.5% SDS-polyacrylamide gel under reducing conditions and transferred to a nitrocellulose membrane. (A) Membrane stained with Ponceau S, used as protein loading control. (B) Proteins detected by the lectin DSA. (C) Bar graph of the signal area observed in (B) and analyzed using Image Studio Lite version 5.2.5. C (+): positive control (3 μg of fetuin); C (-): negative control (3 μg of carboxypeptidase); venom protein mass loaded: 20 μg. (D) Structure recognized by DSA lectin[44].

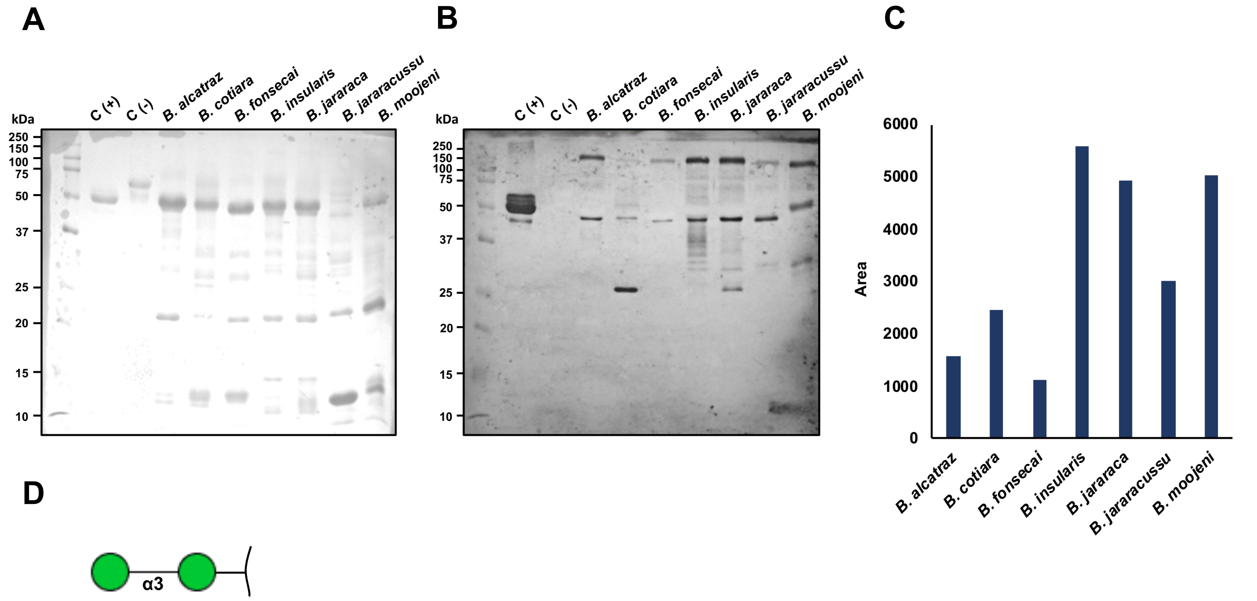

Supplementary Figure S6. Profile of the proteins of *Bothrops* venoms recognized by lectin blot using digoxigenin-labeled GNA lectin. Proteins were separated in a 12.5% SDS-polyacrylamide gel under reducing conditions and transferred to a nitrocellulose membrane. (A) Membrane stained with ponceau S, used as protein loading control. (B) Proteins detected by the lectin GNA. (C) Bar graph of the signal area observed in (B) and analyzed using Image Studio Lite version 5.2.5. C (+): positive control (3 µg of carboxypeptidase); C (-): negative control (3 µg of fetuin); venom protein mass loaded: 20 µg. (D) Structure recognized by GNA lectin[44].

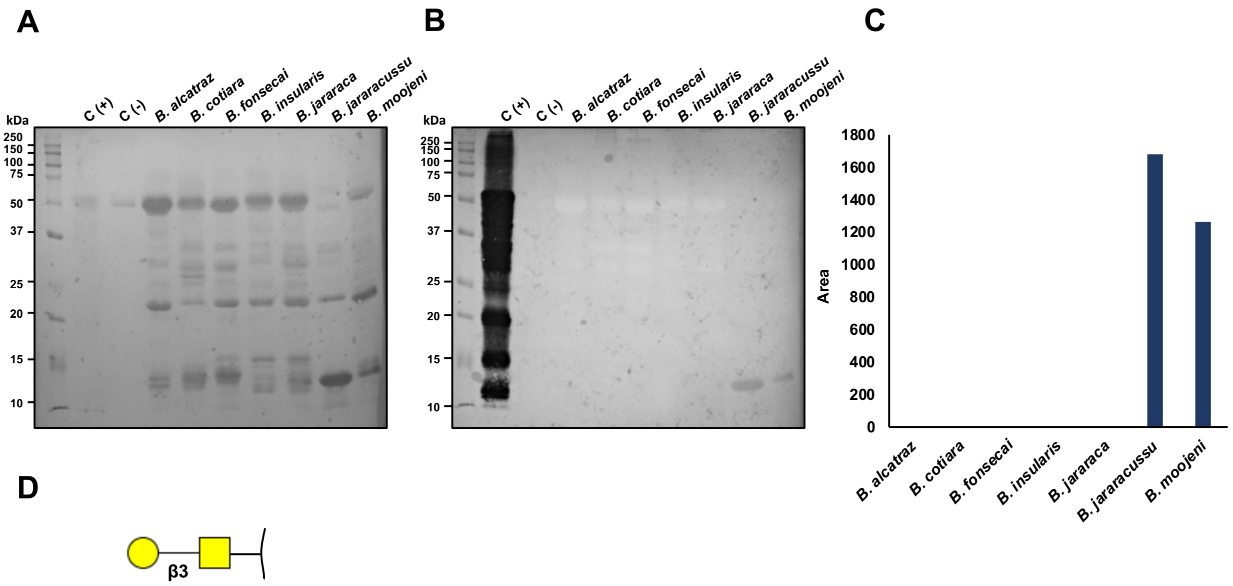

Supplementary Figure S7. Profile of proteins of *Bothrops* venoms recognized by lectin blot using digoxigenin-labeled PNA lectin. Proteins were separated in a 12.5% SDS-polyacrylamide gel under reducing conditions and transferred to a nitrocellulose membrane. (A) Membrane stained with ponceau S, used as the protein loading control. (B) Proteins detected by the lectin PNA. (C) Bar graph of the signal area observed in (B) and analyzed using Image Studio Lite version 5.2.5. C (+): positive control (3 µg of asialofetuin); C (-): negative control (3 µg of carboxypeptidase); venom protein mass loaded: 30 µg. (D) Structure recognized by PNA lectin[44].

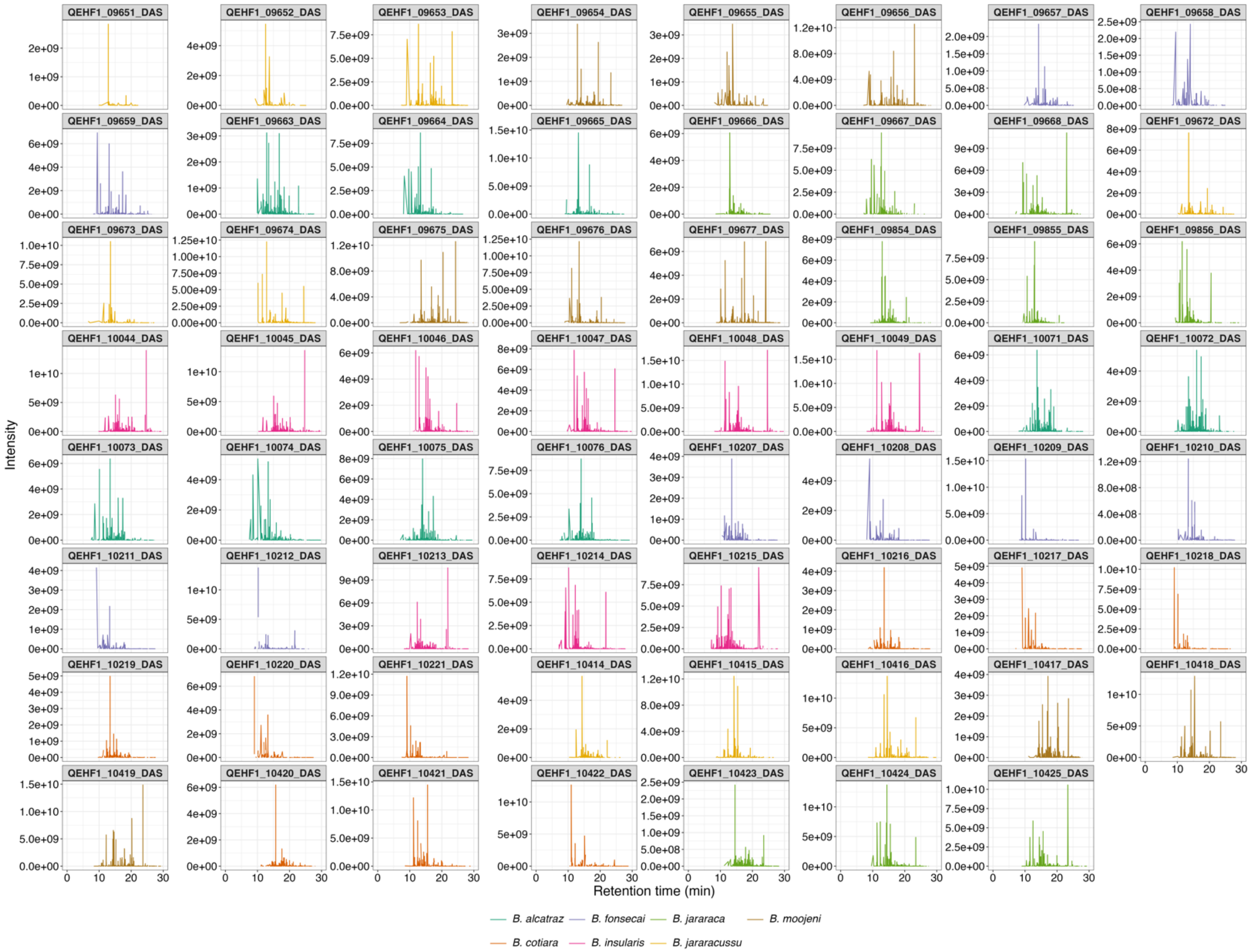
QEHF1_09651_DAS
QEHF1_09652_DAS
QEHF1_09653_DAS
QEHF1_09654_DAS
QEHF1_09655_DAS
QEHF1_09656_DAS
QEHF1_09657_DAS
QEHF1_09658_DAS
QEHF1_09659_DAS
QEHF1_09663_DAS
QEHF1_09664_DAS
QEHF1_09665_DAS
QEHF1_09666_DAS
QEHF1_09667_DAS
QEHF1_09668_DAS
QEHF1_09672_DAS
QEHF1_09673_DAS
QEHF1_09674_DAS
QEHF1_09675_DAS
QEHF1_09676_DAS
QEHF1_09677_DAS
QEHF1_09854_DAS
QEHF1_09855_DAS
QEHF1_09856_DAS
QEHF1_10044_DAS
QEHF1_10045_DAS
QEHF1_10046_DAS
QEHF1_10047_DAS
QEHF1_10048_DAS
QEHF1_10049_DAS
QEHF1_10071_DAS
QEHF1_10072_DAS
QEHF1_10073_DAS
QEHF1_10074_DAS
QEHF1_10075_DAS
QEHF1_10076_DAS
QEHF1_10207_DAS
QEHF1_10208_DAS
QEHF1_10209_DAS
QEHF1_10210_DAS
QEHF1_10211_DAS
QEHF1_10212_DAS
QEHF1_10213_DAS
QEHF1_10214_DAS
QEHF1_10215_DAS
QEHF1_10216_DAS
QEHF1_10217_DAS
QEHF1_10218_DAS
QEHF1_10219_DAS
QEHF1_10220_DAS
QEHF1_10221_DAS
QEHF1_10414_DAS
QEHF1_10415_DAS
QEHF1_10416_DAS
QEHF1_10417_DAS
QEHF1_10418_DAS
QEHF1_10419_DAS
QEHF1_10420_DAS
QEHF1_10421_DAS
QEHF1_10422_DAS
QEHF1_10423_DAS
QEHF1_10424_DAS
QEHF1_10425_DAS
Intensity
Retention time (min)
B. alcatraz
B. fonsecai
B. jararaca
B. moojeni
B. cotiara
B. insularis
B. jararacussu

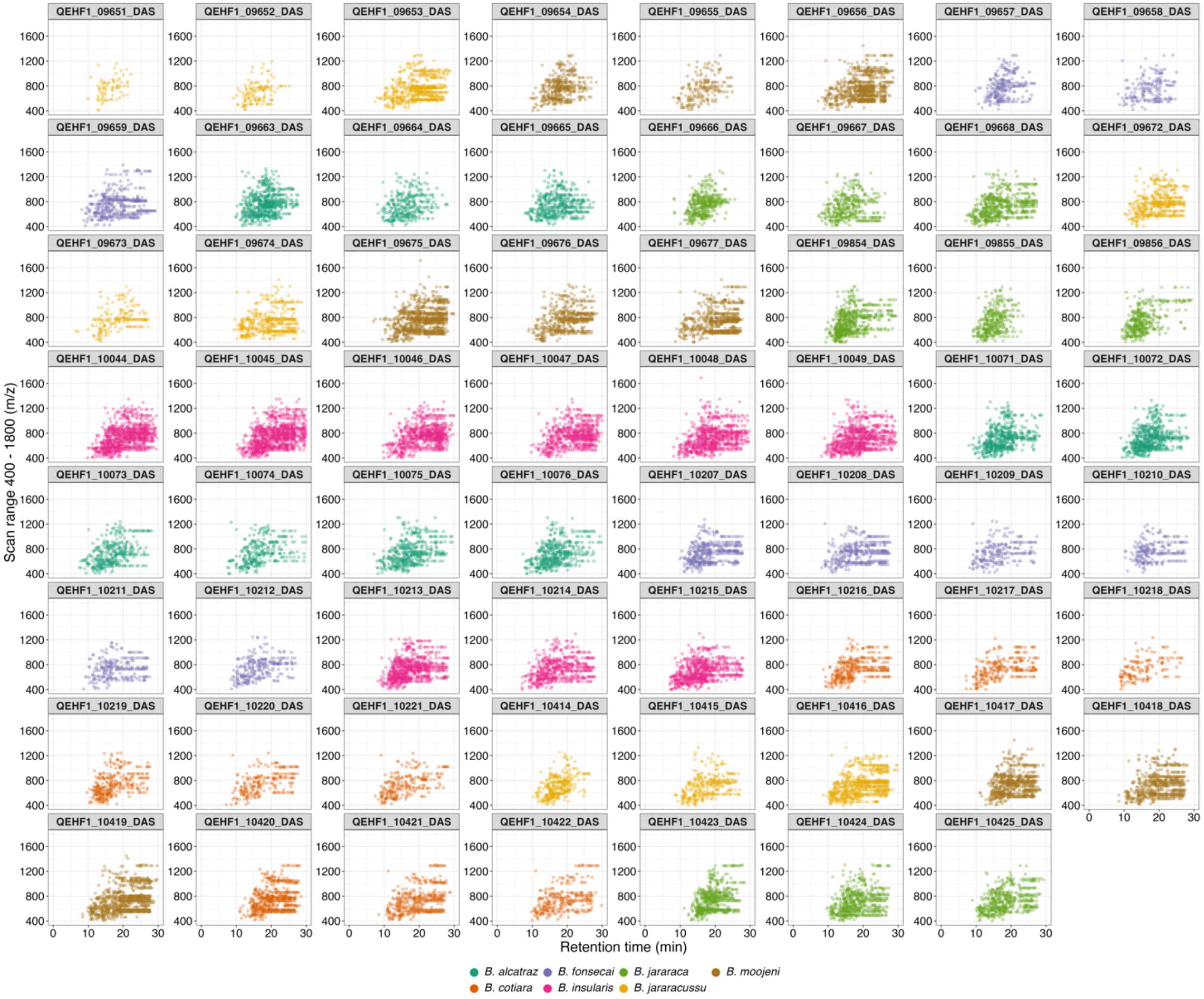

Supplementary Figure S8. Extracted ion chromatograms (upper panel) and distribution of peptide m/z values (lower panel) of LC-MS/MS analysis of de-*N*-glycosylated fractions (dF1, dF2 and dF3) of *Bothrops* venoms.

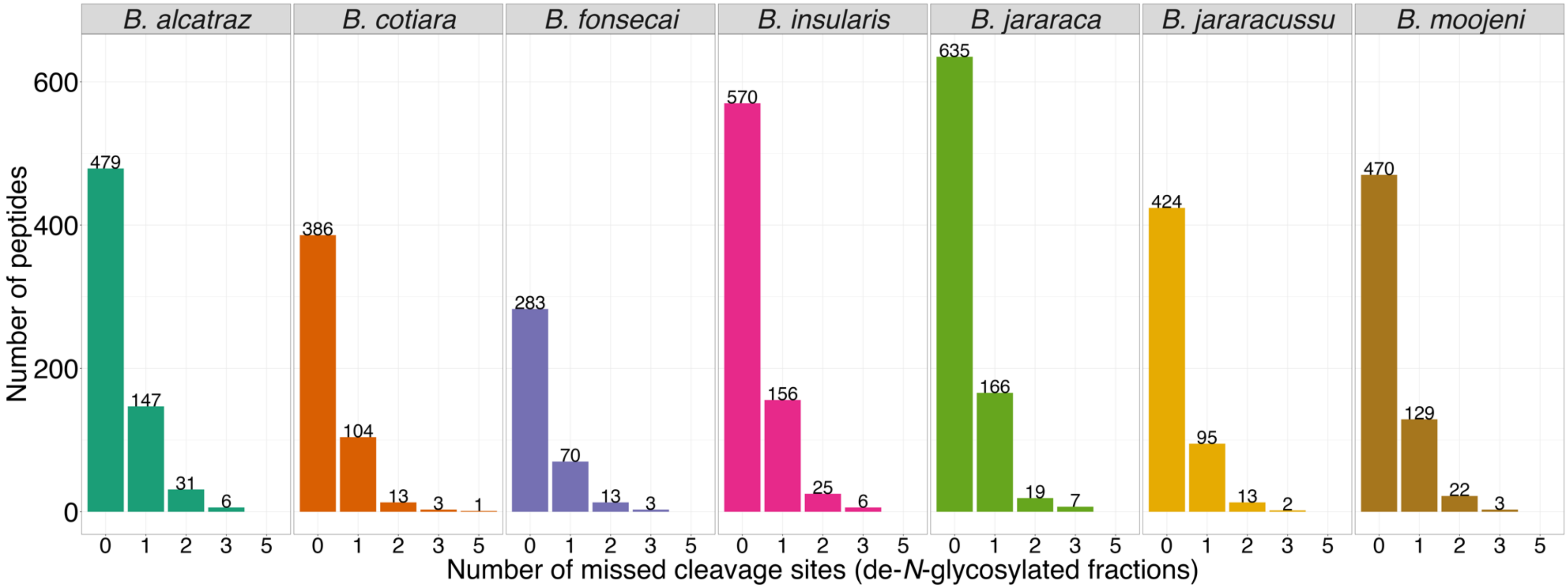

Supplementary Figure S9. Numbers of missed cleavages detected in LC-MS/MS analysis of de-*N*-glycosylated fractions (dF1, dF2 and dF3) of *Bothrops* venoms.

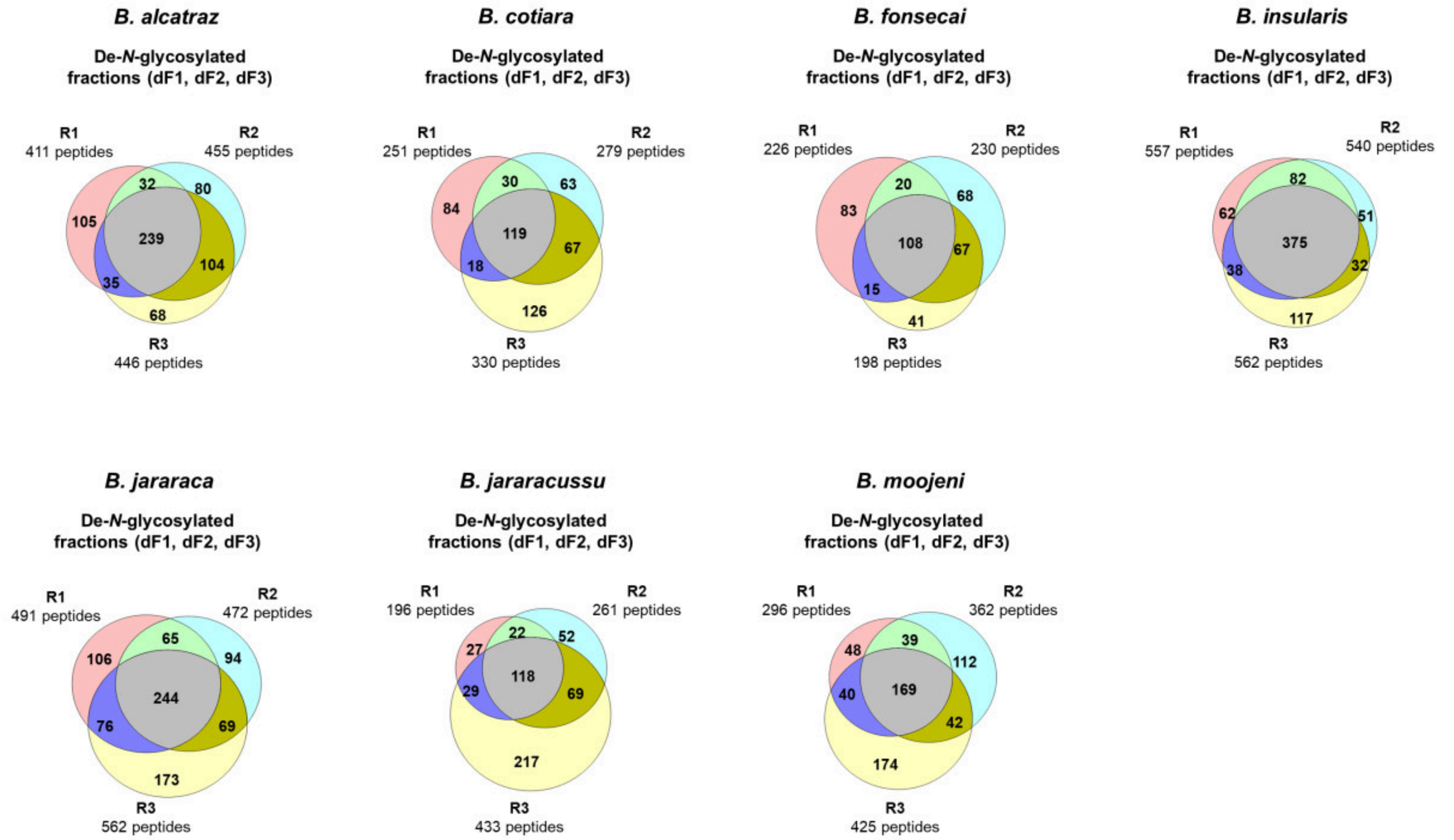

Supplementary Figure S10. Venn diagrams of the number of peptides identified in the three replicates of de-*N*-glycosylated fractions (dF1, dF2 and dF3) of *Bothrops* venoms.

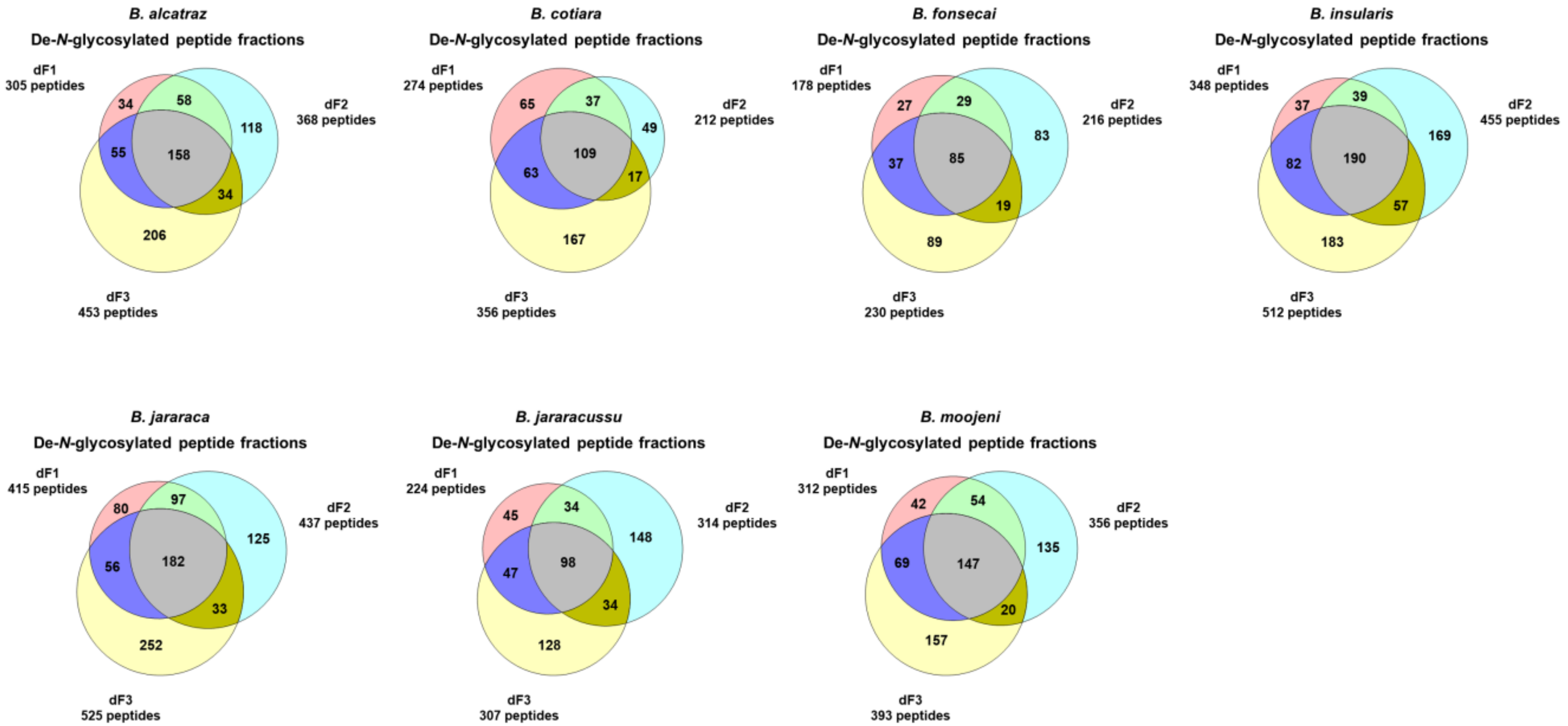

Supplementary Figure S11. Venn diagrams of the number of peptides identified in each of the three replicates of de-*N*-glycosylated fractions (dF1, dF2 and dF3) of *Bothrops* venoms. The numbers outside the circles indicate the total of peptides identified in each fraction.

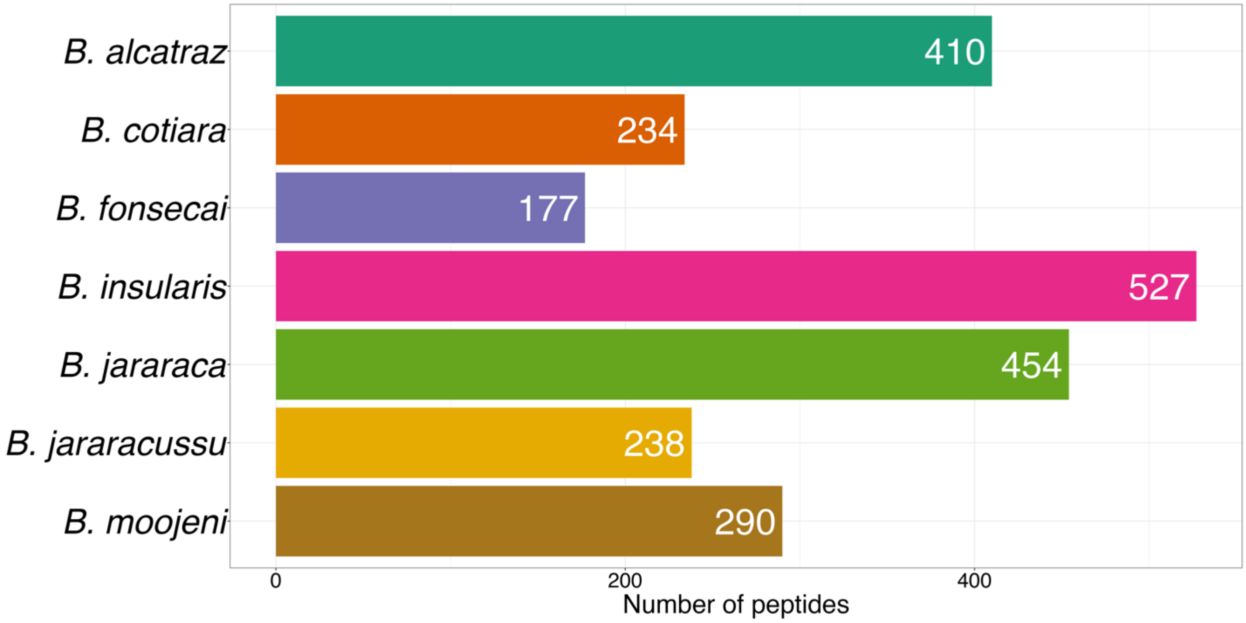

Supplementary Figure S12. Number of peptides identified in at least two replicates of LC-MS/MS analysis of de-*N*-glycosylated fractions (dF1, dF2 and dF3) of *Bothrops* venoms.

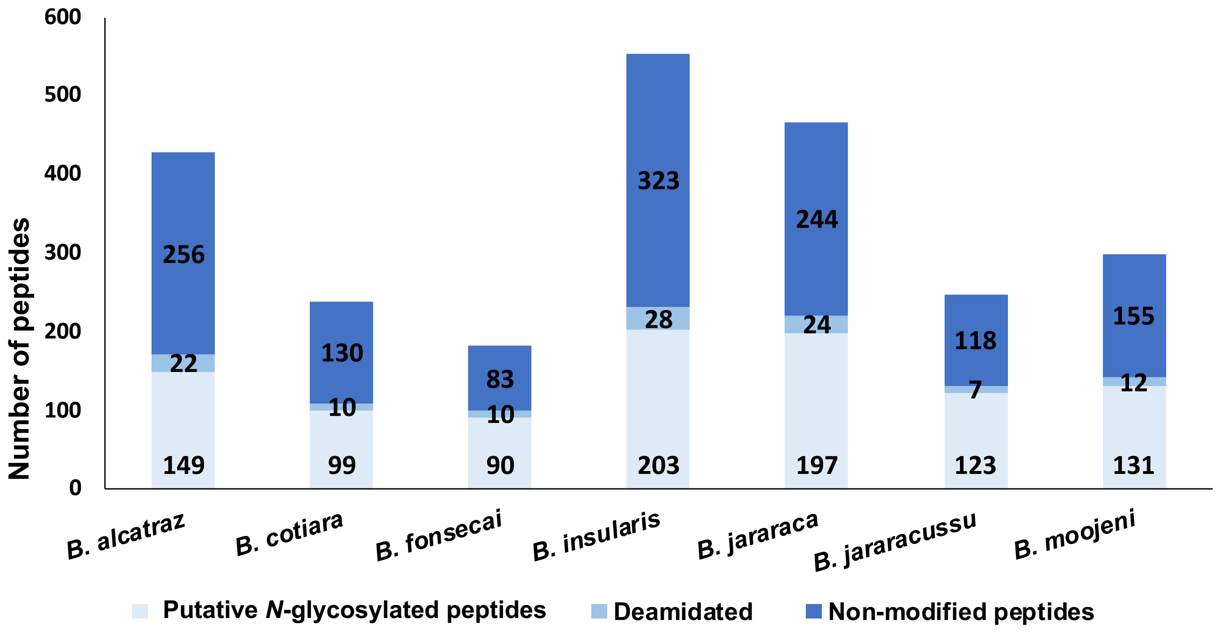

Supplementary Figure S13. Summary of peptide identifications in the de-*N*-glycosylated fractions (dF1, dF2, and dF3) of *Bothrops* venoms. Bar graph of the combined number of peptides identified in fractions dF1, dF2, and dF3, in at least two technical replicates. The identified peptides were classified according to the presence of a deamidation event: 'putative *N*-glycosylated peptides' are deamidated peptides that contain at least one asparagine residue within the consensus sequence (NXS/T); 'deamidated peptides', are those with a deamidation event but no glycosylation consensus sequence; and 'non-modified peptides' are those identified with no observed deamidation event.

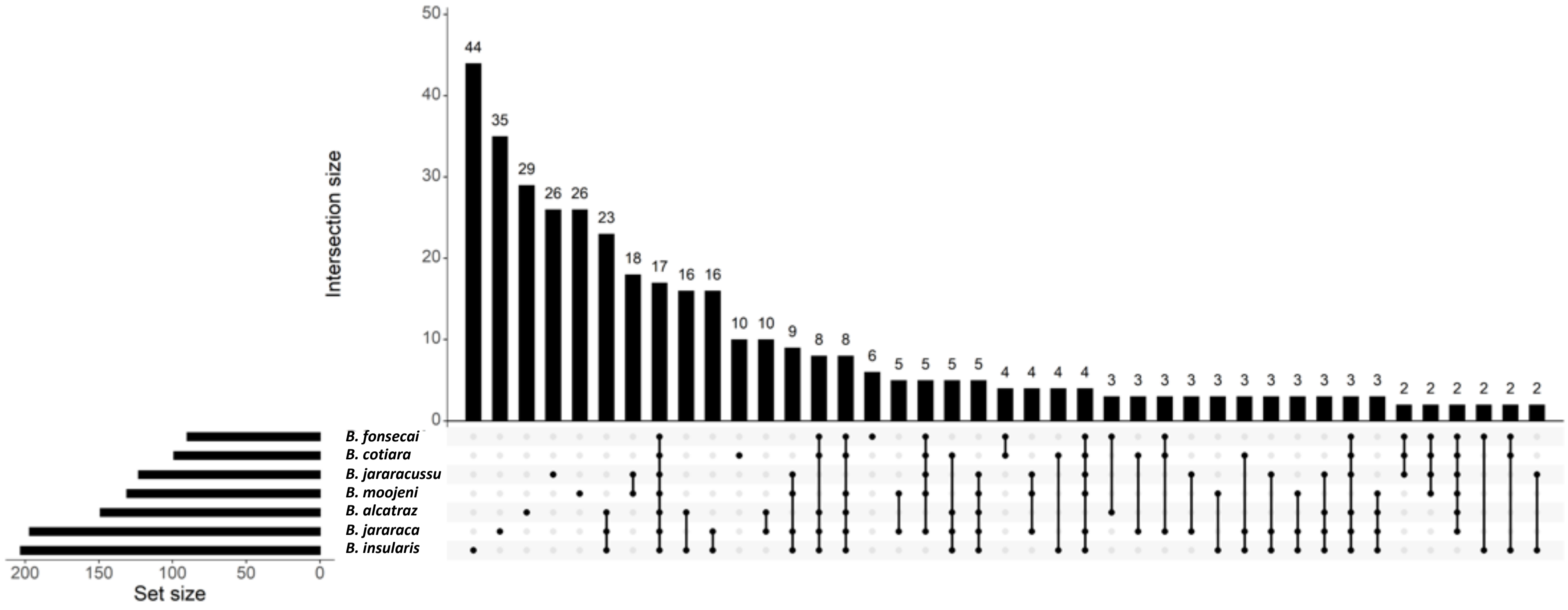

Supplementary Figure S14. Intersections of identified putative *N*-glycosylated peptide sequences in *Bothrops* venoms. UpSet plot of putative *N*-glycosylated peptides identified in the de-*N*-glycosylated fractions. Y-axis: numbers of peptides (unique or shared). Horizontal bars represent the total number of identified sequences in each venom. Connections between the circles represent peptides shared by the venoms. Unconnected circles represent unique peptides.

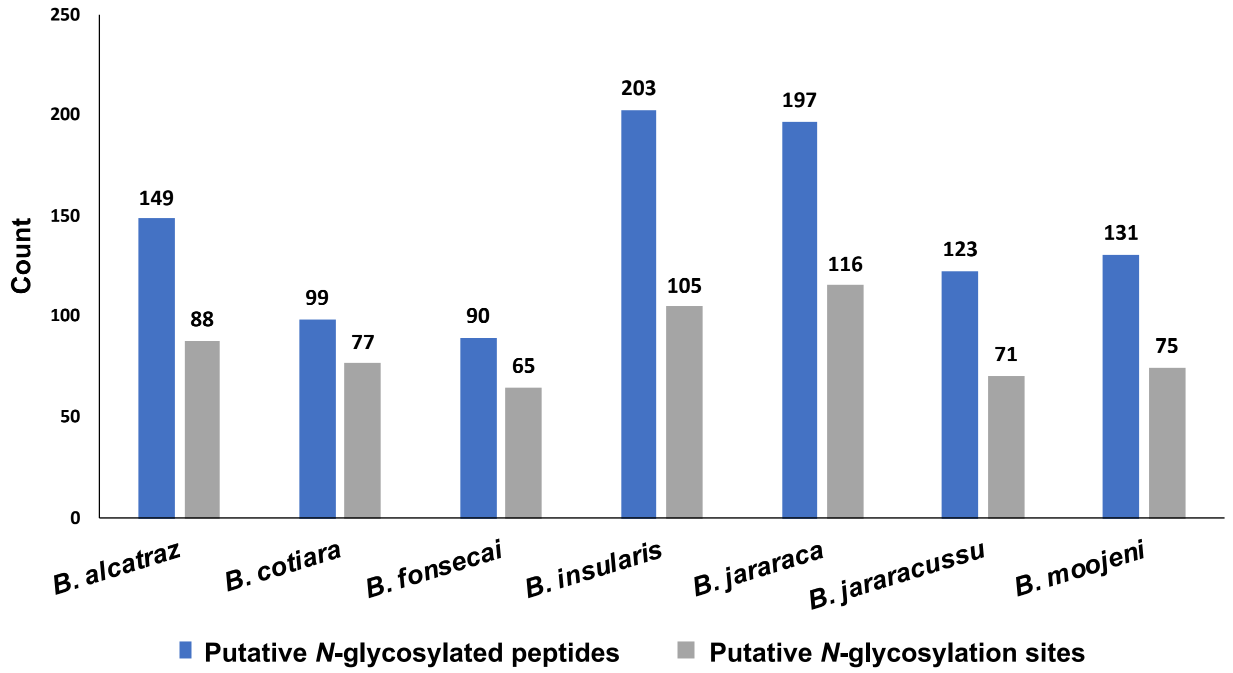

Supplementary Figure S15. Comparison of the number of putative *N*-glycosylated peptides and putative *N*-glycosylation sites identified in de-*N*-glycosylated fractions (Supplementary Tables 11−17).

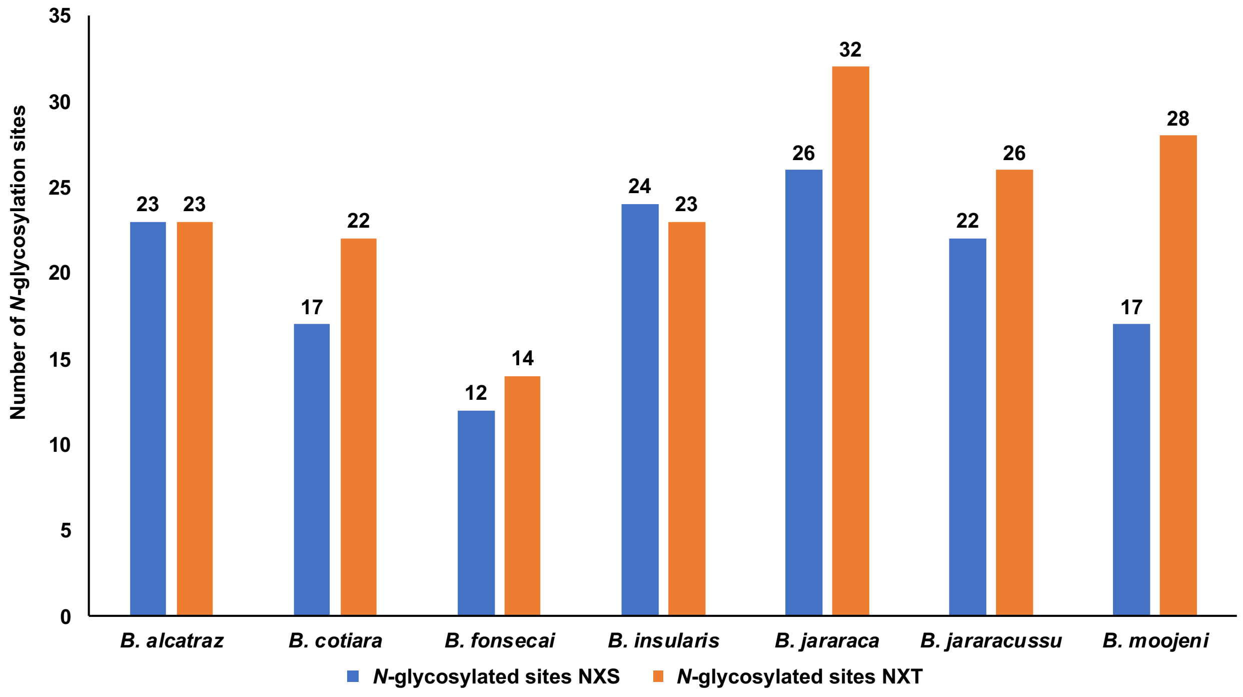

Supplementary Figure S16. Number of the *N*-glycosylation sites containing the sequons NXS and NXT identified in the intact *N*-glycosylated peptides.

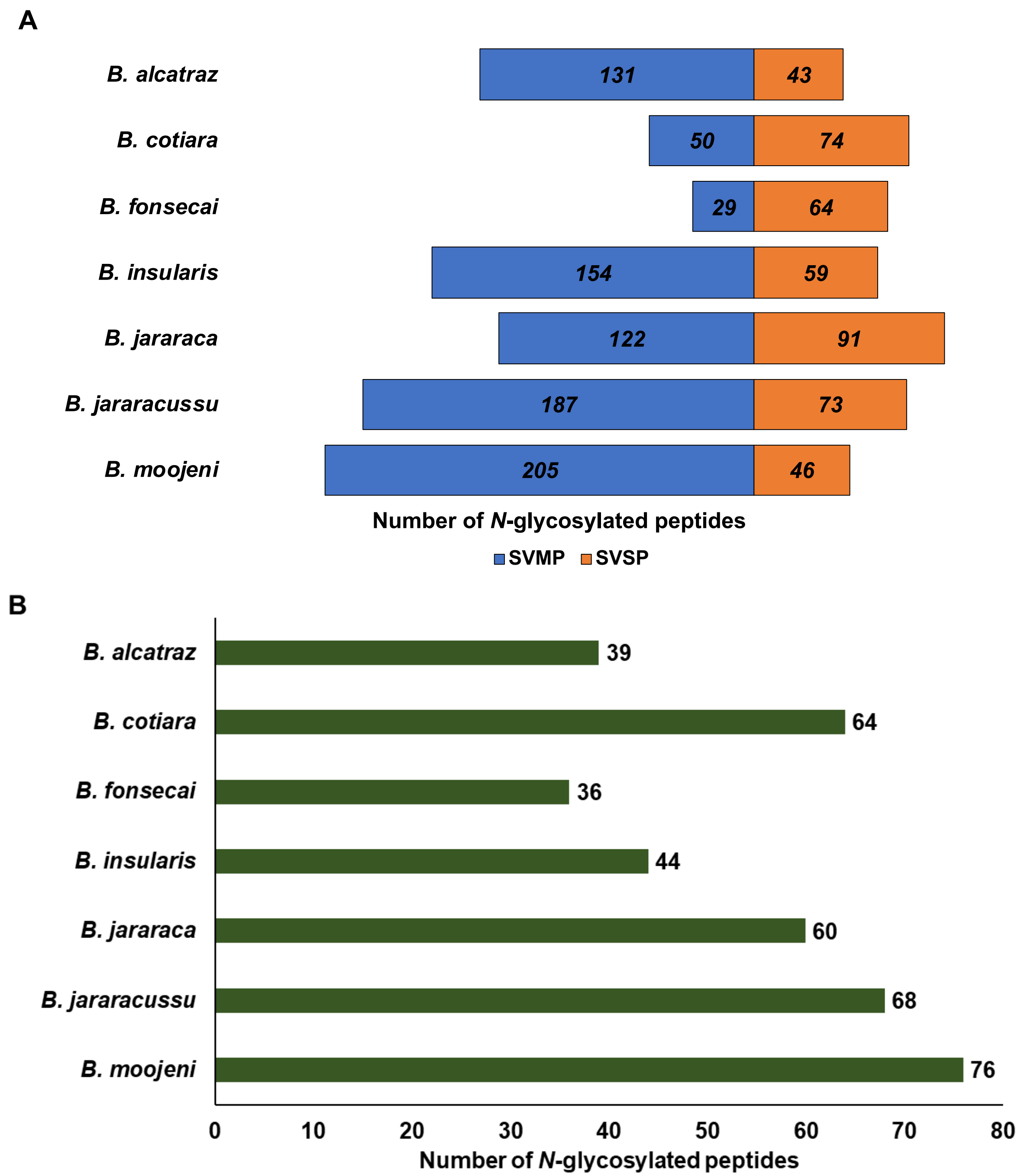

Supplementary Figure S17. Analysis of the distribution of *N*-glycosylated peptides identified in the intact glycopeptide fractions, according to toxin class and *Bothrops* species. (A) Counting of the number of *N*-glycosylated peptides belonging to SVMPs and SVSPs. (B) Counting of the number of *N*-glycosylated peptides belonging to other toxin classes.

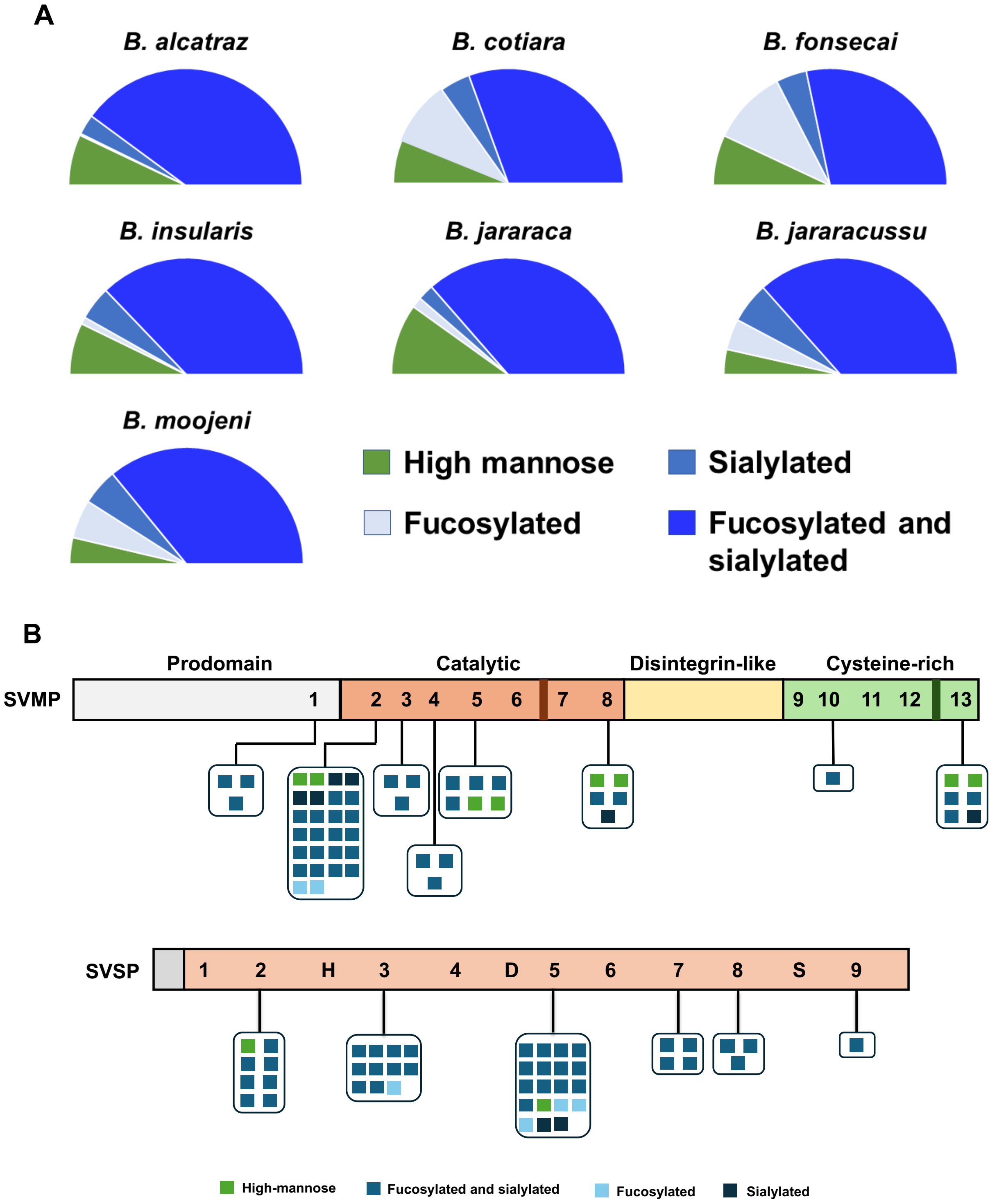

Supplementary Figure S18. Analysis of *N*-glycan classes identified in *Bothrops* venoms. (A) Analysis of the proportions of *N*-glycans classified as high-mannose, fucosylated, sialylated, and fucosylated and sialylated, identified in the intact *N*-glycosylated peptides of *Bothrops* venoms. (B) Identification of occupied *N*-glycosylation sites in a typical P-III SVMP and in a SVSP.

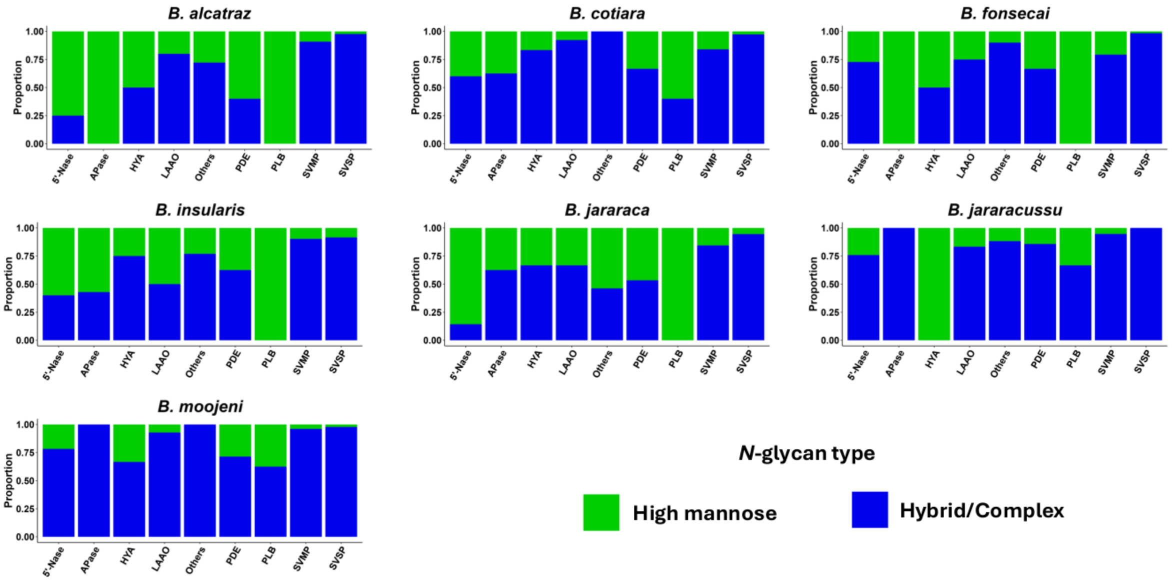

Supplementary Figure S19. Distribution of intact *N*-glycosylated peptides identified in *Bothrops* venoms, by toxin class, and the proportion of identified peptides that contain *N*-glycan chains of high-mannose or hybrid/complex types.

N-glycopeptide from *B. insularis* **YTGNSTAIR** + Fuc:1; Hex:4; HexNAc:3; Neu5Ac:1; Neu5Gc:1

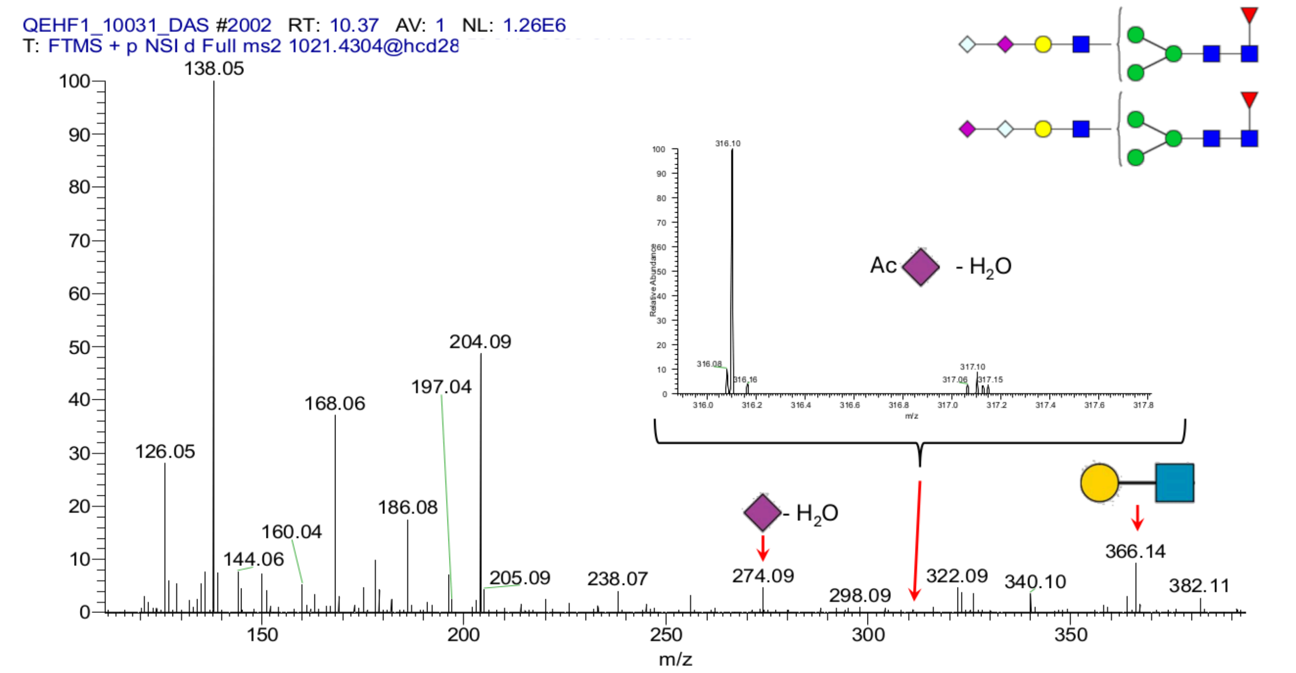

Supplementary Figure S20. MS/MS spectra of an intact acetylated *N*-glycopeptide identified in *B. insularis* venom. The detection of the ion 316.1^{+1} indicates the presence of an acetylated NeuAc (Neu5Ac$_2$).

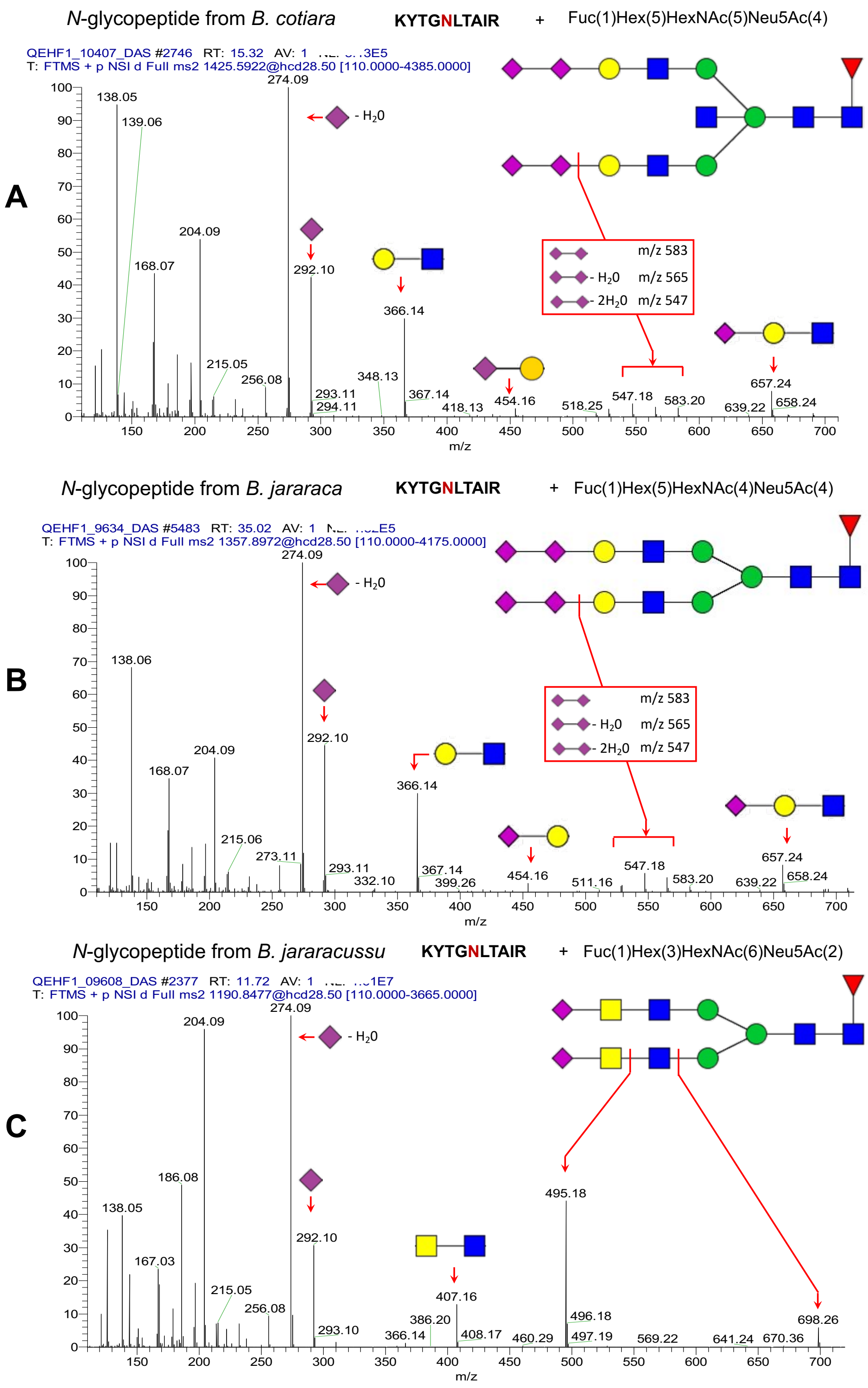

Supplementary Figure S21. Comparison of the low mass range of MS/MS spectra of an intact *N*-glycopeptide identified in *B. cotiara*, *B. jararaca*, and *B. jararacussu* venoms. (A) Fragmentation of the antenna present in an *N*-glycopeptide of *B. cotiara* venom. (B) Fragmentation of the antenna present in an *N*-glycopeptide of *B. jararaca* venom. (C) Fragmentation of the antenna present in an *N*-glycopeptide of *B. jararacussu* venom.

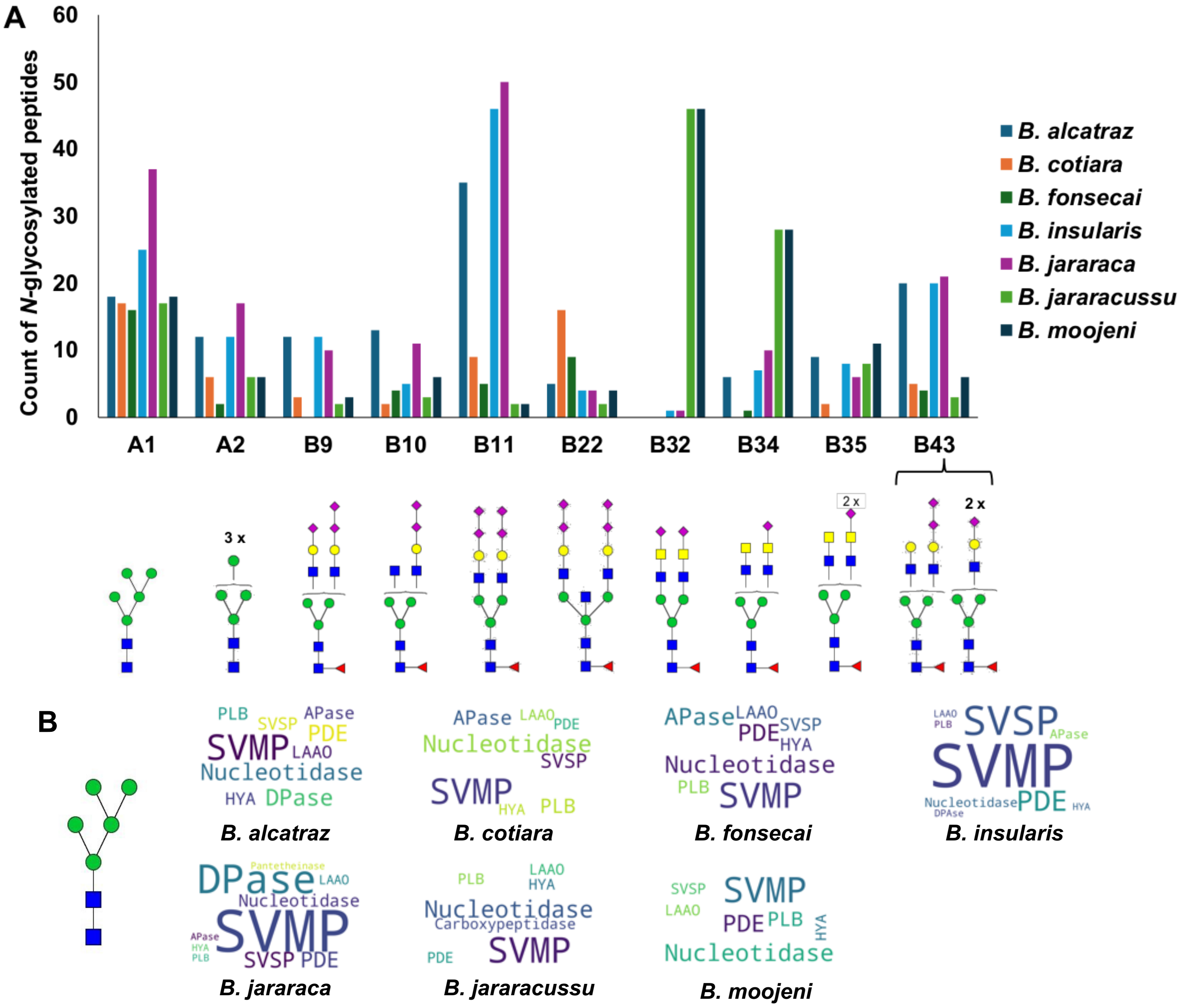

Supplementary Figure S22. Overview of the most often *N*-glycan compositions identified in the seven *Bothrops* venoms analyzed in this study. (A) Comparison of the number of intact *N*-glycopeptides identified as containing the ten most frequent *N*-glycan compositions among the *Bothrops* venoms. Below the graph the structures identified for these *N*-glycan compositions in Andrade-Silva *et al.*[15] are shown. (B) Word clouds of the most frequent toxin classes in which *N*-glycosylated peptides containing the A1 composition were identified. Word clouds were generated using https://chartexpo.com/wordcloud.

A

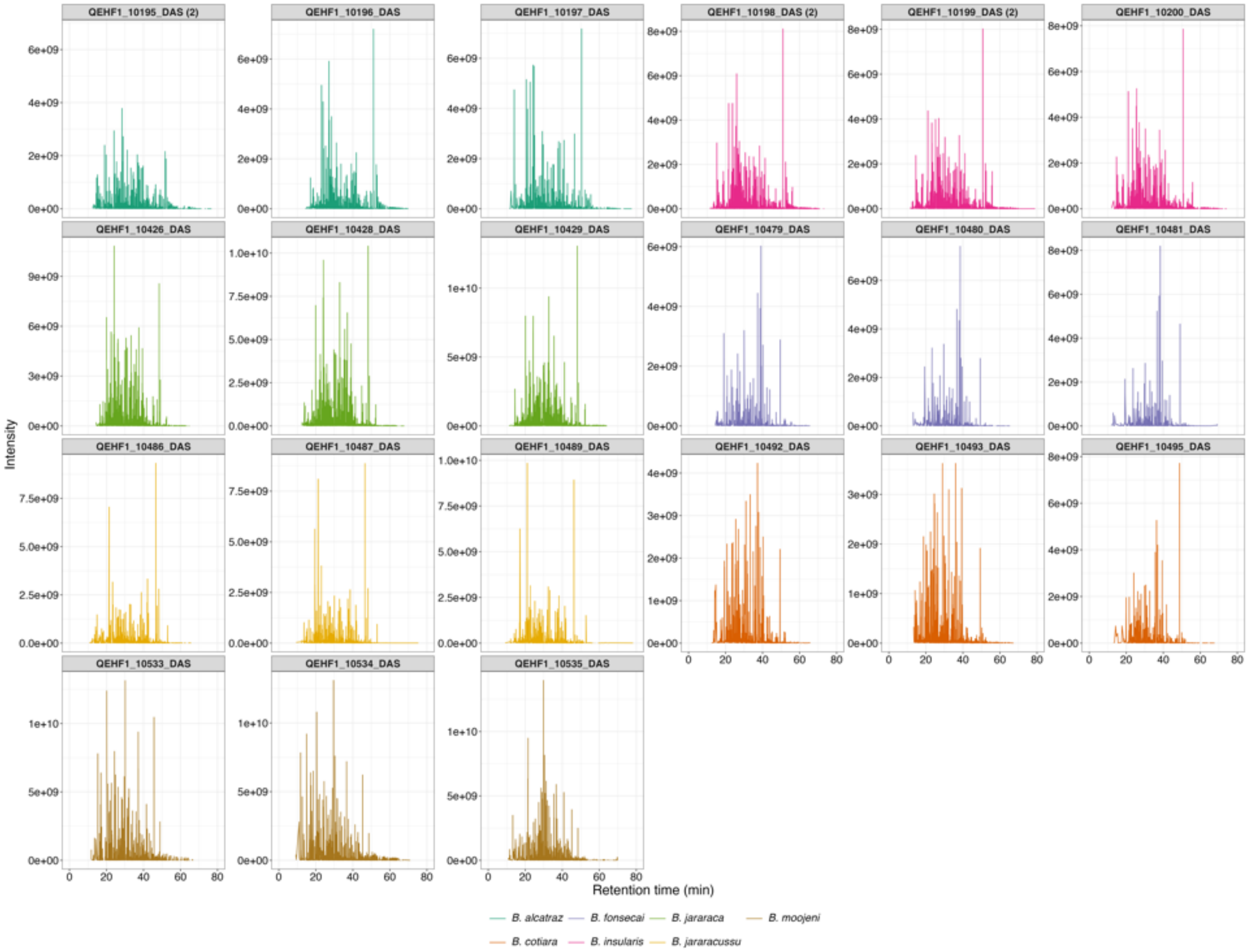

QEHF1_10195_DAS (2)
QEHF1_10196_DAS
QEHF1_10197_DAS
QEHF1_10198_DAS (2)
QEHF1_10199_DAS (2)
QEHF1_10200_DAS
QEHF1_10426_DAS
QEHF1_10428_DAS
QEHF1_10429_DAS
QEHF1_10479_DAS
QEHF1_10480_DAS
QEHF1_10481_DAS
QEHF1_10486_DAS
QEHF1_10487_DAS
QEHF1_10489_DAS
QEHF1_10492_DAS
QEHF1_10493_DAS
QEHF1_10495_DAS
QEHF1_10533_DAS
QEHF1_10534_DAS
QEHF1_10535_DAS
Intensity
Retention time (min)
B. alcatraz
B. fonsecai
B. jararaca
B. moojeni
B. cotiara
B. insularis
B. jararacussu

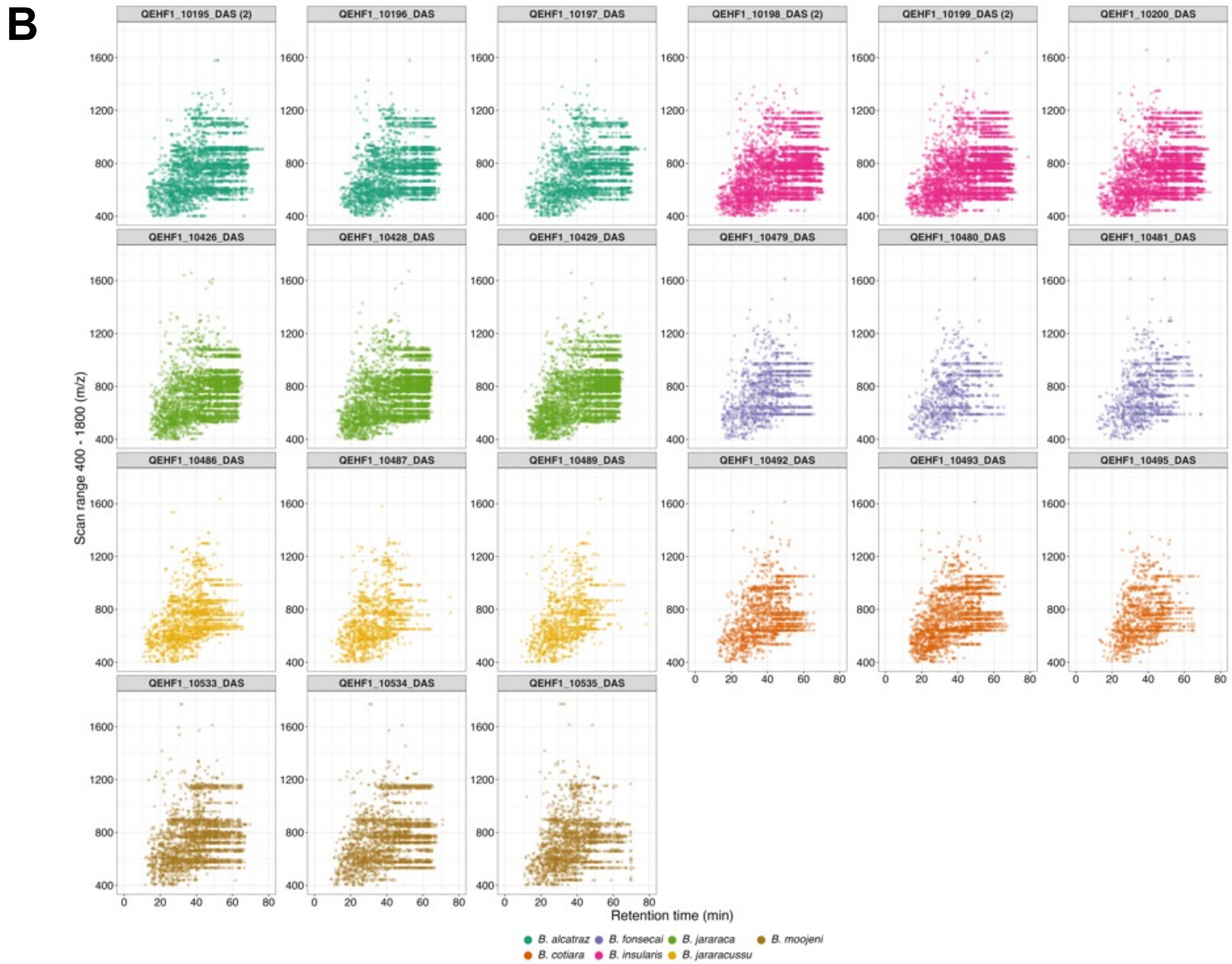

Supplementary Figure S23. Extracted ion chromatograms (upper panel) and distribution of peptide m/z values (lower panel) of LC-MS/MS analysis of non-glycosylated fractions of *Bothrops* venoms.

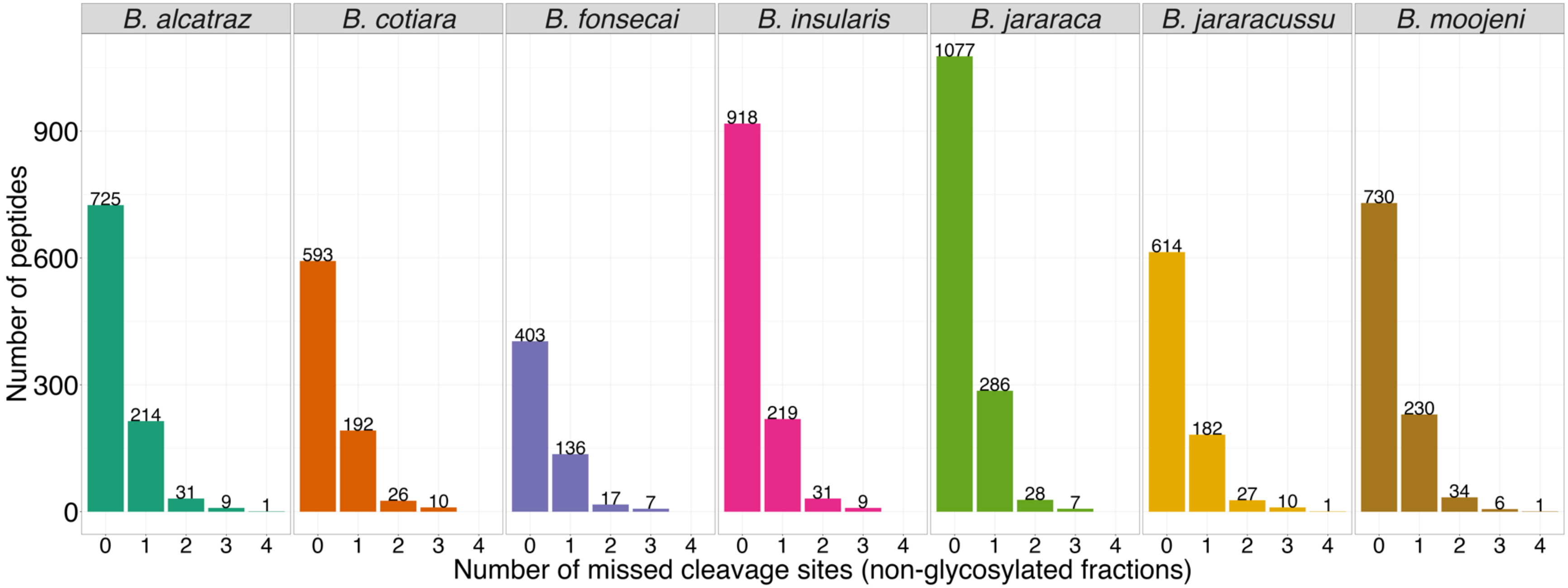

Supplementary Figure S24. Numbers of missed cleavages detected in LC-MS/MS analysis of non-glycosylated fractions of *Bothrops* venoms.

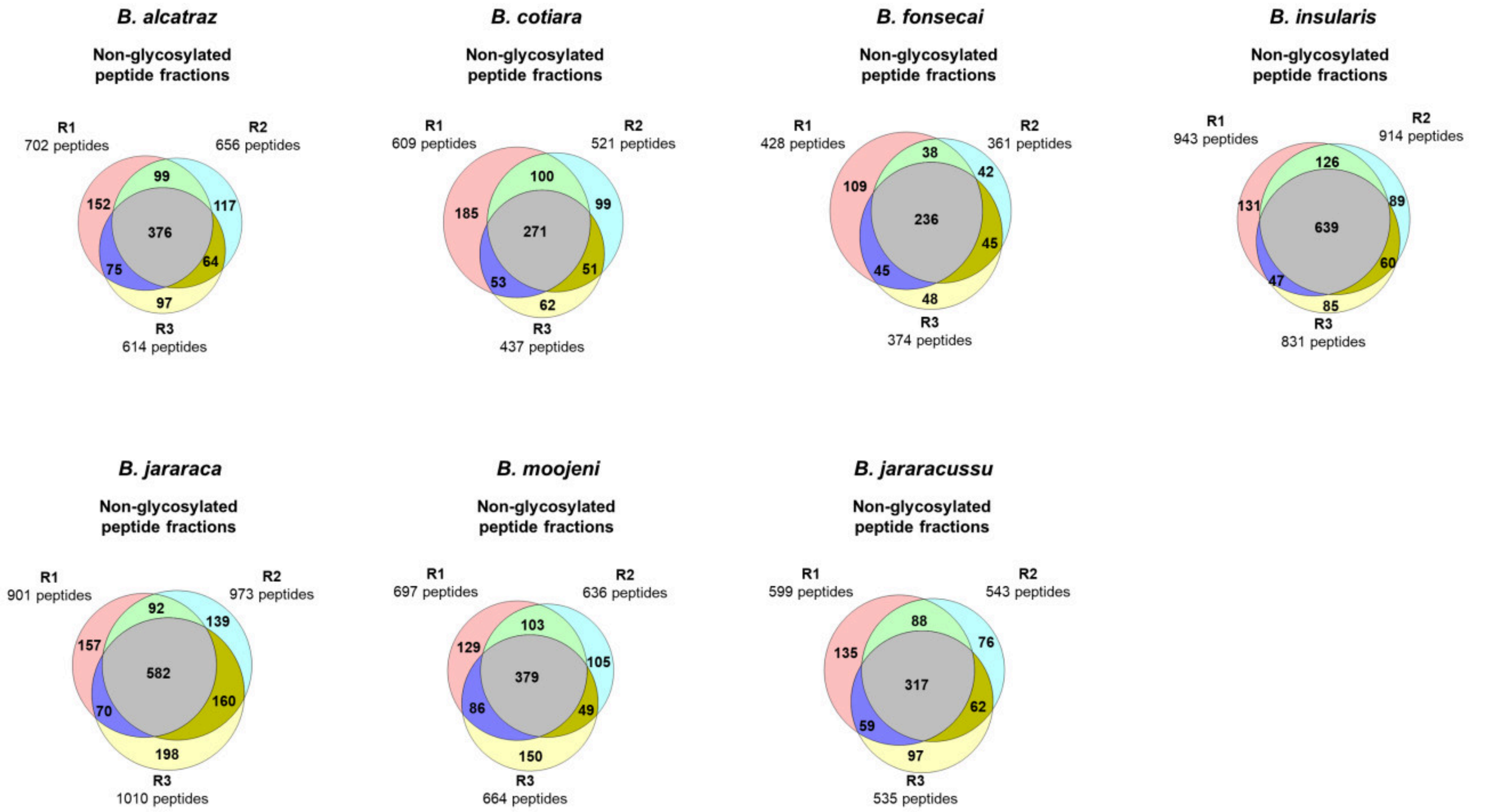

Supplementary Figure S25. Venn diagrams of the number of peptides identified in the three replicates of non-glycosylated peptide fractions of *Bothrops* venoms.

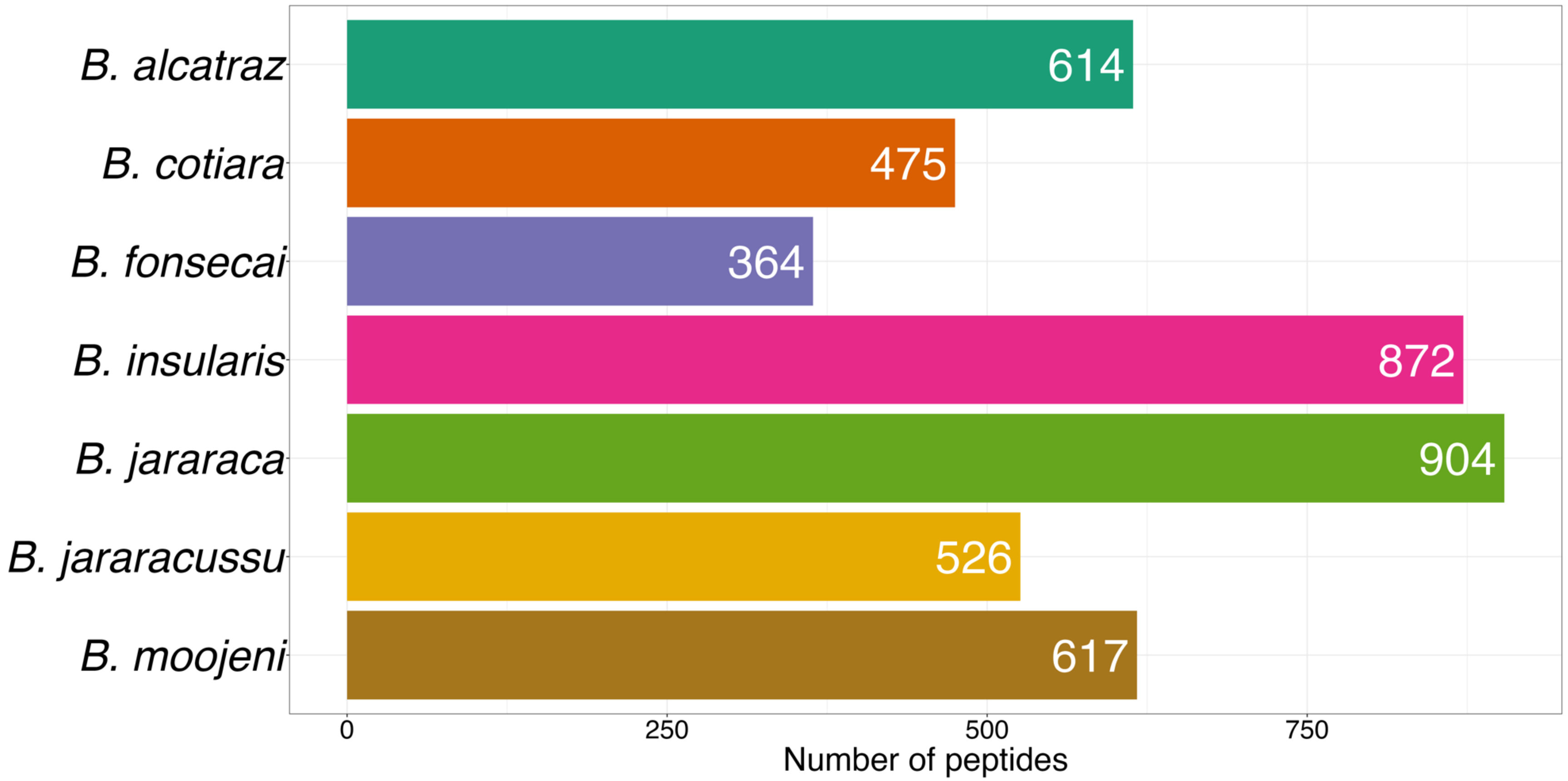

Supplementary Figure S26. Number of peptides identified in at least two replicates of LC-MS/MS analysis of non-glycosylated fractions of *Bothrops* venoms.

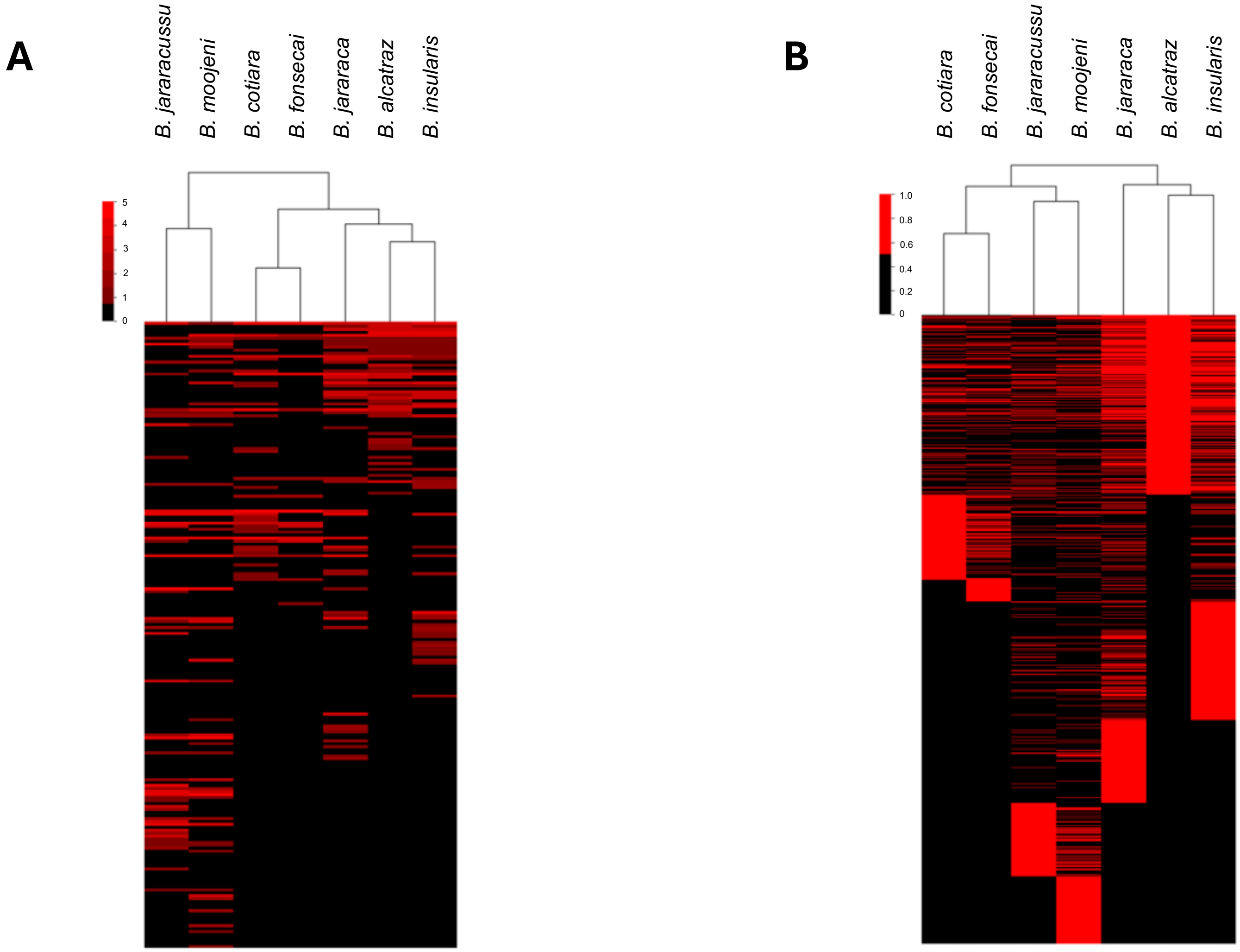

Supplementary Figure S27. *Bothrops* venom clustering according to the amino acid backbones of glycosylated peptides (A) or non-glycosylated peptides (B).

References

119. Bradford MM. A rapid and sensitive method for the quantitation of microgram quantities of protein utilizing the principle of protein-dye binding. Anal Biochem. 1976;72:248-254. doi:10. Bradford MM. 1006/abio.1976.9999

120. Laemmli UK. Cleavage of structural proteins during the assembly of the head of bacteriophage T4. Nature. 1970;227(5259):680-685. doi:10.1038/227680a0

121. DuBois Michel, Gilles KA, Hamilton JK, Rebers PA, Smith Fred. Colorimetric method for determination of sugars and related substances. Anal Chem. 1956;28(3):350-356. doi:10.1021/ac60111a017

122. Larsen MR, Jensen SS, Jakobsen LA, Heegaard NHH. Exploring the sialiome using titanium dioxide chromatography and aass spectrometry. Mol Cell Proteomics. 2007;6(10):1778-1787. doi:10.1074/mcp.M700086-MCP200

123. Klein J, Zaia J. Relative retention time estimation improves N-glycopeptide identifications by LC–MS/MS. J Proteome Res. 2020;19(5):2113-2121. doi:10.1021/acs.jproteome.0c00051

124. Chambers MC, Maclean B, Burke R, Amodei D, Ruderman DL, Neumann S, Gatto L, Fischer B, Pratt B, Egertson J, Hoff K, Kessner D, Tasman N, Shulman N, Frewen B, Baker TA, Brusniak MY, Paulse C, Creasy D, Flashner L, Kani K, Moulding C, Seymour SL, Nuwaysir LM, Lefebvre B, Kuhlmann F, Roark J, Rainer P, Detlev S, Hemenway T, Huhmer A, Langridge J, Connolly B, Chadick T, Holly K, Eckels J, Deutsch EW, Moritz RL, Katz JE, Agus DB, MacCoss M, Tabb DL, Mallick P. A cross-platform toolkit for mass spectrometry and proteomics. Nat Biotechnol. 2012;30(10):918-920. doi:10.1038/nbt.2377

125. Bojar D, Meche L, Meng G, Eng W, Smith DF, Cummings RD, Mahal LK. A Useful guide to lectin binding: machine-learning directed annotation of 57 unique lectin specificities. ACS Chem Biol. 2022;17(11):2993-3012. doi:10.1021/acschembio.1c00689
